# Integrative Bioinformatics Prioritizes the *TLR4* Axis and Candidate Non-Starch Polysaccharides in Hyperuricemia-Associated Inflammation

**DOI:** 10.3390/biology15141150

**Published:** 2026-07-14

**Authors:** Pengcheng You, Anye Chen, Qiancheng Feng, Junhong Hou, Jiacheng Zheng, Hao Chen

**Affiliations:** 1 SDU-ANU Joint Science College, Shandong University, No. 180 West Wenhua Road, Weihai 264209, China; youpengcheng@mail.sdu.edu.cn (P.Y.); anyechen@mail.sdu.edu.cn (A.C.); 202300700273@mail.sdu.edu.cn (Q.F.); 2 Marine College, Shandong University, No. 180 West Wenhua Road, Weihai 264209, China; 202500810054@mail.sdu.edu.cn (J.H.); karcenzheng@yeah.net (J.Z.); 3 Shandong Key Laboratory of Intelligent Marine Engineering Geology, Environment and Equipment, Qingdao 266237, China

**Keywords:** hyperuricemia, Toll-like receptor 4, fucoidan, alginate, bioinformatics

## Abstract

Hyperuricemia (HUA) is a common immunometabolic disorder associated with gout, renal dysfunction, and systemic inflammation, yet the molecular targets through which non-starch polysaccharides (NSPs) may modulate HUA-related inflammation remain unclear. Here, we applied an integrative bioinformatics and computational workflow combining public transcriptomic datasets, curated NSP-related targets, protein–protein interaction analysis, enrichment analysis, single-cell RNA sequencing, and Mendelian randomization. We further included GutMGene-based orthogonal support analysis, guided docking, structural dynamics analysis, exploratory ADMET profiling, and in silico *TLR4* knockout to extend target prioritization. This approach prioritized a *TLR4*-centered inflammatory module, with *TLR4*, *MSR1*, *TIRAP*, and *CXCL8* emerging as candidate genes. Enrichment analyses linked these genes to innate immune and NF-κB-related pathways, whereas single-cell analyses localized the prioritized signals mainly to myeloid compartments during gout flares. Mendelian randomization suggested positive associations between genetically predicted expression of *TLR4*-axis genes and serum uric acid levels. Under electrostatic-guided docking conditions, fucoidan and alginate yielded plausible interaction models with *TLR4*, and normal mode and RMSF analyses suggested altered flexibility in the MD-2 region. In silico *Tlr4* knockout further perturbed urate-handling programs in renal proximal tubule-enriched cells. Together, these findings do not establish *TLR4* as a newly discovered hyperuricemia gene or confirm direct receptor antagonism by NSPs, but they provide an NSP-oriented integrative framework that prioritizes the *TLR4* axis, highlights myeloid-cell relevance, and nominates fucoidan and alginate for experimental follow-up.

## Simple Summary

Hyperuricemia is a common metabolic disorder that can lead to gout, kidney injury, and chronic inflammation. Natural non-starch polysaccharides from plants and algae have shown potential anti-inflammatory and metabolic benefits, but the molecular targets through which they may act in hyperuricemia are still unclear. In this study, we used an integrative computational and multi-omics strategy to identify biological targets linking these polysaccharides to hyperuricemia-associated inflammation. Our analyses highlighted the Toll-like receptor 4 (*TLR4*) pathway as a key candidate target and showed that this pathway is mainly active in myeloid immune cells during gout flares. Docking analyses further suggested that two representative polysaccharides, fucoidan and alginate, may plausibly interact with *TLR4*. Additional analyses also raised the possibility that *TLR4*-related signaling may be connected to renal transport systems involved in uric acid handling. Overall, this study prioritizes a potential inflammatory target and two candidate polysaccharides for future experimental studies of hyperuricemia.

## 1. Introduction

HUA is a common metabolic disorder characterized by elevated serum uric acid (UA) caused by excessive production or impaired excretion [1,2]. UA is the product of purine metabolism, synthesized mainly in the liver and excreted through the kidneys and intestines. Unlike most mammals, humans lack uricase, which contributes to relatively high circulating UA levels [3]. In healthy adults, serum UA generally does not exceed 420 μmol/L in men and 360 μmol/L in women [4]. Purines are derived from both dietary intake and endogenous metabolism [1,5]. In recent years, the global incidence and prevalence of HUA have increased markedly, thus rendering it a critical public health concern. From 2000 to 2023, the prevalence of hyperuricemia increased from 6.7% to 11.2% in women and from 12.3% to 18.6% in men, with prevalent cases rising from 126 million to 305 million in women and from 226 million to 500 million in men. This increase can be attributed to population growth and ageing, which account for approximately 50% of the observed rise [6]. Similar trends have also been reported in Asia, including China, and are associated with dietary transition, sedentary lifestyle, obesity, and metabolic syndrome [7].

Excess uric acid can promote monosodium urate crystal deposition and gout, and HUA is now increasingly regarded as an immunometabolic disorder linked to renal injury, metabolic disturbance, oxidative stress, endothelial dysfunction, tubular damage, and innate immune activation.

Conventional pharmacological interventions for hyperuricemia, such as xanthine oxidase inhibitors (e.g., allopurinol and febuxostat) and uricosuric agents, are effective in reducing serum uric acid levels. However, these interventions are frequently associated with adverse reactions, including hepatotoxicity, nephrotoxicity, gastrointestinal disturbances, and cardiovascular risks [8], which limit their long-term use. Consequently, the identification of safer and more biocompatible therapeutic alternatives has become a critical focus in the management of HUA. In this context, there has been an increased focus on natural bioactive substances, particularly NSPs, which are complex carbohydrates that are found in abundance in plants, algae, and microorganisms. NSPs have been shown to possess a variety of biological activities, including antioxidant, anti-inflammatory, immunomodulatory, and metabolic regulatory effects. This renders them promising candidates for the prevention and treatment of metabolic disorders [9]. Recent studies have indicated that certain NSPs have the capacity to alleviate hyperuricemia by modulating gut microbiota [10], inhibiting inflammatory pathways [11] and regulating uric acid transporters or related enzyme activities [12,13,14]. These findings suggest that NSPs may help reduce uric acid levels. However, the molecular targets that most plausibly connect NSP-associated signalling with HUA-related inflammation remain insufficiently defined; However, the molecular targets that most plausibly connect NSP-associated signalling with HUA-related inflammation remain insufficiently defined. Furthermore, the inflammatory pathways and cellular compartments most relevant to NSP action in HUA have not been systematically prioritised at the transcriptomic and systems-biology levels. The absence of target-level resolution imposes constraints on mechanistic interpretation and complicates the identification of representative NSPs that merit advancement for experimental validation.

Previous experimental and bioinformatics studies have already implicated *TLR4*-related inflammatory signaling in hyperuricemia and gout. Therefore, the novelty of the present study does not lie in proposing *TLR4* as an entirely new disease-associated gene. Instead, our aim was to establish an NSP-oriented integrative prioritization framework that could distinguish which inflammation-related targets are most consistently supported across complementary HUA-relevant datasets, identify the most relevant cellular context, and nominate representative NSP candidates for follow-up validation. To address this gap, the present study employed an integrative bioinformatics and computational strategy to prioritize candidate NSP-associated targets in hyperuricemia-associated inflammation. We first identified differentially expressed genes across HUA-relevant transcriptomic datasets to capture disease-associated inflammatory signals. Because not all DEGs are plausible mediators of NSP action, we then intersected these genes with a curated NSP-related receptor/target set to prioritize candidates that were not only dysregulated in HUA-relevant contexts but also mechanistically relevant to potential NSP sensing or signaling. We then used PPI analysis, GO and KEGG enrichment, and single-cell RNA sequencing to determine whether these candidates converged on coherent inflammatory pathways and cellular compartments. Mendelian randomization was subsequently applied to examine whether genetically predicted expression of prioritized genes was associated with serum uric acid levels. GutMGene-based orthogonal support analysis for *TLR4*. Finally, guided docking, structural dynamics analysis, exploratory ADMET profiling, and in silico *TLR4* knockout were used to evaluate representative NSP-target interaction models and to extend the analysis toward renal urate-handling programs. By integrating these complementary analyses, this study aimed to generate a more restrained, mechanistically organized framework for understanding how candidate NSPs may intersect with *TLR4*-centered inflammatory signaling in HUA. Because hyperuricemia-associated inflammation involves interconnected systemic immune, inflammatory, and renal components, we designed the study to identify convergent inflammatory signals across complementary HUA-relevant datasets rather than to build a single homogeneous transcriptomic cohort. Accordingly, datasets from different but biologically related contexts were used as independent evidence layers for target prioritization, with the aim of highlighting signals that recur across disease, treatment-response, immune-cell, and renal-associated settings.

## 2. Materials and Methods

### 2.1. Collection and Analysis of Differentially Expressed Genes

Transcriptomic datasets analyzed in this study were retrieved from the NCBI Gene Expression Omnibus (GEO) https://www.ncbi.nlm.nih.gov (accessed on 15 September 2025) under accession numbers GSE186871, GSE189228, GSE190205, GSE198133, GSE205963, GSE214587, GSE242872, GSE262687, and GSE300922 [15]. The corresponding GEO supplementary expression matrices were downloaded and used for downstream analysis. The nine transcriptomic datasets comprised a total of 90 samples, including GSE186871 (*n* = 9), GSE189228 (*n* = 6), GSE190205 (*n* = 9), GSE198133 (*n* = 12), GSE205963 (*n* = 6), GSE214587 (*n* = 18), GSE242872 (*n* = 12), GSE262687 (*n* = 6), and GSE300922 (*n* = 12). These files included both count-based matrices and preprocessed gene-level expression matrices, thereby allowing integrated screening across human blood, human cell, and murine inflammatory models. The included datasets were not selected to represent one identical biological compartment, but rather to capture complementary layers of HUA-relevant biology, including systemic immune inflammation, gout-associated PBMC responses, inflammatory perturbation models, treatment-responsive signals, and renal-associated processes. On this basis, the integration strategy was designed to identify genes and pathways that recur across related but non-identical biological contexts, thereby supporting target prioritization at the systems level. 17 comparisons were used to capture convergent inflammatory signals across complementary HUA-relevant contexts rather than to define one uniform transcriptomic state. For GSE198133 and GSE189228, raw count matrices were analyzed together with the corresponding NCBI gene annotation resources, including Human.GRCh38.p13.annot.tsv and Human.GRCh38.p14.annot.tsv, to ensure compatibility with the annotation versions associated with each dataset. For the remaining datasets, the downloaded matrices were already provided at the gene level and were analyzed directly after format inspection and sample-group parsing.

All bioinformatic analyses were performed in RStudio (version 4.4.3), with the DESeq2 (version 1.44.0), dplyr (version 1.1.4) [16], ggplot2 (version 4.0.0), limma (version 3.62.2), readxl (version 1.4.5), and biomaRt (version 2.62.1) packages. Because the included datasets differed in species, tissue source, disease context, and experimental design, raw expression matrices were not directly merged for joint modeling. Instead, each dataset was analyzed independently within its own predefined comparison, and cross-dataset integration was performed only after within-dataset differential expression analysis. Sample metadata tables were manually constructed according to the original GEO group annotations to define experimental contrasts. In total, 17 comparison groups were analyzed: GSE186871 Model vs. Control, GSE186871 Treatment vs. Model, GSE189228 HUA vs. Control, GSE190205 Model vs. Control, GSE190205 PEC vs. Model, GSE198133 UA vs. Control, GSE198133 Sunflower vs. UA, GSE198133 Abietic acid vs. UA, GSE205963 Tet2KO MSU vs. WT MSU, GSE214587 WT MSU vs. WT none, GSE214587 WT Api MSU vs. WT MSU, GSE214587 CD38KO MSU vs. WT MSU, GSE242872 MSU8h vs. Con8h, GSE242872 MSU24h vs. Con24h, GSE262687 Model vs. Control, GSE300922 HUA vs. Control, and GSE300922 XZSW vs. HUA.

For count-based or read-count matrices, raw counts were imported into R and analyzed using DESeq2 [17] using a DESeqDataSet with the design formula~group. Genes with counts ≥ 10 in at least the size of the smallest experimental group were retained for modeling, and standard median-of-ratios normalization was applied [18,19]. A DESeqDataSet object was generated with the design formula~group, followed by normalization and statistical modeling using the DESeq() function. DESeq2 employs a negative binomial generalized linear model to account for over-dispersion in RNA-seq count data, with internal normalization using the median-of-ratios method to correct for library size differences and RNA composition bias [17]. For preprocessed FPKM or gene-level expression matrices, differential expression analysis was conducted using the limma framework. Expression matrices were checked for feature format and sample naming consistency, transformed where necessary, and analyzed with a linear model followed by empirical Bayes moderation [20,21]. For all datasets, genes with an adjusted *p*-value (Benjamini–Hochberg corrected) < 0.05 and an absolute log2 fold change ≥ 1 were considered significantly differentially expressed (DEGs).

Potential batch effects across studies were primarily controlled through study-specific differential analysis rather than direct cross-study matrix correction. Each contrast was therefore evaluated only within its own dataset. Because downstream integration was based on significant DEG sets, receptor overlap, and pathway-level convergence rather than pooled continuous expression values, the influence of between-study platform and processing differences was reduced, although residual heterogeneity could not be fully eliminated.

After differential expression analysis, comparison-specific DEG tables were exported as .csv files for reproducibility and downstream integration. For GSE198133 and GSE189228, DEG lists were cross-referenced with the corresponding NCBI annotation files, and standardized gene symbols were retrieved from Ensembl (version 115) using biomaRt (version 2.62.1) [22]. For datasets already provided with gene-level identifiers, reported gene symbols were used after consistency checking. The annotated results were then compiled into unified tables containing complete DEG outputs, significant DEG outputs, and receptor-overlap outputs for each comparison.

To prioritize candidate molecular targets potentially relevant to NSP activity, all significant DEG sets were further integrated across comparisons. Unique DEGs from all transcriptomic contrasts were pooled to construct a nonredundant DEG background, and this integrated gene set was intersected with a curated NSP-related receptor file, receptor_gene_mapping_human_fixed4.12.csv. This overlap step was added to narrow down from a wide set of disease-related DEGs to a smaller group of biologically likely NSP-responsive candidates. Specifically, differential expression analysis first identified genes associated with HUA-relevant inflammatory states, whereas receptor overlap was subsequently used to determine which of these dysregulated genes were also mechanistically plausible as NSP-related host recognition or signaling candidates. This procedure generated both comparison-specific receptor-overlap tables and a global nonredundant overlap set for subsequent visualization, pathway enrichment, and candidate prioritization. This integrative workflow reduced a broad transcriptomic search space to a compact group of biologically plausible receptor-associated candidates for downstream functional interpretation.

### 2.2. Screening of Candidate Gene Targets

After constructing the integrated nonredundant DEG set from the transcriptomic comparisons, we next sought to determine which of these dysregulated genes were most plausibly relevant to NSP action. To achieve this, we compiled NSP-associated receptors from PRRDB 2.0 https://webs.iiitd.edu.in/raghava/prrdb2/index.html (accessed on 15 September 2025) [23] and used them as a biologically informed filter for downstream candidate prioritization. Receptor selection was based on ligand annotation in PRRDB 2.0, and only receptor entries associated with non-starch polysaccharides were retained for downstream analysis. Gene annotation was then standardized in RStudio (version 4.4.3) using the AnnotationDbi package (version 1.68.0) to obtain official gene symbols and Ensembl gene IDs (ENSGIDs). To improve database consistency, duplicated entries, ambiguous identifiers, and unmapped records were removed after symbol harmonization, and the resulting curated receptor list was checked for compatibility with the transcriptomic gene-annotation framework used in Section 2.1. This curation step served as an annotation-level validation procedure to ensure that the receptor entries used for overlap analysis were nonredundant, mappable, and compatible with the DEG integration workflow. Because PRRDB 2.0 is a curated receptor resource derived from published evidence, its use also provided literature-supported biological grounding for ligand-based receptor filtering. Comparison-specific overlaps were retained for traceability, and the global receptor-overlap set was used for Venn visualization and downstream candidate prioritization. Genes present in both the curated receptor list and the integrated DEG pool were defined as candidate NSP-related targets, and the final overlap table was saved as DE_receptor_matched.csv. Importantly, this downstream integration was based on comparison-specific DEG overlap and the global nonredundant overlap set and was therefore implemented as a target-prioritization strategy rather than as a pooled transcriptomic meta-analysis. In other words, candidate genes were prioritized according to recurrent support across independent HUA-relevant comparisons, rather than by direct joint modeling of all transcriptomic samples. Thus, the final 19 genes were not selected by arbitrary ranking but were defined as the complete overlap between the integrated nonredundant significant DEG set and the curated NSP-related receptor set.

### 2.3. Construction of the Protein–Protein Interaction (PPI) Network

After defining the NSP-related candidate target set, we next examined whether these genes formed a coherent interaction module at the protein level. Candidate target genes were submitted to the STRING 12.0 database https://www.string-db.org (accessed on 17 November 2025) to construct a PPI network [24]. The analysis was restricted to Homo sapiens, with a minimum interaction confidence score of 0.7, which corresponds to the high-confidence category in the STRING database and was selected to improve network specificity for candidate prioritization, although this conservative threshold may exclude some lower-confidence but potentially relevant interactions. Resulting interaction data were exported in .tsv format and imported into Cytoscape 3.10.4 for visualization and topological analysis [25]. In addition to node connectivity and interaction structure, standard network topological metrics were assessed to characterize the resulting high-confidence PPI network, including the average clustering coefficient, network density, and average shortest path length. The average shortest path length was calculated on the full network when the graph was connected. The PPI network revealed several closely associated proteins—including *IL1A*, *IL1B*, *MYD88*, *TRAF6*, *IRAK1*, *IRAK2*, *IRAK4*, and *TIRAP*—suggesting functional convergence and signaling pathway relevance among these molecules.

### 2.4. GO and KEGG Pathway Enrichment Analysis

To further interpret the biological functions represented by the candidate target module and its key interactors, we performed Gene Ontology (GO) and KEGG pathway enrichment analyses using the DAVID database (version 2025_2) [26]. Official gene symbols and Homo sapiens were specified as the identifier and organism, respectively, with the whole human genome as background. Enrichment results were exported as BP.txt, CC.txt, MF.txt, and KEGG.txt, and further processed and visualized in RStudio (version 4.4.3). The top eight significantly enriched pathways were visualized using bubble plots and chord diagrams to illustrate key functional pathways and gene-pathway associations.

### 2.5. Single-Cell RNA Sequencing Analysis

Because pathway-level enrichment alone could not resolve the cellular context of the prioritized inflammatory signals, we next used single-cell RNA sequencing to localize these signals across immune cell populations. Single-cell RNA sequencing data from dataset GSE211783, encompassing peripheral blood mononuclear cells (PBMCs) from three patients during acute gout flares and their subsequent remission phase, were acquired from the Gene Expression Omnibus (GEO) database https://www.ncbi.nlm.nih.gov (accessed on 20 November 2025) [15]. All analyses were conducted using Seurat v5.0.1 in the R environment (version 4.4.3) [27]. Following the creation and merging of individual sample objects, stringent quality control was applied to remove low-quality cells. Cells with fewer than 200 or more than 6000 detected genes, fewer than 500 or more than 50,000 unique molecular identifiers (UMIs), or more than 10% mitochondrial gene content were excluded. The data were then normalized using the LogNormalize method (scale.factor = 10,000), and the top 2000 most variable genes were identified using the vst method for downstream analysis. Subsequent steps included data scaling, principal component analysis (PCA) on the top 30 principal components, and graph-based clustering using the Louvain algorithm at resolution 0.8. Cell populations were visualized in two dimensions using uniform manifold approximation and projection (UMAP). Automated cell type annotation was performed with the SingleR tool (version 2.0 or higher) using the MonacoImmuneData reference dataset from celldex https://www.ncbi.nlm.nih.gov/geo/query/acc.cgi?acc=GSE107011 (accessed on 20 November 2025) [28]. Differential gene expression between the Flare and Remission groups was assessed across all cells and within specific clusters using the Wilcoxon rank-sum test (via FindMarkers with min.pct = 0.1, logfc.threshold = 0.25), with *p*-values adjusted by the Benjamini–Hochberg method. To pinpoint cell-type-specific responses, a focused analysis was subsequently conducted on clusters enriched for monocytes. All visualization plots were generated using standard Seurat functions.

### 2.6. Mendelian Randomization Analysis

To complement the transcriptomic and single-cell prioritization results with genetic evidence, we next conducted a two-sample Mendelian randomization (MR) analysis to test whether genetically predicted expression of selected candidate genes was associated with serum uric acid (SUA) levels [29], following STROBE-MR reporting guidance [30]. Based on the upstream prioritization analyses, *TLR4*, *CXCL8*, *TIRAP*, and *MSR1* were selected for MR testing.

Genetic instruments for gene expression were defined as expression quantitative trait loci (eQTLs) obtained from the OpenGWAS database https://opengwas.io (accessed on 22 November 2025) (primarily cis-eQTLs from blood or other available relevant tissues). Summary statistics for SUA were primarily taken from the large-scale European-ancestry dataset ebi-a-GCST90018977, which served as the main outcome for the manuscript figures; additional SUA-related datasets were screened during the broader exploratory phase. For each gene-outcome pair, we harmonized the exposure and outcome datasets by aligning effect alleles, beta coefficients, standard errors (SE), and effect allele frequencies (EAF) [29].

We implemented a rigorous multi-step quality control pipeline to ensure instrument validity. We further removed variants with low effect allele frequency (EAF < 0.01) and excluded palindromic SNPs with ambiguous strand information during harmonization using the TwoSampleMR package (version 0.5.6 or higher). We also applied the Steiger directionality test to confirm that the genetic instruments primarily influenced the exposure (gene expression) rather than the outcome directly. To mitigate weak instrument bias, we calculated the F-statistic for each SNP and for the overall instrument set, excluding any with F < 10. Finally, we used PhenoScanner V2 and LDtrait tools (version 7.1.0) to systematically screen and remove SNPs known to be associated with potential confounders (e.g., BMI, alcohol intake, or other metabolic traits) or directly with the outcome at genome-wide significance [31,32].

The inverse-variance weighted (IVW) method with multiplicative random effects served as the primary analysis for estimating causal effects. To assess the robustness of the findings, we performed several complementary sensitivity analyses. MR-Egger regression with bootstrapped standard errors was used to evaluate potential directional pleiotropy via the intercept test, and Cochran’s Q statistic was used to quantify heterogeneity; a random-effects IVW model was adopted when significant heterogeneity was detected (P_Q < 0.05). In addition, weighted median and weighted mode methods were used as pleiotropy-robust alternatives, and MR-PRESSO was applied to detect and correct outlier variants contributing to horizontal pleiotropy [33]. Radial MR was used to identify and remove disproportionate outliers. All analyses were performed in R (version 4.4.3) using the TwoSampleMR and MR-PRESSO packages, with statistical significance determined after appropriate multiple-testing correction where applicable.

### 2.7. GutMGene-Based Orthogonal Support Analysis for TLR4

As an additional orthogonal extension beyond host-side transcriptomic and genetic evidence, we queried GutMGene https://bio-computing.hrbmu.edu.cn/gutmgene/#/home (accessed on 18 June 2026) to explore whether the prioritized *TLR4* signal was also supported within a gut microbe-metabolite-host interaction context. To obtain gut-derived supplementary support for the prioritized host target, we queried the human modules of GutMGene https://bio-computing.hrbmu.edu.cn/gutmgene/#/home (accessed 18 June 2026), which curate experimentally supported gut microbe-metabolite, gut microbe-host gene, and metabolite-host gene associations [34,35]. The downloadable “Host Gene” and “Microbial Metabolite” tables were imported into R (version 4.4.3) for analysis. Entries containing *TLR4* were first extracted from the host-gene dataset and then matched to the core gut microbes carried forward from our upstream workflow. Analyses were restricted to human associations. Duplicate microbe–metabolite–*TLR4* records were collapsed at the triplet level, while the reported alteration direction was retained as activation, inhibition, or none. A tripartite network was then constructed to visualize the gut microbe–metabolite–*TLR4* relationships, and a bubble support map was generated to summarize the direction and cumulative evidence intensity for each microbe-metabolite pair converging on *TLR4*. Microbial metabolites were descriptively prioritized according to their recurrence across GutMGene records, consistency of reported regulatory direction, and convergence with the core gut microbes retained from the upstream workflow. No formal statistical weighting model was applied; instead, evidence strength was summarized qualitatively based on repeated curated associations linking a given microbe-metabolite pair to *TLR4*. Because this analysis was intended as an external literature-based extension of the host-side *TLR4* result, GutMGene findings were interpreted as supplementary support for a potential gut-derived upstream context rather than as evidence redefining the renal cellular localization of *TLR4*.

### 2.8. Molecular Docking

After prioritizing *TLR4* and related targets through transcriptomic, cellular, genetic, and database-supported analyses, we next explored whether representative NSPs might plausibly interact with these proteins at the structural level. To further explore plausible interactions between representative NSPs (fucoidan and alginate) and prioritized protein targets, molecular docking simulations were performed for *TLR4* and *CXCL8*. No experimentally determined three-dimensional structure was available for *MSR1*, and *TIRAP*, as a cytoplasmic adaptor protein, was not considered an appropriate extracellular docking target for large polysaccharides.

#### 2.8.1. Ligand Preparation

The hexasaccharide repeating units of fucoidan and alginate were constructed using the GLYCAM-Web Carbohydrate Builder tool, https://glycam.org (accessed on 16 April 2026) [36]. The constructed ligands were directly exported from GLYCAM-Web in PDB format.

#### 2.8.2. Protein Preparation

The 3D structures of *TLR4* (PDB ID: 3FXI) and *CXCL8* (PDB ID: 1IL8) were retrieved from the RCSB Protein Data Bank https://www.rcsb.org/ (accessed on 16 April 2026). For *TLR4*, the biologically relevant complex including the MD-2 subunit was retained. The protein structures were prepared using UCSF ChimeraX (version 1.11.1). First, all water molecules, ions, and other heteroatoms were removed, except for the co-crystallized ligand retained temporarily for reference. Missing side chains were then added using Modeller (version 10.8) through the ChimeraX modeller command. Finally, the added residues were energy-minimized with 200 steps of steepest descent. All prepared proteins were saved in PDBQT format.

#### 2.8.3. Docking Protocol

Docking was performed using CB-Dock2 (version 2.0), which employs curvature-based cavity detection and AutoDock Vina (version 1.2.7) for scoring [37].

Round 1 (Blind docking): fucoidan and alginate hexasaccharides were docked independently against the fully prepared structures of *TLR4* and *CXCL8*. The docking box was set to cover the entire protein surface.

Round 2 (Cavity-guided docking): The top three binding cavities predicted by CB-Dock2 for each protein were used as docking targets. For *TLR4*, the known LPS-binding pocket on MD-2 was specifically included. The docking box was centered on each cavity with dimensions automatically determined by the algorithm.

Round 3 (Electrostatic-guided docking): To test the hypothesis of electrostatic interactions, positively charged residues (Arg, Lys) on the surface of each protein were identified using the “charge” tool in ChimeraX. A list of these residues is provided in Supplementary Tables S6 and S7. In CB-Dock2, these positively charged residues were selected to guide the molecular docking of fucoidan and alginate hexasaccharides. These guided docking settings were used to explore electrostatically plausible binding poses rather than to establish direct binding specificity under physiological conditions.

For every docking run, the best binding pose was selected based on the lowest binding energy (kcal/mol). For each ligand–target pair, the top 5 poses were retained.

#### 2.8.4. Post-Docking Analysis and Validation

All docking results were exported as PDB files and visualized using UCSF ChimeraX (version 1.11.1) to inspect the binding poses, interface orientation, and spatial arrangement of the polysaccharide hexasaccharide units within the target protein cavities. Key intermolecular interactions were subsequently analyzed using the Protein-Ligand Interaction Profiler (PLIP, version 2.4) [38]. For each top-ranked docking pose, the corresponding protein–ligand complex PDB file was uploaded to the PLIP web server https://plip-tool.biotec.tu-dresden.de/plip-web/plip/index (accessed on 16 April 2026). PLIP identified and reported seven interaction types according to default geometric criteria: hydrogen bonds, salt bridges, hydrophobic contacts, π-stacking, π-cation interactions, water bridges, and halogen bonds. Emphasis was placed on electrostatic interactions, including salt bridges and hydrogen bonds formed between the negatively charged sulfate groups (fucoidan) or carboxyl groups (alginate) and the positively charged residues (Arg and Lys) on the *TLR4* surface. Interaction patterns were exported as CSV files, along with publication-ready schematic diagrams and PyMOL (version 3.1.6.1) session files.

### 2.9. Normal Mode Analysis to Evaluate the Structural Dynamics of TLR4 upon Polysaccharide Binding

Because docking provides static interaction models only, we further evaluated whether the NSP-associated *TLR4* poses were accompanied by changes in receptor flexibility and structural dynamics. To investigate the effects of fucoidan and alginate binding on the structural stability and flexibility of the *TLR4* receptor, this study employed the iMODS server http://imods.chaconlab.org/ (accessed on 17 April 2026), an internal coordinate normal mode analysis tool for normal mode analysis (NMA) [39]. The apo–*TLR4* structure (PDB ID: 3FXI, corresponding to the 3FXI group), the alginate–*TLR4* complex PDB file, and the fucoidan–*TLR4* complex PDB file were uploaded separately. The default elastic network model (Elastic Network Model, Sigmoid cutoff) was used to compute the first 20 lowest-frequency normal modes. The analyzed parameters included main-chain deformability (Deformability), B-factor/Mobility, eigenvalues (Eigenvalues), variance contribution (Variance), covariance matrix (Covariance matrix), and elastic network diagram.

Root mean square fluctuation (RMSF) analysis was performed on the fucoidan–*TLR4* complex, alginate–*TLR4* complex, apo–*TLR4* monomer, and a neutral glucose-hexasaccharide control using the CABS-flex 2.0 server https://biocomp.chem.uw.edu.pl/CABSflex2/ (accessed on 17 April 2026) [40]. The resulting RMSF values for chain D (MD-2) residues 19–158 were extracted from the output files. These numerical data were imported into RStudio (version 4.4.3, consistent with previous analyses) using ggplot2 and dplyr. After filtering and sorting by residue position, the RMSF curves were plotted to compare flexibility changes in the MD-2 pocket region across all four models.

In addition, to examine whether charge rather than saccharide size alone might contribute to the observed flexibility changes, we generated a neutral α-1,4-linked glucose hexasaccharide with GLYCAM-Web https://glycam.org (accessed on 17 April 2026), docked it with CB-Dock2 http://cao.labshare.cn:10380/cb-dock2/php/manual.php (accessed on 17 April 2026), and submitted the resulting model to CABS-flex 2.0 for RMSF analysis. The resulting .csv files were analyzed in RStudio using the same workflow applied to the fucoidan- and alginate-associated models.

### 2.10. Exploratory ADMET Profiling of Non-Starch Polysaccharides

To explore the predicted pharmacokinetic and toxicity characteristics of the candidate NSPs (fucoidan and alginate), we performed ADMET profiling using the pkCSM online tool https://biosig.lab.uq.edu.au/pkcsm/ (accessed on 20 April 2026) [41]. Input molecules were converted from PDB to SMILES format using the Online SMILES Translator and Structure File Generator https://cactus.nci.nih.gov/translate/ (accessed on 20 April 2026). A total of 30 indicators spanning absorption, distribution, metabolism, excretion, and toxicity were generated based on the graph-based signature model, and the results were summarized in tabular form [42].

### 2.11. Virtual Knockout of TLR4 and Downstream Functional Enrichment Analysis

To extend the analysis from candidate binding plausibility to systems-level functional consequences, we performed an in silico *Tlr4* knockout analysis in a kidney single-cell context. The single-cell RNA-seq data used for virtual knockout analysis were derived from the previously processed mouse kidney dataset GSE256431. Based on unsupervised clustering and marker-guided annotation performed in Seurat v5, proximal tubule-enriched cells were extracted for downstream analysis [28]. After layer joining and log-normalization, the normalized expression matrix from the RNA assay was used as the input for virtual perturbation analysis. To ensure computational feasibility while preserving transcriptome-wide coverage, up to 2000 proximal tubular cells were randomly sampled for each run.

Virtual knockout of *Tlr4* was performed using scTenifoldKnk (version 1.0.3), a single-cell network perturbation framework that infers gene knockout-responsive regulatory changes through construction of wild-type gene regulatory networks, in silico deletion of the target gene, and manifold alignment-based comparison of the perturbed and unperturbed networks [43]. Because the number of detected genes exceeded the memory limit of the local computing environment, the full gene set was divided into sequential chunks of 2000 genes, and *Tlr4* was forcibly included in each chunk to ensure consistent target-gene representation. For each chunk, scTenifoldKnk was run with the following parameters: qc = FALSE, nc_nNet = 15, nc_nCells = 500, nc_nComp = 3, and nCores = 2. The differential regulation outputs from all chunks were subsequently merged, and for genes appearing in multiple chunks, the record with the largest absolute Z score was retained as the representative result. Genes with FDR < 0.05 were considered significantly perturbed.

For visualization, significantly perturbed downstream genes were grouped into broad functional categories, including transport/tubular handling, metabolism/detoxification, tubular markers/function, and stress/inflammation, according to their annotated biological roles. A focused urate-transport panel was additionally constructed to highlight renal transporters implicated in urate reabsorption, secretion/excretion, and associated proximal tubular transport processes.

To further characterize the biological programs represented by the significantly perturbed genes, over-representation analysis (ORA) was performed using the enricher function in clusterProfiler (4.14.6) [44]. Mouse-native GO Biological Process gene sets were obtained from the Molecular Signatures Database (MSigDB, version 2026.1.Hs) via msigdbr (version 2026.1.0), using the C5 GO:BP collection [45]. Enriched terms were ranked by adjusted *p* value, and the top terms were visualized as a dot plot in which dot size represented gene count, the x-axis represented gene ratio, and dot color represented −log10(FDR).

### 2.12. Use of Artificial Intelligence

During the preparation of this work, the authors used LLMs (ChatGPT version 5.4) to assist with language polishing, information organization and graphical abstract representaion. After using these tools, the authors reviewed and edited the content as needed and remain fully responsible for the content of the publication.

## 3. Results

### 3.1. Prioritization of Hyperuricemia-Associated Candidate NSP Targets

Integrative screening across 17 transcriptomic contrasts identified a compact NSP-receptor-overlapping candidate set enriched for innate immune genes. After pooling significant DEGs from human blood, human cell, and murine inflammatory models, we intersected the nonredundant DEG background with a curated NSP-related receptor set to prioritize candidate targets for downstream analysis.

**Figure 1:**
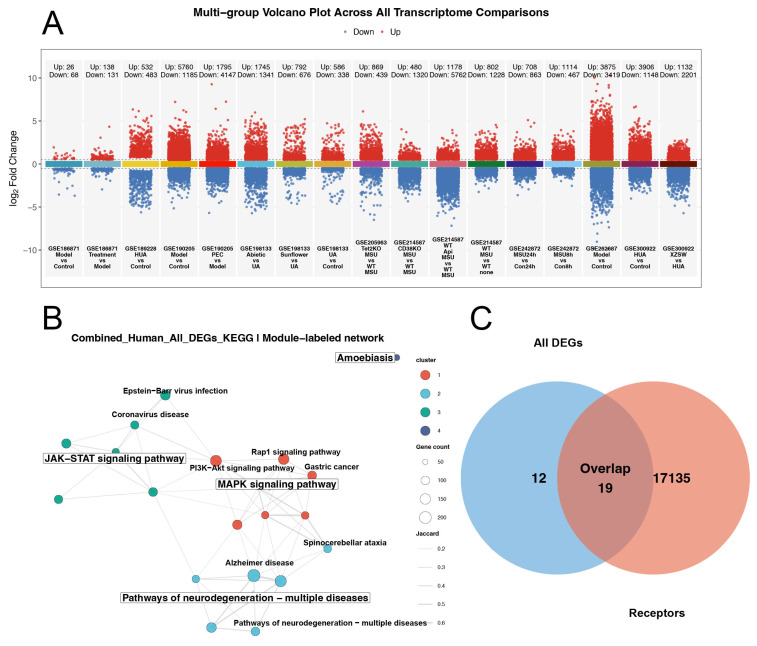
Integrative transcriptomic prioritization of NSP-related candidate genes in hyperuricemia. (**A**) Multi-group plot of significant DEGs across 17 transcriptome comparisons, showing only genes that passed the significance thresholds (red, up-regulated; blue, down-regulated; The different colors on the X-axis represent different samples; threshold |log2FC| ≥ 1 and adjusted *p* < 0.05). The numbers of up- and down-regulated genes were 26 and 68 for GSE186871 Model vs. Control, 138 and 131 for GSE186871 Treatment vs. Model, 532 and 483 for GSE189228 HUA vs. Control, 5760 and 1185 for GSE190205 Model vs. Control, 1795 and 4147 for GSE190205 PEC vs. Model, 1745 and 1341 for GSE198133 Abietic acid vs. UA, 792 and 676 for GSE198133 Sunflower vs. UA, 586 and 338 for GSE198133 UA vs. Control, 869 and 439 for GSE205963 Tet2KO MSU vs. WT MSU, 480 and 1320 for GSE214587 CD38KO MSU vs. WT MSU, 1178 and 5762 for GSE214587 WT Api MSU vs. WT MSU, 802 and 1228 for GSE214587 WT MSU vs. WT none, 708 and 863 for GSE242872 MSU24h vs. Con24h, 1114 and 467 for GSE242872 MSU8h vs. Con8h, 3875 and 3419 for GSE262687 Model vs. Control, 3906 and 1148 for GSE300922 HUA vs. Control, and 1132 and 2201 for GSE300922 XZSW vs. HUA. (**B**) Module-labeled KEGG enrichment network derived from the combined human DEG set, showing interconnected enriched pathways grouped into major signaling modules. Representative pathway nodes, including MAPK signaling, JAK-STAT signaling, and neurodegeneration-related pathways, are labeled to highlight the main biological themes of the network. (**C**) Venn analysis of all nonredundant DEGs and the curated NSP-related receptor set identified 19 overlapping genes between 17,135 unique DEGs and 12 receptor-associated genes. In the Venn diagram, the blue circle represents the receptor set, the salmon circle represents all nonredundant DEGs, and the overlapping shaded region represents the shared genes.

The multi-group volcano summary showed substantial heterogeneity in DEG burden across datasets (Figure 1A), ranging from 94 genes in GSE186871 Model vs. Control to 7294 genes in GSE262687 Model vs. Control, while the clinically derived GSE189228 HUA vs. Control comparison yielded 1015 DEGs. Together, these contrasts captured both disease-associated and intervention-associated transcriptional signals rather than a single context-specific DEG pattern [46].

KEGG module-network analysis of the combined human DEG set further indicated that these transcriptomic changes converged on interconnected signaling hubs represented by PI3K-Akt signaling, MAPK signaling, and neurodegeneration-related modules, supporting a structured and biologically coherent disease-associated network rather than isolated DEG accumulation (Figure 1B).

Venn integration with the curated NSP receptor set identified 19 unique candidate genes overlapping the NSP-related receptor set and the integrated DEG pool (Figure 1C). Specifically, 17,154 nonredundant DEGs intersected with 31 curated receptor-associated genes to yield 19 shared candidates, namely *LGALS4*, *MRC1*, *SCARA3*, *MRC2*, *LGALS1*, *TLR2*, *CLEC7A*, *TLR1*, *MSR1*, *TIRAP*, *TLR9*, *NOD1*, *LGALS9*, *NLRP3*, *LGALS3*, *NOD2*, *TLR4*, *LGALS8*, and *LGALS12*. Notably, the overlapping genes included inflammatory regulators such as *TLR4* and *MSR1*, supporting the view that the most reproducible overlap was concentrated in innate immune signaling rather than in broad metabolic pathways [47,48].

Overall, this prioritization step reduced 17,154 unique DEGs to a compact 19-gene receptor-overlapping candidate set and established an initial rationale for focusing subsequent analyses on a *TLR4*-centered inflammatory module potentially relevant to NSP action [49].

### 3.2. A TLR4-Centered Protein–Protein Interaction Module Emerges from Candidate Target Analysis

**Figure 2:**
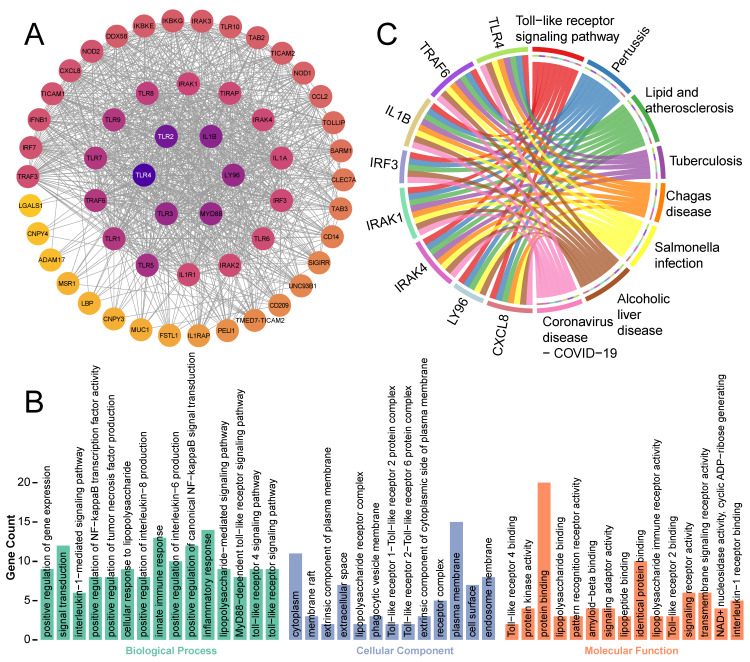
Protein interaction and functional enrichment analyses of prioritized NSP-related candidate genes. (**A**) STRING-derived PPI network of the candidate set and closely connected interactors (Homo sapiens; minimum interaction score = 0.7). Node size and color indicate degree centrality, highlighting *TLR4*-centered connectivity within the network. (**B**) Gene Ontology enrichment of the key network genes across the BP, CC, and MF categories; the top terms ranked by gene count are shown after Benjamini–Hochberg correction. (**C**) KEGG chord diagram showing the relationships between core genes and the top eight significantly enriched pathways.

Based on the prioritized candidate genes, we constructed a protein–protein interaction (PPI) network using STRING to examine whether these genes converged on a coherent interaction module (Figure 2A) [24]. The resulting expanded network comprised 54 nodes and 451 edges, indicating extensive functional connectivity among the candidate genes and their closely connected interactors. Topological analysis showed an average degree of 17.346, an average clustering coefficient of 0.737, and a network density of 0.340, consistent with a relatively dense and highly interconnected interaction structure. Because two nodes were disconnected after confidence filtering, the average shortest path length was evaluated on the largest connected component, which contained 52 nodes and 451 edges, and yielded a value of 1.705. Together, these properties support the view that the prioritized genes converged on a compact interaction module.

Network analysis showed that *TLR4* served as a central node, closely interacting with MYD88, IL1B, TLR2, and others to form a core module. Other nodes, such as *TIRAP*, *TIRAP*, IRAK1, and *CXCL8*, exhibited high connectivity, indicating coordinated regulation through downstream signaling cascades (e.g., the TLR-MYD88-IRAK pathway).

By calculating node degree and betweenness centrality, we identified 11 key hub genes: *TLR4*, TLR2, *TIRAP*, *MSR1*, *CCL2* (C-C motif chemokine ligand 2), *CXCL8*, *MUC1*, *IL1B, MYD88*, *TIRAP*, and *IRAK1*. These genes occupied central positions in the network, with *TLR4* acting as an upstream receptor directly interacting with *MYD88* and *IRAK1* to activate NF-κB-related signaling and the expression of pro-inflammatory factors such as *IL1B* and *CXCL8* [50]. Together, the network architecture positioned *TLR4* as an upstream organizing node and provided the basis for subsequent pathway-level analyses [51].

### 3.3. Functional Enrichment Analyses Highlight TLR4-Associated Innate Immune Signaling

Based on the key genes identified from the PPI network, we performed GO and KEGG enrichment analyses to further define their biological functions and pathway associations [52].

The GO analysis (Figure 2B) revealed that in the Biological Process (BP) category, the key genes were predominantly enriched in pathways associated with innate immunity and inflammatory activation. These included toll-like receptor–related pathways—such as the toll-like receptor signaling pathway, toll-like receptor 4 signaling pathway, and MyD88-dependent toll-like receptor signaling pathway—as well as downstream inflammatory responses, including the inflammatory response, positive regulation of NF-κB transcription factor activity, interleukin-1–mediated signaling pathway, and lipopolysaccharide-mediated signaling pathway. Altogether, these terms point toward a coherent biological theme centered on pathogen recognition via pattern recognition receptors and the initiation of pro-inflammatory signaling cascades leading to cytokine production.

The KEGG pathway analysis (Figure 2C) identified 36 significantly enriched pathways, among which the top-ranked pathways were dominated by immune and inflammatory processes [53]. These included the Toll-like receptor signaling pathway, NF-κB signaling pathway, NOD-like receptor signaling pathway, IL-17 signaling pathway, and MAPK signaling pathway, as well as infection-associated pathways such as Coronavirus disease-COVID-19, Tuberculosis, and Salmonella infection. Many of these pathways converge on classical innate immune signaling modules, consistent with the GO results.

To further illustrate gene–pathway relationships, a chord diagram of the top eight enriched pathways was constructed (Figure 2C). Core genes such as *TLR4*, *MYD88*, *IRAK1*, and *IL1B* were mapped to multiple pathways, particularly the Toll-like receptor and NF-κB signaling pathways, highlighting their central roles in coordinating early immune recognition and inflammatory amplification [48].

Complete GO and KEGG enrichment results are provided in the Supplementary Materials, including all significant terms/pathways together with adjusted *p*-values, enrichment scores, and gene counts for each entry (Biological Process, Supplementary Table S1; Cellular Component, Table S2; Molecular Function, Table S3 and KEGG, Table S4).

Collectively, these enrichment results indicate that the prioritized candidate set is concentrated in innate immune and inflammatory signaling pathways, reinforcing the rationale for focusing downstream analyses on the *TLR4* axis rather than on a diffuse set of unrelated genes.

### 3.4. Single-Cell Analyses Place Core TLR4-Axis Signals Predominantly in Monocyte and Neutrophil Clusters

Single-cell RNA sequencing analysis of GSE211783 PBMCs from patients sampled during acute gout flares and remission was used to localize the prioritized genes across immune cell populations [15]. After quality control, clustering, and annotation, major immune compartments including T cells, monocytes, neutrophils, NK cells, B cells, and several minor myeloid populations were resolved for downstream expression mapping, and UMAP was used for visualization [54].

**Figure 3:**
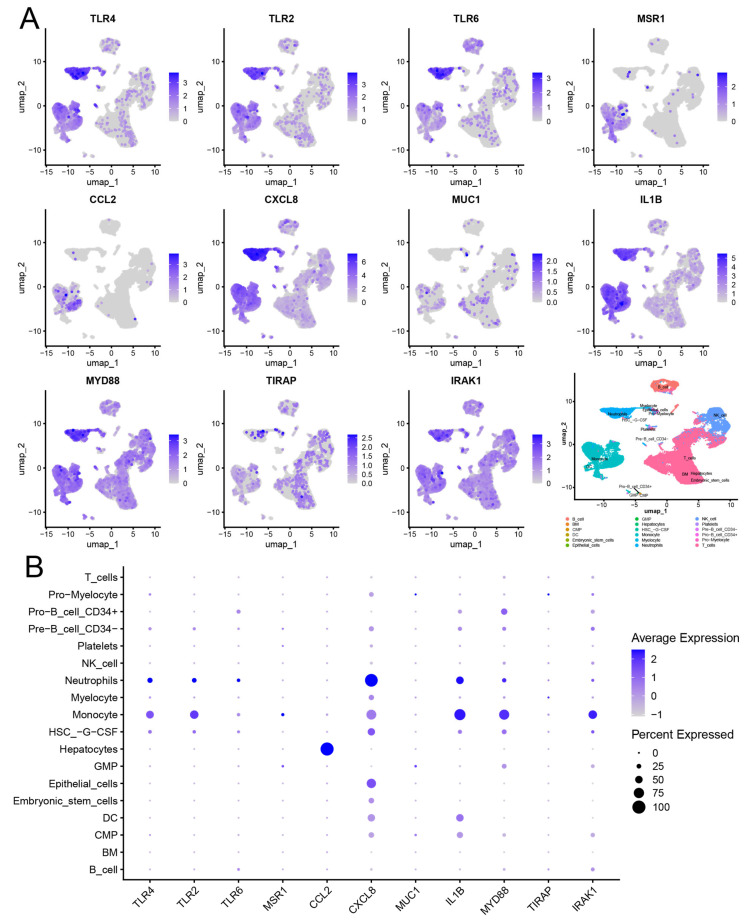
Single-cell transcriptomic landscape of gout PBMCs and expression of key NSP-target genes. (**A**) UMAP visualization of PBMC single-cell RNA-seq data (GSE211783), showing the major annotated immune cell populations. And feature plots showing that the prioritized genes are expressed predominantly in monocyte and neutrophil clusters. (**B**) Dot plot of selected TLR signaling pathway-related genes across annotated cell types. Dot color indicates average expression level, and dot size indicates the percentage of cells expressing each gene.

UMAP visualization resolved major immune populations, including T cells, monocytes, neutrophils, NK cells, B cells, and smaller myeloid subsets (Figure 3A). Cells from flare and remission samples were broadly intermingled on the UMAP, indicating that clustering was driven mainly by cell-type identity rather than by global condition-specific separation.

A cell-type dot plot of selected genes (*TIRAP*, *IL1B*, *TLR2*, *MYD88*, *IRAK1*, *IRAK4*, *TLR4*, *TIRAP*, *CXCL8*, *CCL2*, *MUC1*, and *MSR1*) showed that the prioritized *TLR4*-axis genes were preferentially expressed in myeloid compartments, particularly monocytes and neutrophils (Figure 3B). *TLR4*, *MYD88*, *TIRAP*, *IRAK1*, *MSR1*, *CXCL8*, and *IL1B* displayed stronger expression signals in these cell types than in lymphoid populations, whereas expression in T-, B-, and NK-cell compartments was low or absent for most genes. This pattern is consistent with previous gout-focused single-cell studies and supports a myeloid-centered interpretation of the prioritized inflammatory module [55,56].

Feature plots further corroborated that expression signals for *TLR4*, *TLR2*, *TIRAP*, *MSR1*, *CCL2*, *CXCL8*, *MUC1*, *IL1B*, *MYD88*, *TIRAP*, and *IRAK1* were concentrated mainly in monocyte and neutrophil clusters (Figure 3A), again pointing to myeloid rather than lymphoid localization of the prioritized genes [57].

To further document the single-cell findings, we provided supplementary analyses including the group-level UMAP distribution, *TLR4*-axis-related gene expression patterns, overall immune cell-type proportions, and monocyte subtype proportions in flare and remission samples (Supplementary Figures S1–S4 and Supplementary Table S5). These results offer additional context for the myeloid-centered interpretation of the prioritized inflammatory axis. No statistically significant global differences in overall immune cell composition were observed between the two clinical states.

Rather than serving as standalone proof of mechanism, these patterns place the prioritized *TLR4*-axis genes in the cellular context most relevant to gout-associated inflammatory activity and support their subsequent evaluation in genetic and structural analyses.

### 3.5. Mendelian Randomization Analyses Provide Additional Genetic Support for Prioritized TLR4-Axis Genes

To examine whether genetically predicted expression of the prioritized genes was associated with serum uric acid (SUA) levels, we performed two-sample Mendelian randomization analyses for *MSR1*, *CXCL8*, *TLR4*, and *TLR 6* using eQTL-derived instruments. The main manuscript figures are based on the European-ancestry SUA dataset ebi-a-GCST90018977, with inverse-variance weighted (IVW) estimates as the primary results and MR-Egger and leave-one-out analyses used as sensitivity checks [58].

**Figure 4:**
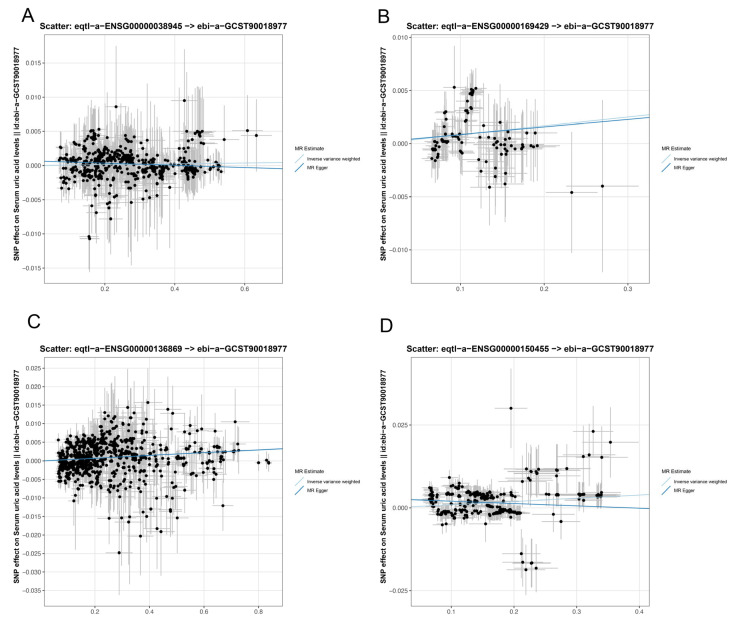
Scatter plots of the main two-sample Mendelian randomization analyses evaluating associations between genetically predicted gene expression and serum uric acid levels in the primary SUA dataset (ebi-a-GCST90018977). (**A**) *MSR1*; (**B**) *CXCL8*; (**C**) *TLR4*; (**D**) *TIRAP*. The x-axis shows SNP effects on gene expression and the y-axis shows SNP effects on serum uric acid. Black lines indicate IVW estimates and blue lines indicate MR-Egger estimates.

For *MSR1*, the IVW analysis indicated a positive association between genetically predicted expression and SUA levels (beta = 0.00402, SE = 0.00026, nsnp = 1219, *p* = 3.40 × 10^−52^; Figure 4A). MR-Egger yielded an estimate in the same direction (beta = 0.00730, SE = 0.00064, *p* = 7.40 × 10^−29^). Sensitivity analyses showed no heterogeneity (Q_p = 0.999), a significant MR-Egger intercept (*p* = 1.20 × 10^−8^), and a non-significant MR-PRESSO global test (*p* = 0.9995), supporting relative signal stability. Steiger analysis also supported the hypothesized direction.

For *CXCL8*, the IVW analysis also supported a positive association with SUA levels (beta = 0.01696, SE = 0.00162, nsnp = 175, *p* = 1.60 × 10^−25^; Figure 4B). The MR-Egger estimate remained directionally consistent (beta = 0.01425, SE = 0.00604, *p* = 0.0193). Sensitivity analyses further showed no significant heterogeneity (Q_p = 0.304), no Egger intercept evidence of pleiotropy (*p* = 0.642), and a non-significant MR-PRESSO global test (*p* = 0.3365).

For *TLR4*, the IVW analysis supported a positive association between genetically predicted expression and SUA levels (beta = 0.00315, SE = 0.00031, nsnp = 1633, *p* = 3.85 × 10^−24^; Figure 4C). MR-Egger gave a directionally similar estimate (beta = 0.00288, SE = 0.00057, *p* = 4.87 × 10^−7^). Sensitivity analyses indicated marked heterogeneity (Q_p = 1.08 × 10^−54^) but no Egger intercept evidence of pleiotropy (*p* = 0.521), with Steiger support.

For *TIRAP*, the IVW analysis yielded a positive association with SUA levels (beta = 0.00927, SE = 0.00087, nsnp = 484, *p* = 3.00 × 10^−26^; Figure 4D). However, the MR-Egger estimate was directionally inconsistent and negative (beta = −0.00749, SE = 0.00263, *p* = 0.00457), indicating that this signal should be interpreted more cautiously than those for *MSR1*, *CXCL8*, and *TLR4*. This inconsistency was reinforced by sensitivity analyses showing significant heterogeneity (Q_p = 2.80 × 10^−14^) and a significant Egger intercept (*p* = 4.99 × 10^−11^), although the MR-PRESSO distortion test was non-significant (*p* = 0.926). Biologically, *TIRAP* is an adaptor molecule within TLR-related inflammatory signaling rather than a direct receptor or urate-handling effector, so its genetically predicted expression may capture more context-dependent or indirect relationships with serum uric acid in humans. Therefore, the *TIRAP* result was retained as a lower-confidence line of genetic support rather than being interpreted at the same confidence level as the more directionally concordant signals for *MSR1*, *CXCL8*, and *TLR4*.

Overall, these MR analyses provided the strongest and directionally consistent support for *MSR1*, *CXCL8*, and *TLR4* in the SUA dataset, as the IVW and MR-Egger estimates were concordant for these genes. In contrast, the *TIRAP* signal was less robust because the MR-Egger estimate was directionally inconsistent with the IVW result, indicating that this association should be interpreted more cautiously. The supplementary heterogeneity, MR-Egger intercept, MR-PRESSO, Steiger, funnel, leave-one-out, and forest results provide context for the stability of these gene-specific signals, but they do not eliminate the interpretive limitations of MR. Therefore, the MR layer was treated as orthogonal genetic support for candidate prioritization rather than as definitive proof that all prioritized genes act through a common mechanism or uniform direction of effect on serum uric acid [51,59].

Detailed IVW estimates, confidence intervals, heterogeneity statistics, MR-Egger intercept tests, MR-PRESSO, and Steiger tests are in Supplementary Table S6. The forest plots, funnel plots, and leave-one-out plots are provided in Supplementary Figure S5.

### 3.6. GutMGene Provided Orthogonal Gut Microbe-Metabolite Support for the Prioritized TLR4 Axis

**Figure 5:**
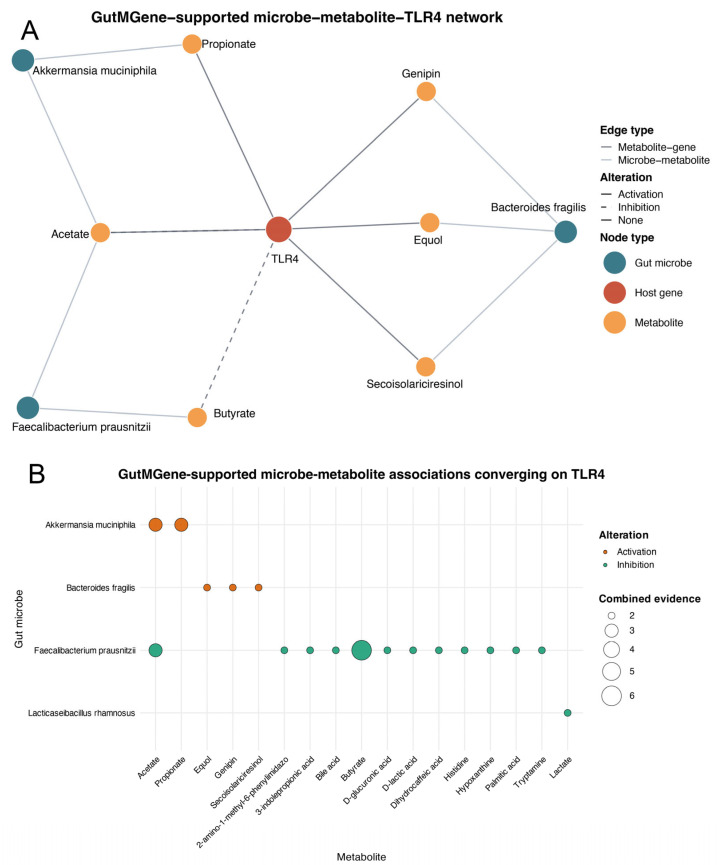
GutMGene provides orthogonal gut microbe-metabolite support for the prioritized *TLR4* axis. (**A**) Tripartite network of GutMGene-curated human associations connecting gut microbes, microbial metabolites, and *TLR4*. Node colors indicate gut microbes, metabolites, and the host gene, whereas edge types indicate microbe-metabolite and metabolite-gene relationships; line style denotes the annotated alteration direction. (**B**) Bubble plot summarizing GutMGene-supported microbe-metabolite associations converging on *TLR4*. Bubble color denotes activation or inhibition, and bubble size represents the combined number of curated associations supporting each microbe-metabolite pair. This analysis was used as a supplementary, literature-based extension of the host-prioritized *TLR4* findings.

Given that *TLR4* emerged as a prioritized host target in our transcriptomic integration, single-cell analysis, and MR framework, we next sought orthogonal evidence from a curated gut microbe-metabolite resource rather than using the microbiome as a primary discovery layer [34,35]. Querying GutMGene identified a *TLR4*-centered support network linking several gut microbes and metabolites, including *Akkermansia muciniphila* with acetate and propionate, Bacteroides fragilis with equol, genipin and secoisolariciresinol, *Faecalibacterium prausnitzii* with butyrate and additional inhibitory metabolites, and *Lacticaseibacillus rhamnosus* with lactate (Figure 5). These metabolites were highlighted because they represented recurrent curated associations converging on *TLR4* through the core gut microbes retained from the upstream workflow, with emphasis placed on associations showing clearer directional consistency or repeated support within the GutMGene resource. The support map further showed that these curated associations converged on *TLR4* with both activation- and inhibition-type annotations, with a particularly prominent inhibitory signal centered on *F. prausnitzii*-associated butyrate based on the cumulative recurrence of inhibitory curated associations in the dataset.

Importantly, this analysis was interpreted as a complementary extension of the host-side findings rather than evidence for a renal cellular source of *TLR4*. In our single-cell analysis, *TLR4* was mainly localized to myeloid immune cells in PBMC samples during gout flares, whereas GutMGene captures literature-curated gut-derived upstream associations. Thus, the GutMGene results do not change the host-side conclusion that *TLR4* is a prioritized inflammatory hub; instead, they suggest that the prioritized *TLR4* axis may also be embedded within a broader gut microbe–metabolite–immune context capable of shaping extra-intestinal inflammatory responses [60,61]. Notably, the prominent *F. prausnitzii*-butyrate signal is directionally consistent with prior evidence linking this organism to kidney protection through a butyrate-dependent gut-kidney axis [62].

### 3.7. Guided Docking Nominates Fucoidan and Alginate as Candidate TLR4-Interacting NSPs

The interaction potential between representative NSPs (fucoidan and alginate) and *TLR4* was explored through three sequential docking rounds using CB-Dock2, followed by PLIP-based interaction profiling [63].

In Round 1 (blind docking), the hexasaccharide repeating units of fucoidan and alginate were placed in the large central cavity of *TLR4* (PDB ID: 3FXI), overlapping the region occupied by the co-crystallized ligand [64]. However, because this cavity is largely hydrophobic and PLIP did not detect specific non-covalent contacts in the resulting poses [65], these models were interpreted as non-informative rather than supportive of biologically plausible binding.

In Round 2 (cavity-guided docking on the cleaned *TLR4* structure after removal of LPS and other heteroatoms), no viable poses were obtained for either polysaccharide within the canonical cavity, further indicating that this region is unlikely to represent a favorable interaction site for these highly anionic ligands in isolation [66].

**Figure 6:**
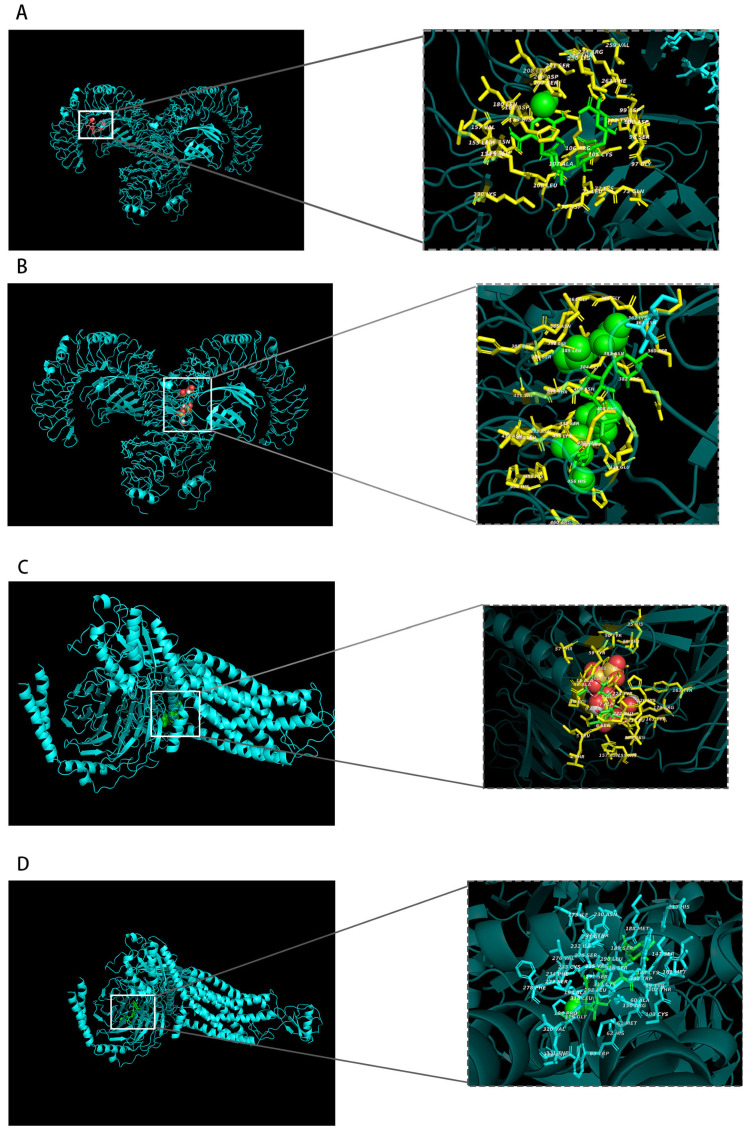
Molecular docking and interaction analysis of fucoidan and alginate in exploratory docking models. (**A**) fucoidan–*TLR4* model and enlarged surface-oriented interaction region. (**B**) alginate–*TLR4* model and enlarged surface-oriented interaction region. (**C**) fucoidan–*CXCL8* exploratory docking model. (**D**) Alginate–*CXCL8* exploratory docking model. The *CXCL8* poses lacked PLIP-supported contacts and were therefore interpreted as non-supportive docking outputs rather than specific interaction models. In all panels, the protein is shown as a cyan cartoon representation, the residues surrounding the predicted docking region are shown as yellow sticks, and the docked ligand poses are highlighted in contrasting colors. The white boxes indicate the regions enlarged in the right-hand panels, and the gray lines connect the overview models to the corresponding zoomed views. The *CXCL8* poses lacked PLIP-supported contacts and were therefore interpreted as non-supportive docking outputs rather than specific interaction models.

In Round 3 (electrostatic-guided docking using positively charged surface residues as anchors), fucoidan yielded clustered poses with multiple hydrogen bonds and salt-bridge-like contacts involving residues such as LYS122, THR319, LYS362, SER120, ASP294, SER504, GLN505, ASN526, ASP550, ASN575, TYR296, and SER552 (Figure 6A; Supplementary Table S7). These models place fucoidan on a positively charged surface region rather than within the canonical hydrophobic cavity, making them more consistent with the physicochemical properties of the ligand [67].

Alginate produced a comparable electrostatic-guided interaction pattern, with hydrogen-bond and salt-bridge-like contacts involving LYS122, SER120, TYR296, THR319, LYS362, ASP294, and LYS341 (Figure 6B; Supplementary Table S8). Together, these guided docking results do not establish direct binding under physiological conditions, but they nominate fucoidan and alginate as plausible *TLR4*-interacting NSPs whose polyanionic character may favor surface electrostatic engagement [68,69,70].

By contrast, analogous docking attempts against *CXCL8* yielded poses without PLIP-supported interactions, suggesting that the *CXCL8* results were likely docking artefacts rather than evidence of a specific interaction. A likely structural explanation for this contrast is that *TLR4* presents a broader positively charged surface environment that is more compatible with multivalent electrostatic contacts from large polyanionic NSPs, whereas *CXCL8* is a much smaller chemokine whose glycosaminoglycan-binding behavior depends on a more limited basic surface patch and on chemokine-specific structural context such as oligomeric state. Thus, the *TLR4* results are more compatible with surface-oriented electrostatic association, while the *CXCL8* docking poses were less supportive of a stable interaction under the present modeling framework [71].

### 3.8. Structural Dynamics Analyses Suggest Altered MD-2-Region Flexibility upon Candidate NSP Binding

**Figure 7:**
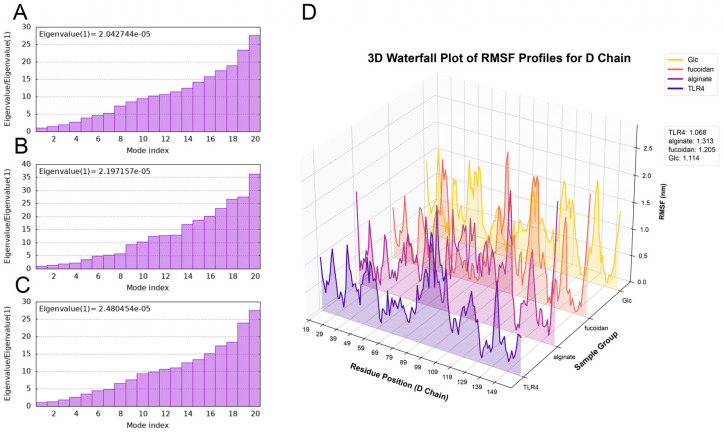
Effect of NSP binding on *TLR4* structural dynamics (normal mode analysis, iMODS server, and CABSflex). (**A**) Eigenvalue spectrum of alginate–*TLR4* complex (Eigenvalue (1) = 2.042744 × 10^−05^). (**B**) Eigenvalue spectrum of fucoidan–*TLR4* complex (Eigenvalue (1) = 2.197157 × 10^−05^). (**C**) Eigenvalue spectrum of apo–*TLR4* (Eigenvalue (1) = 2.480454 × 10^−05^). (**D**) RMSF comparison of *TLR4* chain D (MD-2 domain, residues 19-158). Green curve: alginate–*TLR4* complex; blue curve: fucoidan–*TLR4* complex; red curve: apo–*TLR4* (unliganded); purple curve: neutral-(1,4)-linked glucose hexasaccharide control (Glc). The inset lists the mean RMSF of the top 50% most mobile residues for each system.

Normal mode analysis revealed differences in the collective motion patterns among the three structures. Compared with the apo–*TLR4* structure (3FXI), the first eigenvalue (Eigenvalue (1)) of the alginate––*TLR4* and fucoidan–*TLR4* complexes was reduced (alginate group: 2.042744 × 10^−05^; fucoidan group: 2.197157 × 10^−05^; 3FXI group: 2.480454 × 10^−05^), indicating that ligand binding decreases the overall motional stiffness of the structure and facilitates low-frequency collective deformation (Figure 7A–C).

The main-chain deformability plots showed prominent peaks in the 3FXI structure at approximately atom indices 600 and 1200. In the alginate-bound complex, these peaks were further elevated (some approaching 1.0). In the fucoidan-bound complex, more dispersed and higher peaks appeared, particularly in the 1000–4000 interval, suggesting increased flexibility in specific residue regions that may serve as structural hinge points.

RMSF analysis of chain D (MD-2, residues 19-158) further supported altered local dynamics (Figure 7D). The mean RMSF (top 50% high values) increased from 1.068 in apo–*TLR4* to 1.205 in the fucoidan-bound complex and 1.313 in the alginate-bound complex, whereas a neutral α-1-4-linked glucose hexasaccharide control produced a smaller increase (1.114). These comparisons suggest that the anionic polysaccharides are associated with greater MD-2-region flexibility than either the apo structure or a size-matched neutral control.

In the B-factor/Mobility comparison plots, the NMA-calculated values were consistent with the experimental PDB values of apo–*TLR4* group; however, the alginate-bound group and fucoidan-bound group exhibited greater fluctuation amplitudes in the corresponding flexible regions than the 3FXI group (see Supplementary Figure S6), indicating enhanced atomic displacement potential. The variance contribution plots showed that the cumulative contribution of the low-frequency modes to overall equilibrium motion rapidly approached 100% in all three groups, with slight adjustments in the individual contributions of low-frequency modes in the bound groups, consistent with the eigenvalue changes.

The covariance matrix revealed distinct blue anti-correlated motion regions in the 3FXI and alginate groups, reflecting complexity in the directionality of inter-residue motions; the fucoidan-bound group displayed a predominantly red positive-correlation pattern, indicating more uniform residue motion coupling (see Supplementary Figure S6).

Overall, the normal mode and RMSF analyses suggest that fucoidan- and alginate-associated models are accompanied by altered flexibility in the *TLR4*/MD-2 region, with a stronger effect than that observed for the neutral glucose control. These structural-dynamic observations are consistent with, but do not prove, the possibility that polyanionic NSPs could influence *TLR4*-related conformational states relevant to downstream signaling [72,73].

### 3.9. Exploratory ADMET Profiling of Candidate NSPs

Exploratory ADMET profiling using pkCSM suggested that fucoidan and alginate share several features expected for large polysaccharides [41], including moderate water solubility, negligible predicted intestinal absorption, low blood–brain barrier penetration, no major CYP450 inhibition signals, and favorable AMES, hepatotoxicity, and hERG predictions [68,74], broadly consistent with prior discussions of fucoidan and alginate pharmacological limitations and safety considerations [74,75,76]. These results are useful mainly as a coarse safety screen and should not be overinterpreted as evidence of drug-likeness or target engagement; rather, they indicate that the two representative NSPs do not show obvious computational toxicity liabilities within the limits of the model. Complete results of fucoidan and alginate are provided in the Supplementary Materials (Supplementary Tables S9 and S10).

### 3.10. In Silico TLR4 Perturbation Suggests a Possible Link to Renal Urate-Handling Programs

**Figure 8:**
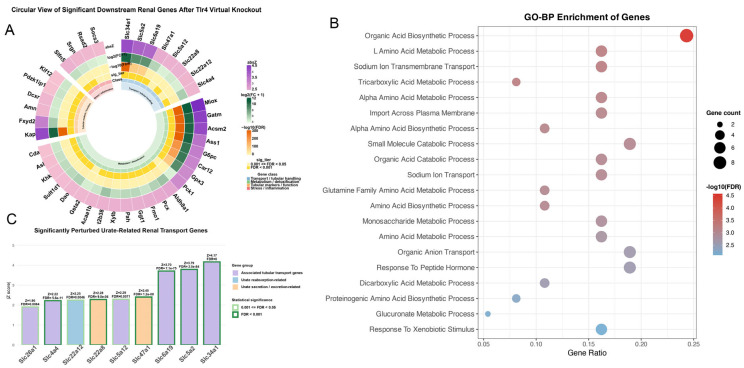
Virtual knockout of *TLR4* disrupts renal transport and metabolic programs in proximal tubular cells. (**A**) Circular multi-track view of significantly perturbed downstream renal genes after *TLR4* virtual knockout, displaying absZ, log2(FC + 1), −log10(FDR), significance tier, and functional class. Genes are grouped into transport/tubular handling, metabolism/detoxification, tubular markers/function, and stress/inflammation categories. (**B**) GO biological process enrichment analysis of the significantly perturbed genes identified after *TLR4* virtual knockout. Dot size indicates gene count, dot color represents −log10(FDR), and the x-axis shows gene ratio. (**C**) Significantly perturbed urate-related renal transport genes after *TLR4* virtual knockout. Bar height indicates absolute Z score, bar fill denotes gene group, and border color denotes statistical significance.

As an extension analysis, we performed in silico *Tlr4* knockout in a proximal tubule-enriched single-cell dataset using scTenifoldKnk to explore whether *Tlr4* perturbation might be linked to renal urate-handling programs [43]. As shown in Figure 8A, virtual deletion of *Tlr4* perturbed a broad set of downstream genes, with the strongest signals mapping to transport/tubular handling, metabolism/detoxification, tubular markers/function, and stress/inflammation categories. Among these were several proximal tubular transport-associated genes, including *Slc34a1*, *Slc5a2*, *Slc6a19*, *Slc47a1*, *Slc22a8*, *Slc22a12*, and *Slc4a4*.

Notably, the urate-related transporters *Slc22a12* and *Slc22a8* were significantly perturbed following *TLR4* virtual knockout (*Slc22a12*, Z = 2.25, FDR = 0.0046; *Slc22a8*, Z = 2.28, FDR = 9.0 × 10^−4^; Figure 8C). This is notable because SLC22A12/URAT1 is a key apical urate reabsorption transporter in the proximal tubule, whereas SLC22A8/OAT3 contributes to renal organic anion uptake and secretion-related handling of urate and other endogenous metabolites [77,78]. *Slc47a1*, *Slc34a1*, and *Slc4a4* were also significantly perturbed, suggesting that the inferred regulatory effect extends beyond a single urate transporter.

Consistent with the gene-level perturbation pattern, over-representation analysis of the significantly perturbed genes highlighted biological processes related to organic acid biosynthetic process, L-amino acid metabolic process, sodium ion transmembrane transport, small molecule catabolic process, and organic anion transport (Figure 8B). The enriched organic anion transport term included *Slc22a12*, *Slc22a8*, *Slc47a1*, *Slc4a4*, and *Slc26a1*, supporting a possible link between *TLR4* perturbation and renal urate-handling programs.

Given previous evidence that soluble uric acid can activate *TLR4*-dependent inflammatory signaling in renal proximal tubular epithelial cells [79], this extension analysis raises the possibility that *TLR4*-centered inflammatory sensing may intersect with tubular transport-metabolic homeostasis. Because this result is derived from in silico perturbation in a mouse kidney dataset rather than direct functional validation, it should be interpreted as hypothesis-generating support for future renal follow-up studies.

## 4. Discussion

This study applied an integrative bioinformatics and computational framework to prioritize candidate NSP-associated targets in hyperuricemia-associated inflammation. Across transcriptomic, network, enrichment, single-cell, Mendelian randomization, docking, structural-dynamics, and virtual perturbation analyses, the main signal most consistently pointed to a *TLR4*-centered inflammatory axis rather than a broad and heterogeneous set of candidate genes. In this context, *TLR4*, together with *MSR1*, *TIRAP*, and *CXCL8*, emerged as a compact candidate module linking HUA-related inflammatory transcriptional programs to NSP-oriented target prioritization, but this prioritization should be interpreted as computational ranking rather than direct mechanistic confirmation.

The network and enrichment results, together with the single-cell analyses, are important because they move the study beyond a simple overlap list. The prioritized genes were not only shared across datasets but were also organized into a coherent innate immune program enriched for Toll-like receptor, NF-κB, IL-17, and related inflammatory pathways, and were localized mainly to myeloid compartments during gout flares. This pattern supports a myeloid-centered interpretation of the prioritized inflammatory module and strengthens the case for treating *TLR4* as the most coherent organizing node across multiple analytical levels, although such convergence may still be influenced by database annotation density, pathway redundancy, and the tendency of inflammatory hub genes to recur across heterogeneous immune-related datasets.

As a supplementary extension, we also queried GutMGene, a curated resource linking gut microbes and microbial metabolites to host genes. This analysis did not alter the main discovery axis of the study, which was established from host-side transcriptomic, network, single-cell, and Mendelian-randomization evidence, but instead provided orthogonal support for the biological plausibility of a *TLR4*-centered axis. Several gut microbial metabolites converged on *TLR4*, with a notable inhibitory pattern centered on Faecalibacterium prausnitzii-associated butyrate [80]. However, this signal should be interpreted as contextual support rather than competing evidence, because our single-cell analysis localized *TLR4* mainly to myeloid compartments, whereas GutMGene captures curated upstream gut-derived associations rather than cell-resolved host expression evidence.

The docking analyses should be interpreted cautiously. Blind and cavity-guided docking did not support a convincing interaction model within the canonical hydrophobic cavity, whereas electrostatic-guided docking yielded more plausible surface-oriented poses for fucoidan and alginate. These guided models, supported by PLIP-detected hydrogen bonds and salt-bridge-like contacts, do not establish direct receptor antagonism, but they do suggest that highly anionic NSPs may be capable of electrostatic engagement with positively charged regions of the *TLR4* surface. This restrained interpretation is more defensible than claiming stable physiological binding and is consistent with the known importance of charge-sensitive interactions in *TLR4*/MD-2-related recognition processes [64,72,73]. However, electrostatic-guided docking itself also introduces uncertainty, because it preferentially steers highly charged ligands toward predefined positively charged surface regions and may therefore overrepresent favorable poses that depend strongly on the assumed charge distribution, protonation state, and simplified treatment of solvent and ionic screening. Accordingly, these guided poses should be interpreted as heuristic interaction models rather than as unique or experimentally validated binding conformations. At the same time, docking of large, flexible, and highly polyanionic polysaccharides remains methodologically challenging, because pose prediction is sensitive to ligand simplification, protonation assumptions, conformational sampling, and the use of guided restraints; therefore, these results should be regarded as plausibility models rather than quantitative evidence of affinity or specificity.

The structural-dynamics analyses provide a second, but still indirect, layer of support. Relative to apo–*TLR4* and a size-matched neutral glucose control, fucoidan- and alginate-associated models showed greater flexibility in the MD-2 region, suggesting that polyanionic NSPs may alter local conformational behavior more strongly than steric size alone would predict. In addition, the reduced low-frequency eigenvalues and the altered RMSF pattern are consistent with a decrease in overall motional stiffness and an increase in collective conformational plasticity, particularly in the *TLR4*/MD-2 region. Because *TLR4* signaling depends on ligand-sensitive conformational organization of the *TLR4*/MD-2 complex and the transmission of structural changes to receptor dimerization-competent states, these computational shifts may be biologically relevant as indicators of altered signaling propensity rather than merely local flexibility changes. Even so, the present analyses do not establish whether such changes would favor receptor activation, destabilization, or nonproductive perturbation under physiological conditions [72]. Exploratory ADMET profiling similarly serves only as a coarse screen: it indicates an absence of obvious computational toxicity liabilities under the chosen model, but it does not convert these polysaccharides into conventional drug-like molecules. Taken together, these analyses are best viewed as nomination tools that help rank candidate NSPs for follow-up testing. In addition, normal mode analysis and related flexibility simulations capture simplified features of structural motion under idealized conditions and cannot reproduce the full biophysical complexity of receptor activation in membrane-associated and ligand-competitive environments. Likewise, ADMET prediction platforms are primarily trained on small-molecule-like chemical spaces, so their applicability to macromolecular polysaccharides is limited and should be interpreted only as exploratory reference information.

An additional strength of the study is the inclusion of the in silico *TLR4* knockout analysis, which extends the manuscript beyond inflammatory blood-cell signatures [43]. The observed perturbation of Slc22a12, Slc22a8, Slc47a1, Slc34a1, and Slc4a4 suggests a possible connection between *TLR4*-centered regulation and renal urate-handling programs. This is biologically intriguing because SLC22A12/URAT1 and SLC22A8/OAT3 occupy central roles in renal urate handling [77,78], and soluble uric acid has been reported to activate *TLR4*-dependent inflammatory signaling in proximal tubular epithelial cells [79]. Even so, the current result should be treated as an extension analysis that generates a renal hypothesis, not as direct proof that NSP-mediated *TLR4* modulation lowers serum uric acid through these transporters. This caution is especially important because virtual knockout infers regulatory perturbation from transcriptomic network structure rather than from experimentally observed gene deletion, and the resulting downstream shifts may depend on cell-state composition, model assumptions, and the quality of the input single-cell dataset. More specifically, this strategy assumes that the inferred single-cell regulatory architecture provides a reasonable approximation of the underlying biological system and that computational removal of *TLR4*-associated influence can capture part of the downstream perturbation pattern expected after loss of function. However, unlike biological knockout models, virtual perturbation cannot reproduce developmental compensation, temporal adaptation, multicellular feedback, or tissue-level physiological interactions, and therefore should be interpreted as a hypothesis-generating approximation rather than a substitute for experimental gene deletion.

The main contribution of this work is therefore not the claim that *TLR4* is newly implicated in hyperuricemia or gout, because previous experimental and bioinformatics studies have already linked *TLR4*-related signaling to these conditions. Rather, the contribution of the present study lies in constructing an NSP-oriented integrative screening framework. Within this framework, *TLR4* emerged as the most coherent candidate hub across multiple analytical layers, the relevant signal was localized mainly to myeloid compartments during gout flares, and fucoidan and alginate were nominated as representative NSPs for follow-up investigation. Compared with earlier bioinformatics studies that mainly reported disease-associated inflammatory genes or pathways, this framework more directly connects target prioritization with candidate polysaccharide selection and mechanistic follow-up planning.

Several limitations should be made explicit. First, the study integrates heterogeneous public datasets spanning human blood, cell-line perturbation experiments, acute gout PBMC single-cell data, and mouse kidney single-cell data, so biological context is not uniform across all analytical layers. These datasets differ in species background, tissue source, disease stage, inflammatory context, and processing pipeline, and they were not generated as a single harmonized cohort. Accordingly, the present workflow should be understood as an evidence-integration and prioritization strategy across related but non-identical biological contexts, rather than as a formal cross-cohort meta-analysis proving one uniform mechanism. In addition, the GutMGene component is based on a curated cross-study knowledge resource rather than matched tissue-resolved samples from the same system and should therefore be interpreted as supplementary context rather than direct mechanistic validation. Second, the docking and normal mode analyses rely on simplified structural models and guided constraints, which are useful for hypothesis generation but insufficient for claiming binding specificity or receptor inhibition. This limitation is particularly relevant for NSPs, whose high molecular weight, branching patterns, charge heterogeneity, and conformational flexibility are difficult to model accurately in standard docking workflows. Although nanosecond-scale molecular dynamics simulations could provide more detailed information on complex stability and time-dependent conformational behavior, they were not included in the present study. Instead, guided docking, normal mode analysis, and RMSF-based flexibility assessment were used as preliminary structural support within a target-prioritization framework. This choice was considered appropriate for the current study because *TLR4*/MD-2 signaling is closely linked to conformational organization and local flexibility in the MD-2 region, whereas long-timescale simulations of large, flexible, and highly charged polysaccharides would still depend strongly on the assumed initial docking pose in the absence of experimental binding-site validation. Third, the Mendelian randomization analysis should also be interpreted cautiously. It evaluates associations between genetically predicted gene expression and serum uric acid rather than directly testing NSP exposure, receptor occupancy, or downstream anti-inflammatory efficacy. Therefore, it supports only one segment of the proposed biological chain rather than providing self-sufficient causal proof. Its interpretation may also be affected by potential horizontal pleiotropy, tissue mismatch between available eQTL resources and the disease-relevant cell populations, ancestry-specific characteristics of the summary statistics, and the strength and robustness of the selected instruments in sensitivity analyses. The discordant IVW and MR-Egger estimates for *TIRAP* further illustrate that not all prioritized inflammatory genes showed equally stable genetic support. In particular, because *TIRAP* functions as a signaling adaptor in a context-dependent innate immune pathway, its association with serum uric acid may be more vulnerable to indirect regulation, pleiotropic influences, or method-specific sensitivity than the more concordant signals observed for *MSR1*, *CXCL8*, and *TLR4*. Finally, no direct biochemical, cellular, or in vivo validation is yet available to confirm *TLR4* antagonism, downstream pathway suppression, or uric acid-lowering efficacy. For this reason, the current study should be framed as hypothesis-generating and target-prioritizing rather than mechanism-proving. A further limitation concerns the curation of the NSP-related receptor set. Because receptor selection was based on ligand annotation in PRRDB 2.0, the final candidate space depends on database completeness, annotation accuracy, and literature coverage, and may therefore favor well-studied receptors while underrepresenting less-characterized NSP-responsive candidates. In addition, ligand-based receptor annotation simplifies the biological complexity of receptor-polysaccharide interactions, and symbol harmonization or removal of ambiguous entries may have introduced some loss of information.

Overall, the study provides a coherent computational rationale for prioritizing the *TLR4* axis in HUA-related inflammation and for nominating fucoidan and alginate as representative NSPs for follow-up investigation. The next decisive experiments would be direct binding and competition assays, cell-based tests of *TLR4*/NF-κB pathway activity, and renal transporter validation in hyperuricemia-relevant models. Until such experiments are completed, the present findings should be regarded as structured, hypothesis-generating evidence rather than definitive proof of mechanism or therapeutic efficacy.

## 5. Conclusions

This integrative bioinformatics and computational study does not primarily contribute by newly linking *TLR4* to hyperuricemia-associated inflammation, as *TLR4* has already been implicated in prior studies. Instead, its main contribution is the development of an NSP-oriented multi-layer prioritization framework through which the *TLR4* axis emerged as the most coherent candidate hub, together with the nomination of fucoidan and alginate as representative NSPs for follow-up validation.

In this framework, fucoidan and alginate emerged as representative NSPs for follow-up investigation. Under electrostatic-guided docking conditions, both polysaccharides yielded plausible *TLR4* interaction models, and structural-dynamics analyses suggested altered flexibility in the MD-2 region. The virtual knockout extension further raised the possibility that *TLR4*-centered signaling may intersect with renal urate-handling programs.

Overall, these results do not establish direct receptor antagonism or uric acid-lowering efficacy, but they provide a structured basis for prioritizing experiments. Future work should focus on direct binding validation, pathway-level functional assays, and renal transporter studies in hyperuricemia-relevant systems to determine whether the computationally prioritized *TLR4* axis can be translated into a substantiated NSP mechanism.

## Data Availability

The original contributions presented in this study are included in the article and Supplementary Materials. Publicly available datasets were analyzed in this study, including GEO datasets GSE186871, GSE189228, GSE190205, GSE198133, GSE205963, GSE214587, GSE242872, GSE262687, GSE300922, and GSE211783, as well as the OpenGWAS dataset and the GutMGene database. Further inquiries can be directed to the corresponding author.

## Supplementary Materials

The following supporting information can be downloaded at: https://www.mdpi.com/article/10.3390/biology15141150/s1, Figure S1: Group-level expression distributions of selected prioritized inflammatory genes in PBMCs from patients with acute gout during flare and remission in the GSE211783 dataset. Jittered expression plots are shown for TLR4, TLR2, TLR6, MSR1, CCL2, CXCL8, MUC1, IL1B, MYD88, TIRAP, and IRAK1, providing supplementary context for the expression patterns of the prioritized TLR4-axis-related genes across the two clinical states; Figure S2: Group-level UMAP distribution of PBMCs from patients sampled during acute gout flare and remission in the GSE211783 dataset. Cells from the two clinical states are broadly intermingled in the low-dimensional space, indicating that global clustering structure is driven predominantly by cell-type identity rather than by overall condition-specific separation; Figure S3: Comparison of overall immune cell-type proportions between flare and remission samples in the GSE211783 PBMC dataset. Boxplots show the estimated proportions of major immune and minor myeloid cell populations, with Wilcoxon test *p* values annotated in each panel. No statistically significant global differences in overall immune cell composition were observed between the two clinical states; Figure S4: Comparison of monocyte subcluster proportions between flare and remission samples in the GSE211783 PBMC dataset. Boxplots display the relative proportions of individual monocyte subclusters, with Wilcoxon test *p* values shown in each panel. No statistically significant differences in monocyte subtype composition were observed between flare and remission samples; Figure S5: Mendelian randomization analysis evaluating the causal effects of key genes on serum uric acid levels. (A) MSR1 forest plot: MR estimates of individual SNPs and overall effect. (B) MSR1 funnel plot: assessment of directional pleiotropy. (C) MSR1 leave-one-out sensitivity analysis plot: showing the stability of causal estimates after removing individual SNPs one by one. (D) CXCL8 forest plot: MR estimates of individual SNPs and overall effect. (E) CXCL8 funnel plot: assessment of directional pleiotropy. (F) CXCL8 leave-one-out sensitivity analysis plot: showing the stability of causal estimates after removing individual SNPs one by one. (G) TLR4 forest plot: MR estimates of individual SNPs and overall effect. (H) TLR4 funnel plot: assessment of directional pleiotropy. (I) TLR4 leave-one-out sensitivity analysis plot: showing the stability of causal estimates after removing individual SNPs one by one. (J) TIRAP forest plot: MR estimates of individual SNPs and overall effect. (K) TIRAP funnel plot: assessing directional pleiotropy. (L) TIRAP leave-one-out sensitivity analysis plot: showing the stability of causal estimates after removing individual SNPs one by one; Figure S6: (A) Deformability plot of the unbound TLR4 (3FXI) structure, atom index range 0–1400. (B) Deformability plot of the Alginate-TLR4 complex, atom index range 0–1400. (C) Deformability plot of the Fucoidan-TLR4 complex, atom index range of 0–7000. (D) B-factor/Mobility comparison plot of the alginate–TLR4 complex, with NMA-calculated values (red) and experimental PDB values (black). (E) B-factor/Mobility comparison plot of the fucoidan–TLR4 complex. (F) B-factor/Mobility comparison plot of the unbound TLR4 structure (3FXI). (G) Covariance matrix of the unbound TLR4 structure (3FXI), showing anti-correlated motion regions (blue). (H) Covariance matrix of the alginate–TLR4 complex, showing anti-correlated motion regions (blue). (I) Covariance matrix of the fucoidan–TLR4 complex, showing predominantly positive-correlation pattern (red); Table S1: Complete Gene Ontology biological process (GO-BP) enrichment results for the prioritized candidate gene set, including term names, gene counts, adjusted *p*-values, fold enrichment, and associated genes; Table S2: Complete Gene Ontology cellular component (GO-CC) enrichment results for the prioritized candidate gene set, including term names, gene counts, adjusted *p*-values, fold enrichment, and associated genes; Table S3: Complete Gene Ontology molecular function (GO-MF) enrichment results for the prioritized candidate gene set, including term names, gene counts, adjusted *p*-values, fold enrichment, and associated genes; Table S4: Complete KEGG pathway enrichment results for the prioritized candidate gene set, including pathway names, gene counts, adjusted *p*-values, fold enrichment, and associated genes; Table S5: Cell-type proportions were compared between the flare and remission groups using the Wilcoxon test. Raw *p* values, adjusted *p* values, formatted *p* values, and significance annotations are reported as generated by the original statistical output. ns indicates not significant; Table S6. (A) Heterogeneity statistics for four MR-prioritized genes. (B) MR-PRESSO sensitivity results for four MR-prioritized genes. (C) MR-Egger intercept tests for horizontal pleiotropy. (D) Integrated sensitivity summary for four MR-prioritized genes; (E) Steiger directionality tests for four MR-prioritized genes; Table S7: Summary table of main noncovalent interactions of fucoidan-TLR4 (PLIP analysis, included interaction type, residues, key parameters and notes); Table S8: Summary table of main noncovalent interactions with alginate-TLR4 (PLIP analysis, included interaction type, residues, key parameters and notes); Table S9: ADMET prediction results for Fucoidan (pkCSM model). This table shows the main pharmacokinetic (ADMET) prediction parameters of the candidate non-starch polysaccharides (Fucoidan). The prediction is completed using the pkCSM online tool, and the input is the canonical molecular structure generated from the corresponding chemical descriptors; Table S10: ADMET prediction results for Alginate (pkCSM model). This table shows the main pharmacokinetic (ADMET) prediction parameters of the candidate non-starch polysaccharides (Alginate). The prediction is completed using the pkCSM online tool, and the input is the canonical molecular structure generated from the corresponding chemical descriptors. Supplementary material has been submitted alongside the manuscript, including figures and tables that support the conclusions of this paper.

## Abbreviations

The following abbreviations are used in this manuscript:

HUA	Hyperuricemia
UA	uric acid
NSPs	Non-starch polysaccharides
DEGs	Different expression genes
GO	Gene ontology
KEGG	Kyoto Encyclopedia of Genes and Genomes
*TLR4*	Toll-like receptor 4
MSR1	Macrophage scavenger receptor-1
TIRAP	TIR domain containing adaptor protein
CXCL8	C-X-C motif chemokine ligand 8
NF-κB	Nuclear Factor κ-light-chain-enhancer of activated B cells
MAPK	Mitogen-Activated Protein Kinase
JNK	c-Jun N-terminal Kinase
TNF-α	Tumor Necrosis Factor-alpha
IL-6	Interleukin-6
IL-12	Interleukin-12
SLC22A12	Solute Carrier Family 22 Member 12
URAT1	Urate Transporter 1

## References

[1] El Ridi R., Tallima H. (2017). Physiological Functions and Pathogenic Potential of Uric Acid: A Review. J. Adv. Res..

[2] Ha G., Wu J., Hu J., Wang X., Xie Y., Zhao Z., Cai D. (2025). Molecular and Clinical Perspectives on the Regulation of Sleep and Uric Acid Metabolism. Nat. Sci. Sleep.

[3] De Lima Balico L., Gaucher E.A. (2025). Genomic Insertion of Ancestral Uricase into Human Liver Cells to Determine Metabolic Consequences of Pseudogenization. Sci. Rep..

[4] Borghi C., Domienik-Karłowicz J., Tykarski A., Widecka K., Filipiak K.J., Jaguszewski M.J., Narkiewicz K., Mancia G. (2021). Expert Consensus for the Diagnosis and Treatment of Patient with Hyperuricemia and High Cardiovascular Risk: 2021 Update. Cardiol. J..

[5] McLean L., Dalbeth N. (2015). Etiology and Pathogenesis of Gout. Rheumatology.

[6] Ngandeu-Singwe M., Nkeck J.R., Hamroun A., Endomba F.T., Ngoufack-Tientcheu C., Ndoadoumgue A.L., Fojo B.T., Ngouo A.T., Tounouga D.N., Nkeck J.P. (2026). Worldwide Trends in Hyperuricaemia from 2000 to 2023: A Systematic Review and Modelling Analysis. Lancet Rheumatol..

[7] Liang H., Zhang J., Yu H., Ding L., Liu F., Wang J. (2023). Incidence Density of Hyperuricemia and Association between Metabolism-Related Predisposing Risk Factors and Serum Urate in Chinese Adults: A Cohort Study. Front. Endocrinol..

[8] Baranova A.S., Nesterenko A.V., Paripa K.R., Volkova N.A. (2025). Gout: Febuxostat Versus Allopurinol—Mechanisms of Action and Side Effects. Innov. Sci. Res..

[9] Zhang W., Liu W., Leng F., Shen M., Xie J. (2025). Dietary Non-Starch Plant Polysaccharides: Multi-Mechanisms for Managing Diabetic Microvascular Complications. Carbohydr. Polym..

[10] Wang Z., Wu G., Niu T., Guo Y., Wang C., Wang X., Yu J. (2024). Polysaccharide Isolated from *Dioscorea septemloba* Improves Hyperuricemia and Alleviates Renal Fibrosis through Gut-Kidney Axis in Mice. Int. J. Biol. Macromol..

[11] Zhang K., Sun S., Qiu H., Zhao H., Zhao H., Shen Y., Wang Y., Zhang Y. (2026). Viscum Coloratum Polysaccharide Ameliorates Hyperuricemic Nephropathy by Upregulating Nrf2 to Inhibit TGF-Β1/Smad3 and NLRP3/ASC/Caspase-1 Pathways. J. Ethnopharmacol..

[12] Zhang Y., Tan X., Lin Z., Li F., Yang C., Zheng H., Li L., Liu H., Shang J. (2021). fucoidan from *Laminaria japonica* Inhibits Expression of GLUT9 and URAT1 via PI3K/Akt, JNK and NF-κB Pathways in Uric Acid-Exposed HK-2 Cells. Mar. Drugs.

[13] Yu W., Liu J., Baranenko D., Cifuentes A., Ibañez E., Zhang Y., Lu W. (2025). The Role of Dietary Polysaccharides in Uric Acid Regulation: Mechanisms and Benefits in Managing Hyperuricemia. Trends Food Sci. Technol..

[14] Wei M., Hu Y., Li X., Long X., Zhang Z., Tao X., Wei H. (2026). Exopolysaccharides from *Lactiplantibacillus plantarum* WLPL04 Alleviate Hyperuricemia by Regulating Uric Acid Metabolism and Gut Microbiota. Foods.

[15] Barrett T., Wilhite S.E., Ledoux P., Evangelista C., Kim I.F., Tomashevsky M., Marshall K.A., Phillippy K.H., Sherman P.M., Holko M. (2012). NCBI GEO: Archive for Functional Genomics Data Sets—Update. Nucleic Acids Res..

[16] Wickham H. (2011). The Split-Apply-Combine Strategy for Data Analysis. J. Stat. Softw..

[17] Love M.I., Huber W., Anders S. (2014). Moderated Estimation of Fold Change and Dispersion for RNA-Seq Data with DESeq2. Genome Biol..

[18] Bourgon R., Gentleman R., Huber W. (2010). Independent Filtering Increases Detection Power for High-Throughput Experiments. Proc. Natl. Acad. Sci. USA.

[19] Conesa A., Madrigal P., Tarazona S., Gomez-Cabrero D., Cervera A., McPherson A., Szcześniak M.W., Gaffney D.J., Elo L.L., Zhang X. (2016). A Survey of Best Practices for RNA-Seq Data Analysis. Genome Biol..

[20] Ritchie M.E., Phipson B., Wu D., Hu Y., Law C.W., Shi W., Smyth G.K. (2015). Limma Powers Differential Expression Analyses for RNA-Sequencing and Microarray Studies. Nucleic Acids Res..

[21] Smyth G.K. (2004). Linear Models and Empirical Bayes Methods for Assessing Differential Expression in Microarray Experiments. Stat. Appl. Genet. Mol. Biol..

[22] Durinck S., Spellman P.T., Birney E., Huber W. (2009). Mapping Identifiers for the Integration of Genomic Datasets with the R/Bioconductor Package biomaRt. Nat. Protoc..

[23] Kaur D., Patiyal S., Sharma N., Usmani S.S., Raghava G.P.S. (2019). PRRDB 2.0: A Comprehensive Database of Pattern-Recognition Receptors and Their Ligands. Database.

[24] Szklarczyk D., Kirsch R., Koutrouli M., Nastou K., Mehryary F., Hachilif R., Gable A.L., Fang T., Doncheva N.T., Pyysalo S. (2023). The STRING Database in 2023: Protein–Protein Association Networks and Functional Enrichment Analyses for Any Sequenced Genome of Interest. Nucleic Acids Res..

[25] Shannon P., Markiel A., Ozier O., Baliga N.S., Wang J.T., Ramage D., Amin N., Schwikowski B., Ideker T. (2003). Cytoscape: A Software Environment for Integrated Models of Biomolecular Interaction Networks. Genome Res..

[26] Sherman B.T., Hao M., Qiu J., Jiao X., Baseler M.W., Lane H.C., Imamichi T., Chang W. (2022). DAVID: A Web Server for Functional Enrichment Analysis and Functional Annotation of Gene Lists (2021 Update). Nucleic Acids Res..

[27] Hao Y., Stuart T., Kowalski M.H., Choudhary S., Hoffman P., Hartman A., Srivastava A., Molla G., Madad S., Fernandez-Granda C. (2024). Dictionary Learning for Integrative, Multimodal and Scalable Single-Cell Analysis. Nat. Biotechnol..

[28] Aran D., Looney A.P., Liu L., Wu E., Fong V., Hsu A., Chak S., Naikawadi R.P., Wolters P.J., Abate A.R. (2019). Reference-Based Analysis of Lung Single-Cell Sequencing Reveals a Transitional Profibrotic Macrophage. Nat. Immunol..

[29] Elsworth B., Lyon M., Alexander T., Liu Y., Matthews P., Hallett J., Bates P., Palmer T., Haberland V., Smith G.D. (2020). The MRC IEU OpenGWAS Data Infrastructure. bioRxiv.

[30] Han S.-I., Chang S.H., Lee C., Jeon M.S., Heo Y.M., Kim S., Choi Y.-E. (2020). Astaxanthin biosynthesis promotion with pH shock in the green microalga, *Haematococcus lacustris*. Bioresour. Technol..

[31] Lin S.-H., Brown D.W., Machiela M.J. (2020). LDtrait: An Online Tool for Identifying Published Phenotype Associations in Linkage Disequilibrium. Cancer Res..

[32] Kamat M.A., Blackshaw J.A., Young R., Surendran P., Burgess S., Danesh J., Butterworth A.S., Staley J.R. (2019). PhenoScanner V2: An Expanded Tool for Searching Human Genotype–Phenotype Associations. Bioinformatics.

[33] Verbanck M., Chen C.-Y., Neale B., Do R. (2018). Detection of Widespread Horizontal Pleiotropy in Causal Relationships Inferred from Mendelian Randomization between Complex Traits and Diseases. Nat. Genet..

[34] Qi C., He G., Qian K., Guan S., Li Z., Liang S., Liu J., Ke X., Zhang S., Lu M. (2025). gutMGene v2.0: An Updated Comprehensive Database for Target Genes of Gut Microbes and Microbial Metabolites. Nucleic Acids Res..

[35] Cheng L., Qi C., Yang H., Lu M., Cai Y., Fu T., Ren J., Jin Q., Zhang X. (2022). gutMGene: A Comprehensive Database for Target Genes of Gut Microbes and Microbial Metabolites. Nucleic Acids Res..

[36] Grant O.C., Wentworth D., Holmes S.G., Kandel R., Sehnal D., Wang X., Xiao Y., Sheppard P., Grelsson T., Coulter A. (2026). Generating 3D Models of Complex Carbohydrates with GLYCAM-Web. Nat. Methods.

[37] Liu Y., Yang X., Gan J., Chen S., Xiao Z.-X., Cao Y. (2022). CB-Dock2: Improved Protein–Ligand Blind Docking by Integrating Cavity Detection, Docking and Homologous Template Fitting. Nucleic Acids Res..

[38] Salentin S., Schreiber S., Haupt V.J., Adasme M.F., Schroeder M. (2015). PLIP: Fully Automated Protein–Ligand Interaction Profiler. Nucleic Acids Res..

[39] López-Blanco J.R., Aliaga J.I., Quintana-Ortí E.S., Chacón P. (2014). iMODS: Internal Coordinates Normal Mode Analysis Server. Nucleic Acids Res..

[40] Kuriata A., Gierut A.M., Oleniecki T., Ciemny M.P., Kolinski A., Kurcinski M., Kmiecik S. (2018). CABS-Flex 2.0: A Web Server for Fast Simulations of Flexibility of Protein Structures. Nucleic Acids Res..

[41] Pires D.E.V., Blundell T.L., Ascher D.B. (2015). pkCSM: Predicting Small-Molecule Pharmacokinetic and Toxicity Properties Using Graph-Based Signatures. J. Med. Chem..

[42] Gore M., Jagtap U.B. (2018). Computational Drug Discovery and Design.

[43] Osorio D., Zhong Y., Li G., Xu Q., Yang Y., Tian Y., Chapkin R.S., Huang J.Z., Cai J.J. (2022). scTenifoldKnk: An Efficient Virtual Knockout Tool for Gene Function Predictions via Single-Cell Gene Regulatory Network Perturbation. Patterns.

[44] Yu G., Wang L.-G., Han Y., He Q.-Y. (2012). clusterProfiler: An R Package for Comparing Biological Themes Among Gene Clusters. OMICS J. Integr. Biol..

[45] Castanza A.S., Recla J.M., Eby D., Thorvaldsdóttir H., Bult C.J., Mesirov J.P. (2023). Extending Support for Mouse Data in the Molecular Signatures Database (MSigDB). Nat. Methods.

[46] Dai H., Xu X., Li W., Fu X., Han W., Li G. (2023). Investigating the Vital Role of the Identified Abietic Acid from *Helianthus annuus* L. Calathide Extract against Hyperuricemia via Human Embryonic Kidney 293T Cell Model. Molecules.

[47] Terkeltaub R. (2009). Gout. Novel Therapies for Treatment of Gout and Hyperuricemia. Arthritis Res. Ther..

[48] Chen Z., Chen R., Wang J., Zhu L., Niu J., Li M., Wu K., Mo J., Zheng S., Liu B. (2025). Ligusticum Cycloprolactam Ameliorates Hyperuricemic Nephropathy through Inhibition of *TLR4*/NF-κB Signaling. J. Nutr. Biochem..

[49] Li M., Chen L.-X., Chen S.-R., Deng Y., Zhao J., Wang Y., Li S.-P. (2017). Non-Starch Polysaccharide from Chinese Yam Activated RAW 264.7 Macrophages through the Toll-like Receptor 4 (*TLR4*)-NF-κB Signaling Pathway. J. Funct. Foods.

[50] Brown J., Wang H., Hajishengallis G.N., Martin M. (2011). TLR-Signaling Networks: An Integration of Adaptor Molecules, Kinases, and Cross-Talk. J. Dent. Res..

[51] Luo Y., Huang P., Chen J., Ma P. (2024). Integrating Network Pharmacology and Experimental Models to Investigate the Mechanisms of XCHD and YCSLS in Preventing HUA Progression via *TLR4*/MYD88/NF-κB Signaling. Heliyon.

[52] Aleksander S.A., Balhoff J., Carbon S., Cherry J.M., Drabkin H.J., Ebert D., Feuermann M., Gaudet P., Harris N.L., The Gene Ontology Consortium (2023). The Gene Ontology Knowledgebase in 2023. Genetics.

[53] Kanehisa M., Furumichi M., Sato Y., Matsuura Y., Ishiguro-Watanabe M. (2025). KEGG: Biological Systems Database as a Model of the Real World. Nucleic Acids Res..

[54] McInnes L., Healy J., Saul N., Großberger L. (2018). UMAP: Uniform Manifold Approximation and Projection. J. Open Source Softw..

[55] Yu H., Xue W., Yu H., Song Y., Liu X., Qin L., Wang S., Bao H., Gu H., Chen G. (2023). Single-Cell Transcriptomics Reveals Variations in Monocytes and Tregs between Gout Flare and Remission. JCI Insight.

[56] Chang J.-G., Tu S.-J., Huang C.-M., Chen Y.-C., Chiang H.-S., Lee Y.-T., Yen J.-C., Lin C.-L., Chung C.-C., Liu T.-C. (2022). Single-Cell RNA Sequencing of Immune Cells in Patients with Acute Gout. Sci. Rep..

[57] Cronstein B.N., Terkeltaub R. (2006). The Inflammatory Process of Gout and Its Treatment. Arthritis Res. Ther..

[58] Bowden J., Davey Smith G., Burgess S. (2015). Mendelian Randomization with Invalid Instruments: Effect Estimation and Bias Detection through Egger Regression. Int. J. Epidemiol..

[59] Pan F., Zhang Y., Li M., Liu M. (2025). *IL1A* Regulates MSU-Induced Apoptosis and Inflammatory Response through *TLR4*/MyD88/NF-κB Signaling Pathway. Int. J. Med. Sci..

[60] Arifuzzaman M., Collins N., Guo C.-J., Artis D. (2024). Nutritional Regulation of Microbiota-Derived Metabolites: Implications for Immunity and Inflammation. Immunity.

[61] Zheng D., Liwinski T., Elinav E. (2020). Interaction between Microbiota and Immunity in Health and Disease. Cell Res..

[62] Li H.-B., Xu M.-L., Xu X.-D., Tang Y.-Y., Jiang H.-L., Li L., Xia W.-J., Cui N., Bai J., Dai Z.-M. (2022). Faecalibacterium Prausnitzii Attenuates CKD via Butyrate-Renal GPR43 Axis. Circ. Res..

[63] Adasme M.F., Linnemann K.L., Bolz S.N., Kaiser F., Salentin S., Haupt V.J., Schroeder M. (2021). PLIP 2021: Expanding the Scope of the Protein–Ligand Interaction Profiler to DNA and RNA. Nucleic Acids Res..

[64] Park B.S., Song D.H., Kim H.M., Choi B.-S., Lee H., Lee J.-O. (2009). The Structural Basis of Lipopolysaccharide Recognition by the *TLR4*–MD-2 Complex. Nature.

[65] Ohto U., Fukase K., Miyake K., Satow Y. (2007). Crystal Structures of Human MD-2 and Its Complex with Antiendotoxic Lipid IVa. Science.

[66] Li B., Lu F., Wei X., Zhao R. (2008). fucoidan: Structure and Bioactivity. Molecules.

[67] Tissot B., Montdargent B., Chevolot L., Varenne A., Descroix S., Gareil P., Daniel R. (2003). Interaction of fucoidan with the Proteins of the Complement Classical Pathway. Biochim. Biophys. Acta BBA—Proteins Proteom..

[68] Wang Y., Ren K., Tan J., Mao Y. (2023). Alginate Oligosaccharide Alleviates Aging-Related Intestinal Mucosal Barrier Dysfunction by Blocking FGF1-Mediated *TLR4*/NF-κB P65 Pathway. Phytomedicine.

[69] Wang G., Zhang N., Zhou Y., Yan J., Liu Y., Liu M., Xue M., Liang H. (2025). Inhibiting HSCs Activation and Ameliorating Alcoholic Liver Fibrosis by fucoidan Targeting the *TLR4*/NF-κB Pathway. J. Food Sci..

[70] Yang B., Li W., Saeki H., Shimizu Y., Joe G.-H. (2024). Maillard-Type Glycated Collagen with Alginate Oligosaccharide Suppresses Inflammation and Oxidative Stress by Attenuating the Expression of LPS Receptors *TLR4* and *Cd14* in Macrophages. Food Funct..

[71] Mahler B.P., Nagarajan B., Sankaranarayanan N.V., Joseph P.R.B., Desai U.R., Rajarathnam K. (2026). Structural Basis of Chemokine CXCL8 Monomer and Dimer Binding to Chondroitin Sulfate: Insights into Specificity and Plasticity. Biomolecules.

[72] Fu Y., Kim H., Lee D.S., Han A., Heine H., Zamyatina A., Kim H.M. (2025). Structural Insight into *TLR4*/MD-2 Activation by Synthetic LPS Mimetics with Distinct Binding Modes. Nat. Commun..

[73] Paramo T., Piggot T.J., Bryant C.E., Bond P.J. (2013). The Structural Basis for Endotoxin-Induced Allosteric Regulation of the Toll-like Receptor 4 (*TLR4*) Innate Immune Receptor. J. Biol. Chem..

[74] Tripathi D., Ramar M., Lavudi K., Sharma S., Rajinikanth P.S., Pandey P. (2025). The Potential of fucoidans from Ocean Treasures to Biomedical Marvels: A Review. Int. J. Biol. Macromol..

[75] Hariyadi D.M., Islam N. (2020). Current Status of Alginate in Drug Delivery. Adv. Pharmacol. Pharm. Sci..

[76] Robbens J., Vanparys C., Nobels I., Blust R., Van Hoecke K., Janssen C., De Schamphelaere K., Roland K., Blanchard G., Silvestre F. (2010). Eco-, Geno- and Human Toxicology of Bio-Active Nanoparticles for Biomedical Applications. Toxicology.

[77] Vávra J., Mančíková A., Pavelcová K., Hasíková L., Bohatá J., Stibůrková B. (2022). Functional Characterization of Rare Variants in OAT1/SLC22A6 and OAT3/SLC22A8 Urate Transporters Identified in a Gout and Hyperuricemia Cohort. Cells.

[78] Enomoto A., Kimura H., Chairoungdua A., Shigeta Y., Jutabha P., Ho Cha S., Hosoyamada M., Takeda M., Sekine T., Igarashi T. (2002). Molecular Identification of a Renal Urate–Anion Exchanger That Regulates Blood Urate Levels. Nature.

[79] Xiao J., Zhang X.-L., Fu C., Han R., Chen W., Lu Y., Ye Z. (2015). Soluble Uric Acid Increases NALP3 Inflammasome and Interleukin-1β Expression in Human Primary Renal Proximal Tubule Epithelial Cells through the Toll-like Receptor 4-Mediated Pathway. Int. J. Mol. Med..

[80] Sokol H., Pigneur B., Watterlot L., Lakhdari O., Bermúdez-Humarán L.G., Gratadoux J.-J., Blugeon S., Bridonneau C., Furet J.-P., Corthier G. (2008). *Faecalibacterium prausnitzii* Is an Anti-Inflammatory Commensal Bacterium Identified by Gut Microbiota Analysis of Crohn Disease Patients. Proc. Natl. Acad. Sci. USA.

