# Supplementary material for: Integrative Bioinformatics Prioritizes the TLR4 Axis and Candidate Non-Starch Polysaccharides in Hyperuricemia-Associated Inflammation
Source: Biology (Basel). 2026 Jul 14;15(14):1150. doi: 10.3390/biology15141150 (PMC13406026; doi:10.3390/biology15141150)
Supplement: Supplementary file 1 [file biology-15-01150-s001.zip › biology-4421470-supplementary.pdf]

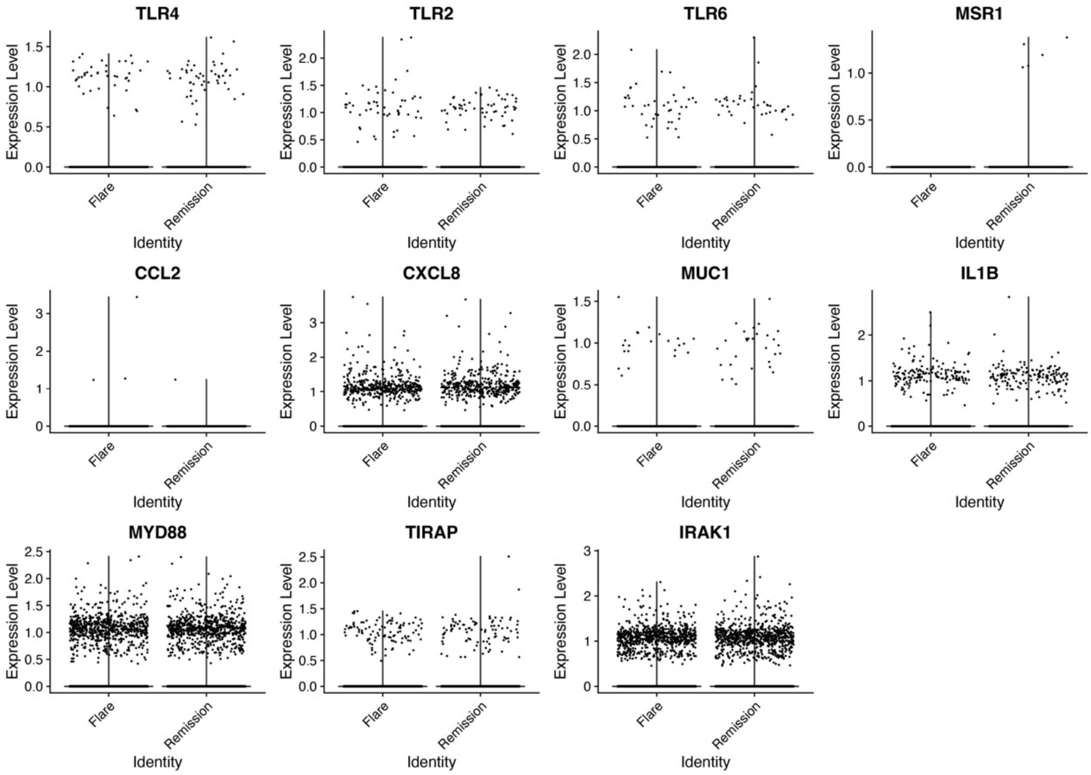

Figure S1. Group-level expression distributions of selected prioritized inflammatory genes in PBMCs from patients with acute gout during flare and remission in the GSE211783 dataset. Jittered expression plots are shown for TLR4, TLR2, TLR6, MSR1, CCL2, CXCL8, MUC1, IL1B, MYD88, TIRAP, and IRAK1, providing supplementary context for the expression patterns of the prioritized TLR4-axis-related genes across the two clinical states.

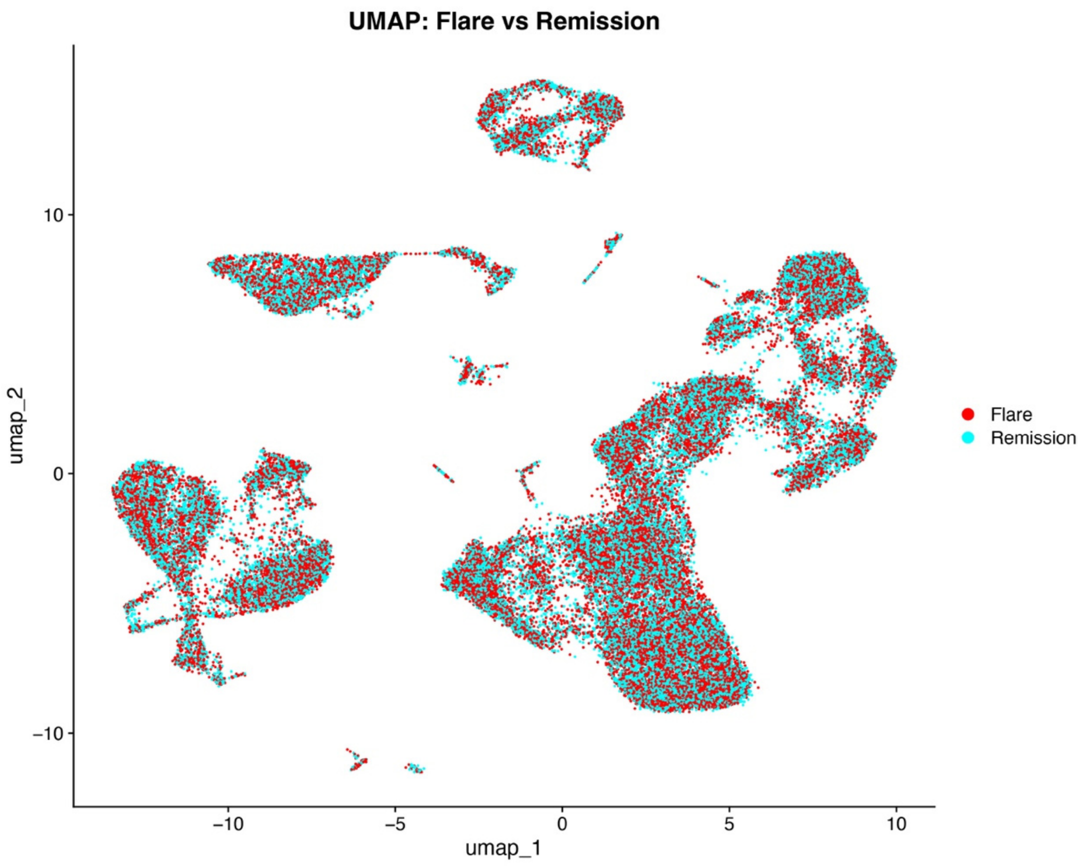

Figure S2. Group-level UMAP distribution of PBMCs from patients sampled during acute gout flare and remission in the GSE211783 dataset. Cells from the two clinical states are broadly intermingled in the low-dimensional space, indicating that global clustering structure is driven predominantly by cell-type identity rather than by overall condition-specific separation.

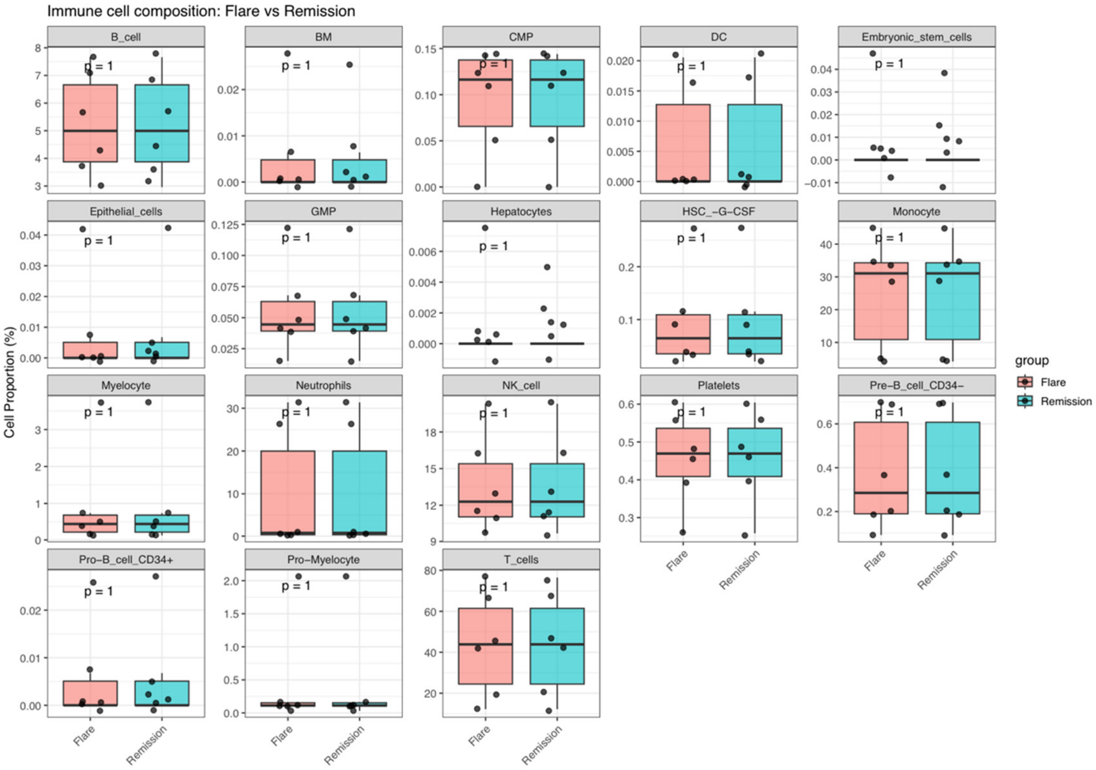

Figure S3. Comparison of overall immune cell-type proportions between flare and remission samples in the GSE211783 PBMC dataset. Boxplots show the estimated proportions of major immune and minor myeloid cell populations, with Wilcoxon test P values annotated in each panel. No statistically significant global differences in overall immune cell composition were observed between the two clinical states.

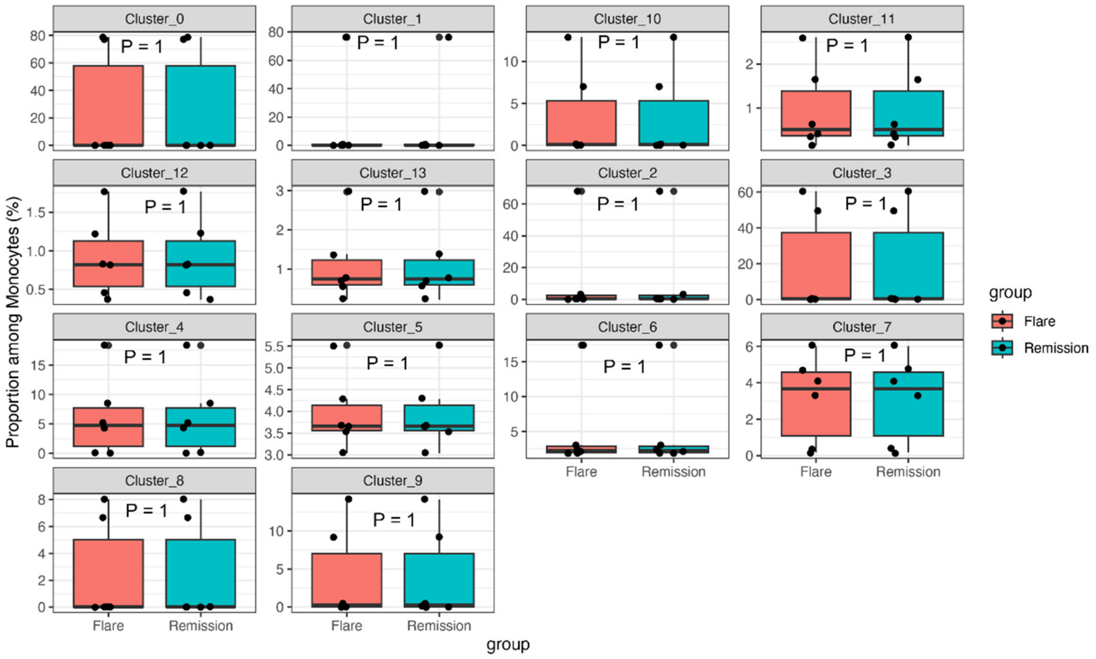

Figure S4. Comparison of monocyte subcluster proportions between flare and remission samples in the GSE211783 PBMC dataset. Boxplots display the relative proportions of individual monocyte subclusters, with Wilcoxon test P values shown in each panel. No statistically significant differences in monocyte subtype composition were observed between flare and remission samples.

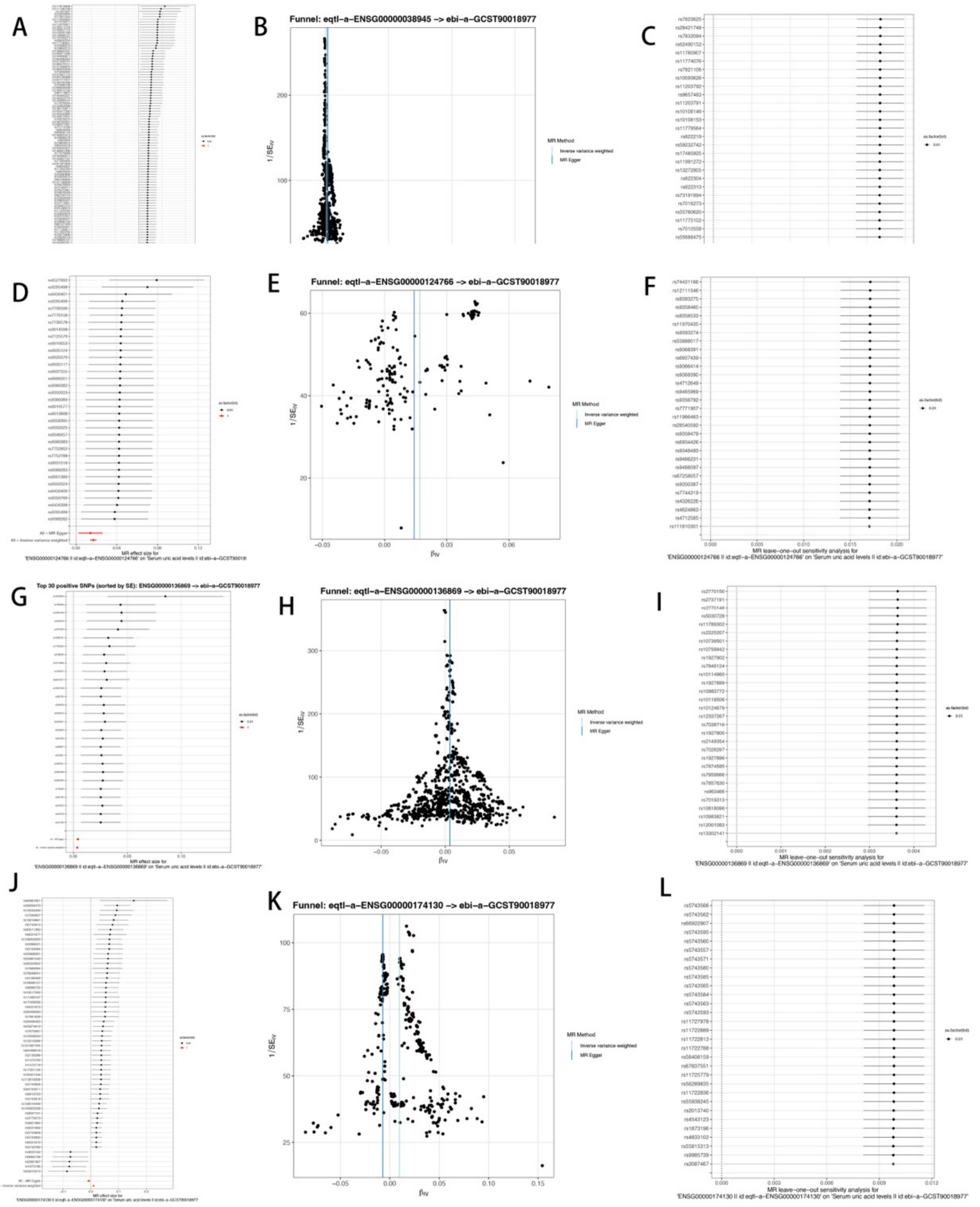

Figure S5. Mendelian randomization analysis evaluating the causal effects of key genes on serum uric acid levels. (A) MSR1 forest plot: MR estimates of individual SNPs and overall effect. (B) MSR1 funnel plot: assessment of directional pleiotropy. (C) MSR1 leave-one-out sensitivity analysis plot: showing the stability of causal estimates after removing individual SNPs one by one. (D) CXCL8 forest plot: MR estimates of individual SNPs and overall effect. (E) CXCL8 funnel plot: assessment of directional pleiotropy. (F) CXCL8 leave-one-out sensitivity analysis plot: showing the stability of causal estimates after removing individual SNPs one by one. (G) TLR4 forest plot: MR estimates of individual SNPs and overall effect. (H) TLR4 funnel plot: assessment of directional pleiotropy. (I) TLR4 leave-one-out sensitivity analysis plot: showing the stability of causal estimates after removing individual SNPs one by one. (J) TIRAP forest plot: MR estimates of individual SNPs and overall effect. (K) TIRAP funnel plot: assessing directional pleiotropy. (L) TIRAP leave-one-out sensitivity analysis plot: showing the stability of causal estimates after removing individual SNPs one by one.

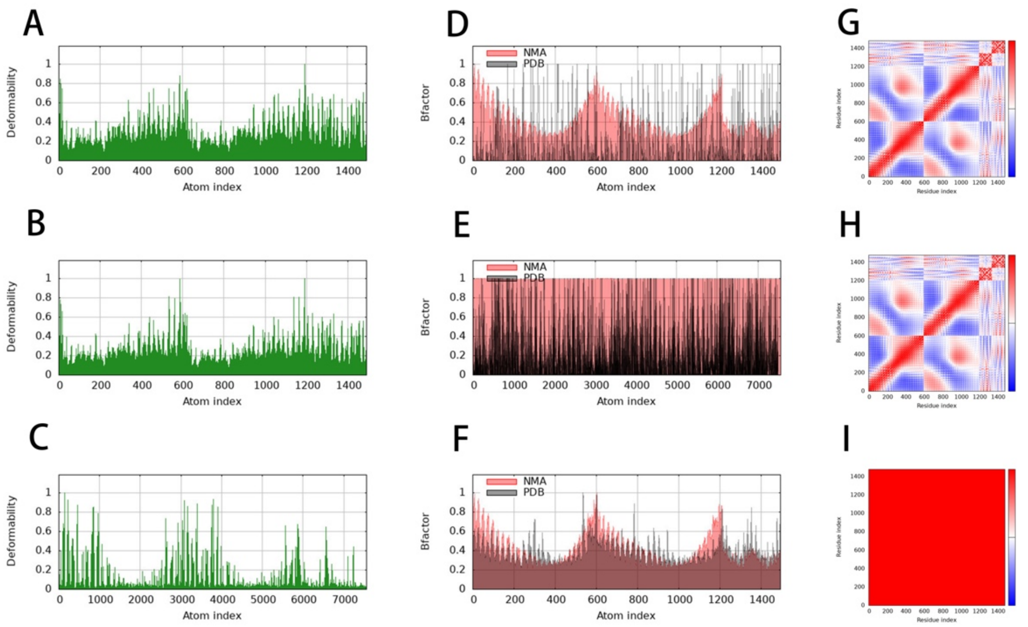

Figure S6. (A) Deformability plot of the unbound TLR4 (3FXI) structure, atom index range 0-1400.(B) Deformability plot of the Alginate-TLR4 complex, atom index range 0-1400.(C) Deformability plot of the Fucoidan-TLR4 complex, atom index range of 0-7000.(D) B-factor/Mobility comparison plot of the alginate–TLR4 complex, with NMA-calculated values (red) and experimental PDB values (black). (E) B-factor/Mobility comparison plot of the fucoidan–TLR4 complex. (F) B-factor/Mobility comparison plot of the unbound TLR4 structure (3FXI). (G) Covariance matrix of the unbound TLR4 structure (3FXI), showing anti-correlated motion regions (blue). (H) Covariance matrix of the alginate–TLR4 complex, showing anti-correlated motion regions (blue). (I) Covariance matrix of the fucoidan–TLR4 complex, showing predominantly positive-correlation pattern (red).

Table S1. Complete Gene Ontology biological process (GO-BP) enrichment results for the prioritized candidate gene set, including term names, gene counts, adjusted P-values, fold enrichment, and associated genes.

Category	Term	Count	%	PValue	Genes	List Total	Pop Hits	Pop Total	Fold Enrichment	Bonferroni	Benjamini	FDR
GOTERM_BP_DIRECT	GO:0002224~toll-like receptor signaling pathway	9	45	3.2353e-19	TLR1, IRAK1, LY96, TLR6, IRAK4, TLR5, TLR4, TLR3, TLR2	20	27	19478	324.633	1.2488e-16	1.2488e-16	9.7382e-17
GOTERM_BP_DIRECT	GO:0034142~toll-like receptor 4 signaling pathway	9	45	8.5149e-19	IRAK1, IRF3, IRAK2, TRAF6, LY96, IRAK4, TLR4, TIRAP, MYD88	20	30	19478	292.17	3.2867e-16	1.6434e-16	1.2815e-16
GOTERM_BP_DIRECT	GO:0002755~MyD88-dependent toll-like receptor signaling pathway	8	40	2.7191e-18	IRAK1, IRAK2, TRAF6, TLR6, IRAK4, TLR4, TIRAP, MYD88	20	16	19478	486.95	1.0496e-15	3.4986e-16	2.7282e-16
GOTERM_BP_DIRECT	GO:0031663~lipopolysaccharide-mediated signaling pathway	9	45	4.3874e-18	IRAK1, IRF3, IRAK2, TRAF6, LY96, IRAK4, TLR4, MYD88, TLR2	20	36	19478	243.475	1.6935e-15	4.2338e-16	3.3015e-16
GOTERM	GO:0006954~inflammatory	14	70	6.2595e-18	CXCL8, LY96, TIRAP, TLR1, IL1A,	20	432	19478	31.5616	2.4162e-15	4.8323e-16	3.7682e-16

Category	Term	Count	%	PValue	Genes	List Total	Pop Hits	Pop Total	Fold Enrichment	Bonferroni	Benjamini	FDR
_BP_DIRECT	response				IRAK2, IL1B, CCL2, TLR6, TLR5, TLR4, MYD88, TLR3, TLR2							
GOTERM_BP_DIRECT	GO:0043123~positive regulation of canonical NF-kappaB signal transduction	12	60	3.7222e-17	IL1A, IRAK1, IRF3, IRAK2, IL1B, TRAF6, TLR6, IRAK4, TLR4, TIRAP, MYD88, TLR3	20	232	19478	50.3741	1.4368e-14	2.3946e-15	1.8673e-15
GOTERM_BP_DIRECT	GO:0032755~positive regulation of interleukin-6 production	10	50	1.8170e-16	TLR1, IL1A, IL1B, TRAF6, TLR6, TLR4, TIRAP, MYD88, TLR3, TLR2	20	102	19478	95.4804	8.5709e-14	1.0019e-14	7.8130e-15
GOTERM_BP_DIRECT	GO:0045087~innate immune response	13	65	1.4700e-14	LY96, IRAK4, TIRAP, TLR1, IRAK1, IRF3, TRAF6, TLR6, TLR5, TLR4, MYD88, TLR3, TLR2	20	570	19478	22.2118	5.6568e-12	7.0927e-13	5.5308e-13
GOTERM_BP_DIRECT	GO:0032757~positive regulation of interleukin-8 production	8	40	1.6132e-13	TLR1, IL1B, TLR5, TLR4, TIRAP, MYD88, TLR3, TLR2	20	65	19478	119.865	6.2268e-11	6.9189e-12	5.3953e-12
GOTERM_BP_DIRECT	GO:0071222~cellular response to lipopolysaccharide	9	45	5.3044e-12	IL1A, CXCL8, IL1B, TRAF6, CCL2, LY96, TLR5, TLR4, MYD88	20	192	19478	45.6516	2.0475e-09	2.0475e-10	1.5966e-10
GOTERM_BP_DIRECT	GO:0032760~positive regulation of tumor necrosis factor production	8	40	8.1812e-12	TLR1, IL1A, LY96, TLR4, TIRAP, MYD88, TLR3, TLR2	20	112	19478	69.5643	3.1579e-09	2.8708e-10	2.2387e-10
GOTERM_BP_DIRECT	GO:0051092~positive regulation of NF-kappaB transcription factor activity	8	40	1.3376e-11	IRAK1, IRAK2, IL1B, TRAF6, TLR4, TIRAP, MYD88, TLR2	20	120	19478	64.9267	5.1630e-09	4.3025e-10	3.3551e-10
GOTERM_BP_DIRECT	GO:0070498~interleukin-1-mediated signaling pathway	6	30	1.6576e-11	IRAK1, IRAK2, IL1B, TRAF6, IRAK4, MYD88	20	23	19478	254.061	6.3982e-09	4.9217e-10	3.8379e-10
GOTERM_BP_DIRECT	GO:0007165~signal transduction	12	60	5.5006e-09	TLR1, CXCL8, IRAK1, IRAK2, IL1B, CCL2, TLR6, IRAK4, TLR4, MYD88, TLR3, TLR2	20	1308	19478	8.93486	2.1232e-06	1.5166e-07	1.1826e-07
GOTERM_BP_DIRECT	GO:0010628~positive regulation of gene expression	9	45	8.8352e-09	IL1A, CXCL8, IL1B, CCL2, TLR6, TLR4, MYD88, TLR3, TLR2	20	489	19478	17.9245	3.4104e-06	2.2736e-07	1.7729e-07
GOTERM_BP_DIRECT	GO:0007254~JNK cascade	6	30	1.2190e-08	IRAK1, IL1B, IRAK4, TLR4, MYD88, TLR3	20	81	19478	72.1407	4.7052e-06	2.9408e-07	2.2932e-07
GOTERM_BP_DIRECT	GO:1900017~positive regulation of cytokine production involved in inflammatory response	5	25	2.0006e-08	IRF3, TLR6, TLR4, MYD88, TLR3	20	31	19478	157.081	7.7221e-06	4.5424e-07	3.5422e-07
GOTERM_BP_DIRECT	GO:0008063~Toll signaling pathway	4	20	2.7473e-08	IRAK1, IRAK2, IRAK4, MYD88	20	7	19478	556.514	1.0605e-05	5.8914e-07	4.5941e-07
GOTERM	GO:0046330~positive	6	30	3.3662e-08	IL1A, IL1B, TLR4, TIRAP, MYD88,	20	99	19478	59.0242	1.2993e-05	6.8387e-07	5.3327e-07

Category	Term	Count	%	PValue	Genes	List Total	Pop Hits	Pop Total	Fold Enrichment	Bonferroni	Benjamini	FDR
_BP_DIRECT	regulation of JNK cascade				TLR3							
GOTERM_BP_DIRECT	GO:0070555~response to interleukin-1	5	25	3.7337e-08	IRAK1, IRAK2, IL1B, TRAF6, MYD88	20	36	19478	135.264	1.4412e-05	7.2060e-07	5.6192e-07
GOTERM_BP_DIRECT	GO:0038172~interleukin-33-mediated signaling pathway	4	20	4.3930e-08	IRAK1, TRAF6, IRAK4, MYD88	20	8	19478	486.95	1.6957e-05	8.0747e-07	6.2966e-07
GOTERM_BP_DIRECT	GO:0032735~positive regulation of interleukin-12 production	5	25	9.3917e-08	TRAF6, TLR4, TIRAP, TLR3, TLR2	20	45	19478	108.211	3.6251e-05	1.6478e-06	1.2850e-06
GOTERM_BP_DIRECT	GO:0035666~TRIF-dependent toll-like receptor signaling pathway	4	20	1.2920e-07	IRF3, TRAF6, TLR6, TLR4	20	11	19478	354.145	4.9869e-05	2.1683e-06	1.6908e-06
GOTERM_BP_DIRECT	GO:0032496~response to lipopolysaccharide	6	30	1.9146e-07	IRAK1, IL1B, LY96, TLR4, TIRAP, TLR2	20	140	19478	41.7386	7.3902e-05	3.0794e-06	2.4013e-06
GOTERM_BP_DIRECT	GO:0006955~immune response	8	40	4.3991e-07	TLR1, IL1A, CXCL8, IL1B, CCL2, TLR6, TLR4, TLR2	20	537	19478	14.5088	0.000169792	6.7923e-06	5.2966e-06
GOTERM_BP_DIRECT	GO:0071260~cellular response to mechanical stimulus	5	25	9.7563e-07	IL1B, TLR5, TLR4, MYD88, TLR3	20	80	19478	60.8687	0.000376522	1.4484e-05	1.1295e-05
GOTERM_BP_DIRECT	GO:0001774~microglial cell activation	4	20	2.8296e-06	TLR1, TLR6, TLR3, TLR2	20	29	19478	134.331	0.00109161	4.0452e-05	3.1544e-05
GOTERM_BP_DIRECT	GO:0038124~toll-like receptor TLR6:TLR2 signaling pathway	3	15	5.4026e-06	TLR6, MYD88, TLR2	20	4	19478	730.425	0.00208325	7.4479e-05	5.8078e-05
GOTERM_BP_DIRECT	GO:0032481~positive regulation of type I interferon production	4	20	7.0335e-06	IRAK1, IRF3, TRAF6, MYD88	20	39	19478	99.8872	0.00271125	9.3618e-05	7.3003e-05
GOTERM_BP_DIRECT	GO:0045944~positive regulation of transcription by RNA polymerase II	9	45	8.5696e-06	IL1A, MUC1, IRF3, IL1B, TRAF6, TLR4, MYD88, TLR3, TLR2	20	1205	19478	7.27394	0.00330243	0.000110263	8.5982e-05
GOTERM_BP_DIRECT	GO:0032728~positive regulation of interferon-beta production	4	20	9.4744e-06	IRF3, TLR4, TLR3, TLR2	20	43	19478	90.5953	0.00365046	0.000117972	9.1993e-05
GOTERM_BP_DIRECT	GO:0032722~positive regulation of chemokine production	4	20	1.0880e-05	TLR4, MYD88, TLR3, TLR2	20	45	19478	86.5689	0.00419109	0.000131246	0.000102345
GOTERM_BP_DIRE	GO:0045429~positive regulation of nitric oxide	4	20	1.2418e-05	IL1B, TLR6, TLR5, TLR4	20	47	19478	82.8851	0.00478186	0.000145252	0.000113266

Category	Term	Count	%	PValue	Genes	List Total	Pop Hits	Pop Total	Fold Enrichment	Bonferroni	Benjamini	FDR
CT	biosynthetic process											
GOTERM_BP_DIRECT	GO:0007252~I-kappaB phosphorylation	3	15	1.8876e-05	TLR4, TLR3, TLR2	20	7	19478	417.386	0.00725981	0.000208592	0.000162659
GOTERM_BP_DIRECT	GO:0048661~positive regulation of smooth muscle cell proliferation	4	20	1.8914e-05	IRAK1, IRAK4, TLR4, MYD88	20	54	19478	72.1407	0.00727421	0.000208592	0.000162659
GOTERM_BP_DIRECT	GO:1901224~positive regulation of non-canonical NF-kappaB signal transduction	4	20	3.6237e-05	IL1B, TLR4, TLR3, TLR2	20	67	19478	58.1433	0.0138905	0.000388546	0.000302986
GOTERM_BP_DIRECT	GO:0071223~cellular response to lipoteichoic acid	3	15	4.9323e-05	TLR4, TIRAP, TLR2	20	11	19478	265.609	0.018859	0.000514557	0.000401247
GOTERM_BP_DIRECT	GO:0071346~cellular response to type II interferon	4	20	5.7158e-05	CCL2, TLR4, TLR3, TLR2	20	78	19478	49.9436	0.0218222	0.00058061	0.000452755
GOTERM_BP_DIRECT	GO:0140052~cellular response to oxidised low-density lipoprotein particle stimulus	3	15	8.1464e-05	TLR6, TLR4, MYD88	20	14	19478	208.693	0.0309573	0.000806289	0.000628738
GOTERM_BP_DIRECT	GO:0050729~positive regulation of inflammatory response	4	20	0.000167693	IL1B, TLR4, TLR3, TLR2	20	112	19478	34.7821	0.062684	0.00161824	0.00126189
GOTERM_BP_DIRECT	GO:0060907~positive regulation of macrophage cytokine production	3	15	0.000266851	TLR4, MYD88, TLR3	20	25	19478	116.868	0.0978895	0.0025123	0.00195908
GOTERM_BP_DIRECT	GO:0032727~positive regulation of interferon-alpha production	3	15	0.000311853	IRF3, TLR4, TLR3	20	27	19478	108.211	0.113429	0.00279942	0.00218297
GOTERM_BP_DIRECT	GO:1900227~positive regulation of NLRP3 inflammasome complex assembly	3	15	0.000311853	TLR6, TLR4, MYD88	20	27	19478	108.211	0.113429	0.00279942	0.00218297
GOTERM_BP_DIRECT	GO:0050830~defense response to Gram-positive bacterium	4	20	0.000323674	IL1B, TIRAP, MYD88, TLR2	20	140	19478	27.8257	0.117466	0.00283951	0.00221423
GOTERM_BP_DIRECT	GO:0019221~cytokine-mediated signaling pathway	4	20	0.000337432	IL1A, IL1B, CCL2, IRAK4	20	142	19478	27.4338	0.122142	0.00289442	0.00225705
GOTERM_BP_DIRECT	GO:0045766~positive regulation of angiogenesis	4	20	0.000487314	IL1A, CXCL8, IL1B, TLR3	20	161	19478	24.1963	0.171509	0.0040892	0.00318873

Category	Term	Count	%	PValue	Genes	List Total	Pop Hits	Pop Total	Fold Enrichment	Bonferroni	Benjamini	FDR
GOTERM_BP_DIRECT	GO:0042742~defense response to bacterium	4	20	0.000580926	TLR6, TLR4, MYD88, TLR3	20	171	19478	22.7813	0.200927	0.00473083	0.00368907
GOTERM_BP_DIRECT	GO:0032743~positive regulation of interleukin-2 production	3	15	0.00058829	IL1A, IL1B, TRAF6	20	37	19478	78.9649	0.203196	0.00473083	0.00368907
GOTERM_BP_DIRECT	GO:0007249~canonical NF-kappaB signal transduction	3	15	0.000620612	IRAK1, IRAK2, TRAF6	20	38	19478	76.8868	0.213081	0.0048889	0.00381233
GOTERM_BP_DIRECT	GO:0070374~positive regulation of ERK1 and ERK2 cascade	4	20	0.00101099	IL1A, IL1B, TLR4, TIRAP	20	207	19478	18.8193	0.32324	0.00780482	0.00608614
GOTERM_BP_DIRECT	GO:0060337~type I interferon-mediated signaling pathway	3	15	0.00107392	IRAK1, IRF3, MYD88	20	50	19478	58.434	0.339498	0.00812809	0.00633823
GOTERM_BP_DIRECT	GO:0051607~defense response to virus	4	20	0.0015656	IRF3, MYD88, TLR3, TLR2	20	241	19478	16.1643	0.453814	0.0116216	0.00906243
GOTERM_BP_DIRECT	GO:0032731~positive regulation of interleukin-1 beta production	3	15	0.00186306	TLR6, TLR4, MYD88	20	66	19478	44.2682	0.513155	0.0129777	0.0101199
GOTERM_BP_DIRECT	GO:0034146~toll-like receptor 5 signaling pathway	2	10	0.00195002	TLR5, MYD88	20	2	19478	973.9	0.529256	0.0129777	0.0101199
GOTERM_BP_DIRECT	GO:0042495~detection of triacyl bacterial lipopeptide	2	10	0.00195002	TLR1, TLR2	20	2	19478	973.9	0.529256	0.0129777	0.0101199
GOTERM_BP_DIRECT	GO:0032493~response to bacterial lipoprotein	2	10	0.00195002	TLR6, TLR2	20	2	19478	973.9	0.529256	0.0129777	0.0101199
GOTERM_BP_DIRECT	GO:1903974~positive regulation of cellular response to macrophage colony-stimulating factor stimulus	2	10	0.00195002	TLR4, TLR2	20	2	19478	973.9	0.529256	0.0129777	0.0101199
GOTERM_BP_DIRECT	GO:0042496~detection of diacyl bacterial lipopeptide	2	10	0.00195002	TLR6, TLR2	20	2	19478	973.9	0.529256	0.0129777	0.0101199
GOTERM_BP_DIRECT	GO:0032729~positive regulation of type II interferon production	3	15	0.00259017	IL1B, TLR4, TLR3	20	78	19478	37.4577	0.632525	0.0169458	0.0132142
GOTERM_BP_DIRECT	GO:1904466~positive regulation of matrix metallopeptidase secretion	2	10	0.00292367	TLR4, TLR2	20	3	19478	649.267	0.677029	0.0176334	0.0137504

Category	Term	Count	%	PValue	Genes	List Total	Pop Hits	Pop Total	Fold Enrichment	Bonferroni	Benjamini	FDR
GOTERM_BP_DIRECT	GO:0001660~fever generation	2	10	0.00292367	IL1A, IL1B	20	3	19478	649.267	0.677029	0.0176334	0.0137504
GOTERM_BP_DIRECT	GO:0002730~regulation of dendritic cell cytokine production	2	10	0.00292367	TLR4, TLR3	20	3	19478	649.267	0.677029	0.0176334	0.0137504
GOTERM_BP_DIRECT	GO:0071727~cellular response to triacyl bacterial lipopeptide	2	10	0.00292367	TLR1, TLR2	20	3	19478	649.267	0.677029	0.0176334	0.0137504
GOTERM_BP_DIRECT	GO:0001959~regulation of cytokine-mediated signaling pathway	2	10	0.00292367	IRAK1, IRAK2	20	3	19478	649.267	0.677029	0.0176334	0.0137504
GOTERM_BP_DIRECT	GO:0006915~apoptotic process	5	25	0.00330017	IL1A, IRF3, IL1B, MYD88, TLR2	20	656	19478	7.42302	0.72084	0.0195979	0.0152823
GOTERM_BP_DIRECT	GO:0007166~cell surface receptor signaling pathway	4	20	0.0043946	CCL2, LY96, TIRAP, MYD88	20	347	19478	11.2265	0.817326	0.0257017	0.020042
GOTERM_BP_DIRECT	GO:0032497~detection of lipopolysaccharide	2	10	0.00486829	LY96, TLR4	20	5	19478	389.56	0.847981	0.0276347	0.0215493
GOTERM_BP_DIRECT	GO:0071726~cellular response to diacyl bacterial lipopeptide	2	10	0.00486829	TLR6, TLR2	20	5	19478	389.56	0.847981	0.0276347	0.0215493
GOTERM_BP_DIRECT	GO:0034134~toll-like receptor 2 signaling pathway	2	10	0.00583925	IRAK1, TLR2	20	6	19478	324.633	0.895708	0.0313049	0.0244113
GOTERM_BP_DIRECT	GO:0034137~positive regulation of toll-like receptor 2 signaling pathway	2	10	0.00583925	TLR1, TIRAP	20	6	19478	324.633	0.895708	0.0313049	0.0244113
GOTERM_BP_DIRECT	GO:0071221~cellular response to bacterial lipopeptide	2	10	0.00583925	TIRAP, TLR2	20	6	19478	324.633	0.895708	0.0313049	0.0244113
GOTERM_BP_DIRECT	GO:0033092~positive regulation of immature T cell proliferation in thymus	2	10	0.00583925	IL1A, IL1B	20	6	19478	324.633	0.895708	0.0313049	0.0244113
GOTERM_BP_DIRECT	GO:0008285~negative regulation of cell population proliferation	4	20	0.00811226	IL1A, CXCL8, IL1B, TLR2	20	433	19478	8.99677	0.956894	0.042895	0.0334492
GOTERM_BP_DIRECT	GO:0042088~T-helper 1 type immune response	2	10	0.00874675	TRAF6, TLR4	20	9	19478	216.422	0.966328	0.0450166	0.0351036
GOTERM	GO:0034162~toll-like	2	10	0.00874675	IRAK1, IRAK4	20	9	19478	216.422	0.966328	0.0450166	0.0351036

Category	Term	Count	%	PValue	Genes	List Total	Pop Hits	Pop Total	Fold Enrichment	Bonferroni	Benjamini	FDR
_BP_DIRECT	receptor 9 signaling pathway											
GOTERM_BP_DIRECT	GO:0007250~activation of NF-kappaB-inducing kinase activity	2	10	0.00971412	TLR6, TLR3	20	10	19478	194.78	0.976901	0.0493375	0.038473
GOTERM_BP_DIRECT	GO:0035556~intracellular signal transduction	4	20	0.0101465	CXCL8, IRAK1, IRAK2, IRAK4	20	470	19478	8.28851	0.980485	0.0508643	0.0396636
GOTERM_BP_DIRECT	GO:0014004~microglia differentiation	2	10	0.0106806	TLR4, MYD88	20	11	19478	177.073	0.984155	0.0521862	0.0406944
GOTERM_BP_DIRECT	GO:0031666~positive regulation of lipopolysaccharide-mediated signaling pathway	2	10	0.0106806	TRAF6, LY96	20	11	19478	177.073	0.984155	0.0521862	0.0406944
GOTERM_BP_DIRECT	GO:0032308~positive regulation of prostaglandin secretion	2	10	0.0116462	IL1A, IL1B	20	12	19478	162.317	0.989131	0.0554991	0.0432778
GOTERM_BP_DIRECT	GO:2000343~positive regulation of chemokine (C-X-C motif) ligand 2 production	2	10	0.0116462	TLR4, TIRAP	20	12	19478	162.317	0.989131	0.0554991	0.0432778
GOTERM_BP_DIRECT	GO:0034138~toll-like receptor 3 signaling pathway	2	10	0.0135747	TRAF6, TLR3	20	14	19478	139.129	0.994886	0.0631305	0.0492287
GOTERM_BP_DIRECT	GO:1904996~positive regulation of leukocyte adhesion to vascular endothelial cell	2	10	0.0135747	IRAK1, TRAF6	20	14	19478	139.129	0.994886	0.0631305	0.0492287
GOTERM_BP_DIRECT	GO:0046209~nitric oxide metabolic process	2	10	0.0145376	TLR6, TLR2	20	15	19478	129.853	0.996492	0.0668037	0.052093
GOTERM_BP_DIRECT	GO:1903428~positive regulation of reactive oxygen species biosynthetic process	2	10	0.0164607	TLR6, TLR4	20	17	19478	114.576	0.99835	0.0747511	0.0582904
GOTERM_BP_DIRECT	GO:0035234~ectopic germ cell programmed cell death	2	10	0.017421	IL1A, IL1B	20	18	19478	108.211	0.998868	0.0772931	0.0602726
GOTERM_BP_DIRECT	GO:0071360~cellular response to exogenous dsRNA	2	10	0.017421	IRF3, TLR3	20	18	19478	108.211	0.998868	0.0772931	0.0602726
GOTERM	GO:0002753~cytoplasmic	2	10	0.0183803	IRF3, TRAF6	20	19	19478	102.516	0.999224	0.0806228	0.0628691

Category	Term	Count	%	PValue	Genes	List Total	Pop Hits	Pop Total	Fold Enrichment	Bonferroni	Benjamini	FDR
_BP_DIRECT	pattern recognition receptor signaling pathway											
GOTERM_BP_DIRECT	GO:0043032~positive regulation of macrophage activation	2	10	0.0193388	TLR6, TLR4	20	20	19478	97.39	0.999467	0.0838739	0.0654043
GOTERM_BP_DIRECT	GO:0070935~3'-UTR-mediated mRNA stabilization	2	10	0.0212531	TIRAP, MYD88	20	22	19478	88.5364	0.99975	0.0882117	0.0687868
GOTERM_BP_DIRECT	GO:0032874~positive regulation of stress-activated MAPK cascade	2	10	0.0212531	IL1A, TLR4	20	22	19478	88.5364	0.99975	0.0882117	0.0687868
GOTERM_BP_DIRECT	GO:0042116~macrophage activation	2	10	0.0212531	TLR1, TLR4	20	22	19478	88.5364	0.99975	0.0882117	0.0687868
GOTERM_BP_DIRECT	GO:0010744~positive regulation of macrophage derived foam cell differentiation	2	10	0.0212531	MSR1, IL1B	20	22	19478	88.5364	0.99975	0.0882117	0.0687868
GOTERM_BP_DIRECT	GO:0043507~positive regulation of JUN kinase activity	2	10	0.025071	TRAF6, TLR6	20	26	19478	74.9154	0.999945	0.102951	0.0802805
GOTERM_BP_DIRECT	GO:0090023~positive regulation of neutrophil chemotaxis	2	10	0.0269747	CXCL8, TIRAP	20	28	19478	69.5643	0.999974	0.109602	0.0854671
GOTERM_BP_DIRECT	GO:0045840~positive regulation of mitotic nuclear division	2	10	0.0288748	IL1A, IL1B	20	30	19478	64.9267	0.999988	0.116101	0.0905346
GOTERM_BP_DIRECT	GO:0070372~regulation of ERK1 and ERK2 cascade	2	10	0.0298236	IL1A, IL1B	20	31	19478	62.8323	0.999992	0.118679	0.0925453
GOTERM_BP_DIRECT	GO:0097192~extrinsic apoptotic signaling pathway in absence of ligand	2	10	0.0307714	IL1A, IL1B	20	32	19478	60.8687	0.999994	0.119978	0.0935576
GOTERM_BP_DIRECT	GO:0010575~positive regulation of vascular endothelial growth factor production	2	10	0.0307714	IL1A, IL1B	20	32	19478	60.8687	0.999994	0.119978	0.0935576
GOTERM_BP_DIRECT	GO:0071345~cellular response to cytokine stimulus	2	10	0.0317184	IRAK1, TRAF6	20	33	19478	59.0242	0.999996	0.120033	0.0936005
GOTERM_BP_DIRECT	GO:0045671~negative regulation of osteoclast differentiation	2	10	0.0317184	TLR4, TLR3	20	33	19478	59.0242	0.999996	0.120033	0.0936005

Category	Term	Count	%	PValue	Genes	List Total	Pop Hits	Pop Total	Fold Enrichment	Bonferroni	Benjamini	FDR
GOTERM_BP_DIRECT	GO:0044344~cellular response to fibroblast growth factor stimulus	2	10	0.0317184	CXCL8, CCL2	20	33	19478	59.0242	0.999996	0.120033	0.0936005
GOTERM_BP_DIRECT	GO:0043122~regulation of canonical NF-kappaB signal transduction	2	10	0.0345542	IL1B, TRAF6	20	36	19478	54.1056	0.999999	0.129494	0.100979
GOTERM_BP_DIRECT	GO:2001240~negative regulation of extrinsic apoptotic signaling pathway in absence of ligand	2	10	0.0354977	IL1A, IL1B	20	37	19478	52.6432	0.999999	0.131751	0.102738
GOTERM_BP_DIRECT	GO:2001238~positive regulation of extrinsic apoptotic signaling pathway	2	10	0.0364403	TLR6, TLR4	20	38	19478	51.2579	0.999999	0.133962	0.104462
GOTERM_BP_DIRECT	GO:0032733~positive regulation of interleukin-10 production	2	10	0.0402021	TLR4, TLR2	20	42	19478	46.3762	1	0.146396	0.114159
GOTERM_BP_DIRECT	GO:0043406~positive regulation of MAP kinase activity	2	10	0.0430143	IL1B, TLR4	20	45	19478	43.2844	1	0.155173	0.121003
GOTERM_BP_DIRECT	GO:1904646~cellular response to amyloid-beta	2	10	0.0448847	TLR6, TLR4	20	47	19478	41.4426	1	0.15895	0.123948
GOTERM_BP_DIRECT	GO:0030890~positive regulation of B cell proliferation	2	10	0.0448847	TLR4, TIRAP	20	47	19478	41.4426	1	0.15895	0.123948
GOTERM_BP_DIRECT	GO:0051781~positive regulation of cell division	2	10	0.0467517	IL1A, IL1B	20	49	19478	39.751	1	0.164056	0.12793
GOTERM_BP_DIRECT	GO:0071347~cellular response to interleukin-1	2	10	0.052332	CXCL8, CCL2	20	55	19478	35.4145	1	0.181983	0.141909
GOTERM_BP_DIRECT	GO:0034605~cellular response to heat	2	10	0.053259	IL1A, IRAK1	20	56	19478	34.7821	1	0.183553	0.143134
GOTERM_BP_DIRECT	GO:0030593~neutrophil chemotaxis	2	10	0.056035	CXCL8, IL1B	20	59	19478	33.0136	1	0.191411	0.149261
GOTERM_BP_DIRECT	GO:0042102~positive regulation of T cell proliferation	2	10	0.0597242	IL1B, TRAF6	20	63	19478	30.9175	1	0.202224	0.157693
GOTERM_BP_DIRECT	GO:0140374~antiviral innate immune response	2	10	0.0615637	IRF3, TRAF6	20	65	19478	29.9662	1	0.20664	0.161136
GOTERM	GO:0070098~chemokine-	2	10	0.0661476	CXCL8, CCL2	20	70	19478	27.8257	1	0.220112	0.171642

Category	Term	Count	%	PValue	Genes	List Total	Pop Hits	Pop Total	Fold Enrichment	Bonferroni	Benjamini	FDR
_BP_DIRECT	mediated signaling pathway											
GOTERM_BP_DIRECT	GO:0006909~phagocytosis	2	10	0.0679752	TLR4, MYD88	20	72	19478	27.0528	1	0.222359	0.173394
GOTERM_BP_DIRECT	GO:0071466~cellular response to xenobiotic stimulus	2	10	0.0679752	IL1B, TLR3	20	72	19478	27.0528	1	0.222359	0.173394
GOTERM_BP_DIRECT	GO:0032088~negative regulation of NF-kappaB transcription factor activity	2	10	0.0716202	IRAK1, IRAK2	20	76	19478	25.6289	1	0.232314	0.181157
GOTERM_BP_DIRECT	GO:0043124~negative regulation of canonical NF-kappaB signal transduction	2	10	0.0743451	IRAK1, IRAK2	20	79	19478	24.6557	1	0.239143	0.186482
GOTERM_BP_DIRECT	GO:0051091~positive regulation of DNA-binding transcription factor activity	2	10	0.0806738	IL1B, TRAF6	20	86	19478	22.6488	1	0.257356	0.200684
GOTERM_BP_DIRECT	GO:0098586~cellular response to virus	2	10	0.0896436	IRF3, TLR3	20	96	19478	20.2896	1	0.283627	0.22117
GOTERM_BP_DIRECT	GO:0050727~regulation of inflammatory response	2	10	0.0940974	IRF3, MYD88	20	101	19478	19.2851	1	0.295298	0.230271

Table S2. Complete Gene Ontology cellular component (GO-CC) enrichment results for the prioritized candidate gene set, including term names, gene counts, adjusted P-values, fold enrichment, and associated genes.

Category	Term	Count	%	PValue	Genes	List Total	Pop Hits	Pop Total	Fold Enrichment	Bonferroni	Benjamini	FDR
GOTERM_CC_DIRECT	GO:0010008~endosome membrane	8	40	4.3694e-09	IRAK1, IRAK2, TRAF6, LY96, IRAK4, TLR4, MYD88, TLR3	20	292	20795	28.4863	2.2721e-07	2.2721e-07	1.8351e-07
GOTERM_CC_DIRECT	GO:0009986~cell surface	7	35	1.9366e-05	IL1A, IRAK1, IRAK4, TLR4, TIRAP, MYD88, TLR2	20	662	20795	10.9943	0.00100651	0.000494874	0.000399706
GOTERM_CC_DIRECT	GO:0005886~plasma membrane	15	75	2.8550e-05	MSR1, LY96, IRAK4, TIRAP, TLR1, MUC1, IRAK1, IRAK2, TRAF6, TLR6, TLR5, TLR4, MYD88, TLR3, TLR2	20	5597	20795	2.78654	0.00148354	0.000494874	0.000399706
GOTERM_CC_DIRECT	GO:0043235~receptor complex	5	25	3.9532e-05	TLR1, LY96, TLR6, TLR4, TLR2	20	217	20795	23.9574	0.00205357	0.00051391	0.000415081
GOTERM_CC_DIRECT	GO:0031234~extrinsic component of cytoplasmic side of plasma membrane	3	15	7.1504e-05	TRAF6, TIRAP, MYD88	20	14	20795	222.804	0.00371143	0.00074364	0.000600633

Category	Term	Count	%	PValue	Genes	List Total	Pop Hits	Pop Total	Fold Enrichment	Bonferroni	Benjamini	FDR
GOTERM_CC_DIRECT	GO:0035354~Toll-like receptor 1-Toll-like receptor 2 protein complex	2	10	0.00182657	TLR1, TLR2	20	2	20795	1039.75	0.0906894	0.0135688	0.0109594
GOTERM_CC_DIRECT	GO:0035355~Toll-like receptor 2-Toll-like receptor 6 protein complex	2	10	0.00182657	TLR6, TLR2	20	2	20795	1039.75	0.0906894	0.0135688	0.0109594
GOTERM_CC_DIRECT	GO:0030670~phagocytic vesicle membrane	3	15	0.00276156	TLR1, TLR6, TLR2	20	86	20795	36.2703	0.133939	0.0179501	0.0144982
GOTERM_CC_DIRECT	GO:0046696~lipopolysaccharide receptor complex	2	10	0.0045605	LY96, TLR4	20	5	20795	415.9	0.211552	0.0262863	0.0212312
GOTERM_CC_DIRECT	GO:0005615~extracellular space	7	35	0.00505505	IL1A, MUC1, CXCL8, IL1B, CCL2, IRAK4, TLR3	20	1867	20795	3.89837	0.231665	0.0262863	0.0212312
GOTERM_CC_DIRECT	GO:0019897~extrinsic component of plasma membrane	2	10	0.0091013	IRAK4, MYD88	20	10	20795	207.95	0.378385	0.0430243	0.0347504
GOTERM_CC_DIRECT	GO:0045121~membrane raft	3	15	0.0156417	TLR1, TLR6, TLR2	20	211	20795	14.7832	0.55948	0.0677809	0.0547461
GOTERM_CC_DIRECT	GO:0005737~cytoplasm	11	55	0.0249931	MUC1, IRAK1, IRF3, IRAK2, TRAF6, IRAK4, TLR4, TIRAP, MYD88, TLR3, TLR2	20	6004	20795	1.90494	0.731836	0.0999724	0.080747
GOTERM_CC_DIRECT	GO:0005829~cytosol	10	50	0.0507666	IL1A, IRAK1, IRF3, IRAK2, IL1B, TRAF6, IRAK4, TIRAP, MYD88, TLR2	20	5713	20795	1.81997	0.933412	0.188562	0.1523
GOTERM_CC_DIRECT	GO:0005811~lipid droplet	2	10	0.095069	IRAK1, TRAF6	20	109	20795	19.078	0.994454	0.329572	0.266193

Table S3. Complete Gene Ontology molecular function (GO-MF) enrichment results for the prioritized candidate gene set, including term names, gene counts, adjusted P-values, fold enrichment, and associated genes.

Category	Term	Count	%	PValue	Genes	List Total	Pop Hits	Pop Total	Fold Enrichment	Bonferroni	Benjamini	FDR
GOTERM_MF_DIRECT	GO:0005149~interleukin-1 receptor binding	5	25	6.7369e-10	IL1A, IL1B, IRAK4, TLR5, MYD88	20	14	19253	343.804	6.4001e-08	6.4001e-08	5.4569e-08
GOTERM_MF_DIR	GO:0061809~NAD+ nucleosidase activity,	4	20	4.5260e-07	TLR1, TLR6, TLR4, TLR2	20	16	19253	240.662	4.2996e-05	2.1499e-05	1.8330e-05

Category	Term	Count	%	PValue	Genes	List Total	Pop Hits	Pop Total	Fold Enrichment	Bonferroni	Benjamini	FDR
ECT	cyclic ADP-ribose generating											
GOTERM_MF_DIRECT	GO:0004888~transmembrane signaling receptor activity	6	30	9.7181e-07	TLR1, TLR6, TLR5, TLR4, TLR3, TLR2	20	192	19253	30.0828	9.2318e-05	3.0774e-05	2.6239e-05
GOTERM_MF_DIRECT	GO:0038023~signaling receptor activity	6	30	2.2638e-06	TLR1, TLR6, TLR5, TLR4, TLR3, TLR2	20	228	19253	25.3329	0.00021504	5.3766e-05	4.5842e-05
GOTERM_MF_DIRECT	GO:0035663~Toll-like receptor 2 binding	3	15	5.5296e-06	TLR1, TLR6, TIRAP	20	4	19253	721.987	0.000525173	0.000105062	8.9579e-05
GOTERM_MF_DIRECT	GO:0001875~lipopolysaccharide immune receptor activity	3	15	1.3808e-05	LY96, TLR4, TLR2	20	6	19253	481.325	0.00131088	0.000218621	0.000186403
GOTERM_MF_DIRECT	GO:0042802~identical protein binding	10	50	1.8665e-05	TLR1, IRAK1, IRF3, TRAF6, TLR6, TLR4, TIRAP, MYD88, TLR3, TLR2	20	1777	19253	5.41728	0.00177164	0.000253313	0.000215983
GOTERM_MF_DIRECT	GO:0071723~lipopeptide binding	3	15	4.1326e-05	TLR1, TLR6, TLR2	20	10	19253	288.795	0.00391831	0.000490741	0.000418421
GOTERM_MF_DIRECT	GO:0035591~signaling adaptor activity	4	20	6.8690e-05	IRAK2, TRAF6, TIRAP, MYD88	20	82	19253	46.9585	0.00650449	0.000725057	0.000618207
GOTERM_MF_DIRECT	GO:0001540~amyloid-beta binding	4	20	7.6466e-05	MSR1, TLR6, TLR4, TLR2	20	85	19253	45.3012	0.00723819	0.000726423	0.000619371
GOTERM_MF_DIRECT	GO:0038187~pattern recognition receptor activity	3	15	0.000635047	TLR5, TLR3, TLR2	20	38	19253	75.9987	0.0585637	0.0054845	0.00467625
GOTERM_MF_DIRECT	GO:0001530~lipopolysaccharide binding	3	15	0.000739432	LY96, TLR4, TLR2	20	41	19253	70.4378	0.0678597	0.00585383	0.00499116
GOTERM_MF_DIRECT	GO:0005515~protein binding	20	100	0.00152977	MSR1, CXCL8, LY96, IRAK4, TIRAP, TLR1, IL1A, MUC1, IRAK1, IRF3, IRAK2, IL1B, TRAF6, CCL2, TLR6, TLR5, TLR4, MYD88, TLR3, TLR2	20	13690	19253	1.40636	0.135358	0.0111791	0.00953166
GOTERM_MF_DIRECT	GO:0004672~protein kinase activity	4	20	0.00310229	IRAK1, IRAK2, CCL2, IRAK4	20	303	19253	12.7083	0.255599	0.0210512	0.0179489
GOTERM_MF_DIRECT	GO:0035662~Toll-like receptor 4 binding	2	10	0.0039419	LY96, TIRAP	20	4	19253	481.325	0.312863	0.0249654	0.0212863

Category	Term	Count	%	PValue	Genes	List Total	Pop Hits	Pop Total	Fold Enrichment	Bonferroni	Benjamini	FDR
GOTERM_MF_DIRECT	GO:0035325~Toll-like receptor binding	2	10	0.00590733	MYD88, TLR2	20	6	19253	320.883	0.430423	0.0336211	0.0286665
GOTERM_MF_DIRECT	GO:0046982~protein heterodimerization activity	4	20	0.00601642	IRAK1, IRAK2, TLR6, TLR4	20	384	19253	10.0276	0.43633	0.0336211	0.0286665
GOTERM_MF_DIRECT	GO:0060090~molecular adaptor activity	3	15	0.0152827	IRAK2, TIRAP, MYD88	20	193	19253	14.9635	0.768473	0.0806584	0.0687719
GOTERM_MF_DIRECT	GO:0008009~chemokine activity	2	10	0.0491702	CXCL8, CCL2	20	51	19253	37.751	0.991687	0.245851	0.20962
GOTERM_MF_DIRECT	GO:0005102~signaling receptor binding	3	15	0.0542684	CCL2, TLR6, TLR4	20	384	19253	7.5207	0.995012	0.257775	0.219787

Table S4. Complete KEGG pathway enrichment results for the prioritized candidate gene set, including pathway names, gene counts, adjusted P-values, fold enrichment, and associated genes.

Category	Term	Count	%	PValue	Genes	List Total	Pop Hits	Pop Total	Fold Enrichment	Bonferroni	Benjamini	FDR
KEGG_PATHWAY	hsa04620:Toll-like receptor signaling pathway	15	75	3.8033e-24	CXCL8, LY96, IRAK4, TIRAP, TLR1, IRAK1, IRF3, IL1B, TRAF6, TLR6, TLR5, TLR4, MYD88, TLR3, TLR2	19	109	8534	61.8107	2.7384e-22	2.7384e-22	1.2931e-22
KEGG_PATHWAY	hsa05133:Pertussis	11	55	9.1947e-17	IL1A, CXCL8, IRAK1, IRF3, IL1B, TRAF6, LY96, IRAK4, TLR4, TIRAP, MYD88	19	78	8534	63.3428	7.9936e-15	3.3101e-15	1.5631e-15
KEGG_PATHWAY	hsa05417:Lipid and atherosclerosis	13	65	8.1916e-16	CXCL8, LY96, IRAK4, TIRAP, IRAK1, IRF3, IL1B, TRAF6, CCL2, TLR6, TLR4, MYD88, TLR2	19	216	8534	27.0327	5.5955e-14	1.9660e-14	9.2838e-15
KEGG_PATHWAY	hsa05152:Tuberculosis	12	60	8.5869e-15	TLR1, IL1A, IRAK1, IRAK2, IL1B, TRAF6, TLR6, IRAK4, TLR4, TIRAP, MYD88, TLR2	19	182	8534	29.6148	6.1551e-13	1.5456e-13	7.2988e-14
KEGG_PATHWAY	hsa05142:Chagas disease	10	50	1.6928e-13	CXCL8, IRAK1, IL1B, TRAF6, CCL2, TLR6, IRAK4, TLR4, MYD88, TLR2	19	103	8534	43.6076	1.2190e-11	2.4376e-12	1.1511e-12
KEGG_PATHWAY	hsa05132:Salmonella infection	12	60	3.0427e-13	CXCL8, IRAK1, IL1B, TRAF6, LY96, TLR6, IRAK4, TLR5, TLR4, TIRAP, MYD88, TLR2	19	251	8534	21.4737	2.1910e-11	3.6512e-12	1.7242e-12
KEGG_PATHWAY	hsa04936:Alcoholic liver disease	10	50	3.6869e-12	CXCL8, IRAK1, IRF3, IL1B, TRAF6, LY96, IRAK4, TLR4,	19	144	8534	31.1915	2.6546e-10	3.7923e-11	1.7908e-11

Category	Term	Count	%	PValue	Genes	List Total	Pop Hits	Pop Total	Fold Enrichment	Bonferroni	Benjamini	FDR
					TIRAP, MYD88							
KEGG_PATHWAY	hsa05171:Coronavirus disease - COVID-19	11	55	8.4891e-12	CXCL8, IRAK1, IRF3, IL1B, TRAF6, CCL2, IRAK4, TLR4, MYD88, TLR3, TLR2	19	238	8534	20.7594	6.1122e-10	7.6402e-11	3.6079e-11
KEGG_PATHWAY	hsa05161:Hepatitis B	10	50	1.1384e-11	CXCL8, IRAK1, IRF3, TRAF6, IRAK4, TLR4, TIRAP, MYD88, TLR3, TLR2	19	163	8534	27.5557	8.1962e-10	9.1069e-11	4.3005e-11
KEGG_PATHWAY	hsa04064:NF-kappa B signaling pathway	9	45	1.5848e-11	CXCL8, IRAK1, IL1B, TRAF6, LY96, IRAK4, TLR4, TIRAP, MYD88	19	105	8534	38.4992	1.1410e-09	1.1410e-10	5.3882e-11
KEGG_PATHWAY	hsa05140:Leishmaniasis	8	40	1.3031e-10	IL1A, IRAK1, IL1B, TRAF6, IRAK4, TLR4, MYD88, TLR2	19	79	8534	45.4843	9.3822e-09	8.5293e-10	4.0277e-10
KEGG_PATHWAY	hsa05135:Yersinia infection	9	45	1.4564e-10	CXCL8, IRAK1, IRF3, IL1B, TRAF6, CCL2, IRAK4, TLR4, MYD88	19	138	8534	29.2929	1.0486e-08	8.5500e-10	4.0375e-10
KEGG_PATHWAY	hsa05162:Measles	9	45	1.5437e-10	IL1A, IRAK1, IRF3, IL1B, TRAF6, IRAK4, TLR4, MYD88, TLR2	19	139	8534	29.0822	1.1115e-08	8.5500e-10	4.0375e-10
KEGG_PATHWAY	hsa05164:Influenza A	9	45	8.9327e-10	IL1A, CXCL8, IRF3, IL1B, CCL2, IRAK4, TLR4, MYD88, TLR3	19	173	8534	23.3666	6.4315e-08	4.5940e-09	2.1694e-09
KEGG_PATHWAY	hsa05168:Herpes simplex virus 1 infection	9	45	1.3385e-09	IRAK1, IRF3, IL1B, TRAF6, CCL2, IRAK4, MYD88, TLR3, TLR2	19	182	8534	22.2111	9.6372e-08	6.4248e-09	3.0339e-09
KEGG_PATHWAY	hsa05130:Pathogenic Escherichia coli infection	9	45	3.1874e-09	CXCL8, IRAK1, IL1B, TRAF6, IRAK4, TLR5, TLR4, TIRAP, MYD88	19	203	8534	19.9134	2.2949e-07	1.4343e-08	6.7732e-09
KEGG_PATHWAY	hsa05144:Malaria	6	30	4.5500e-08	CXCL8, IL1B, CCL2, TLR4, MYD88, TLR2	19	50	8534	53.8989	3.2760e-06	1.9271e-07	9.1001e-08
KEGG_PATHWAY	hsa04621:NOD-like receptor signaling pathway	8	40	6.0562e-08	CXCL8, IRF3, IL1B, TRAF6, CCL2, IRAK4, TLR4, MYD88	19	189	8534	19.012	4.3604e-06	2.4225e-07	1.1439e-07
KEGG_PATHWAY	hsa05145:Toxoplasmosis	7	35	7.2930e-08	IRAK1, TRAF6, LY96, IRAK4, TLR4, MYD88, TLR2	19	112	8534	28.0724	5.2509e-06	2.7636e-07	1.3051e-07
KEGG_PATHWAY	hsa05134:Legionellosis	6	30	8.1405e-08	CXCL8, IL1B, TLR5, TLR4, MYD88, TLR2	19	56	8534	48.1241	5.8612e-06	2.9306e-07	1.3839e-07
KEGG_PATHWAY	hsa05323:Rheumatoid arthritis	6	30	1.1748e-06	IL1A, CXCL8, IL1B, CCL2, TLR4, TLR2	19	95	8534	28.3679	8.4583e-05	4.0279e-06	1.9021e-06
KEGG_PATHWAY	hsa05170:Human immunodeficiency virus 1 infection	7	35	3.2553e-06	IRAK1, IRF3, TRAF6, IRAK4, TLR4, MYD88, TLR2	19	213	8534	14.7611	0.000234352	1.0654e-05	5.0309e-06
KEGG_PATHWAY	hsa05131:Shigellosis	7	35	8.2190e-06	CXCL8, IRF3, IL1B, TRAF6, TLR5, TLR4, MYD88	19	250	8534	12.5764	0.000591592	2.5729e-05	1.2150e-05
KEGG_PATHWAY	hsa05321:Inflammatory	5	25	9.2039e-	IL1A, IL1B, TLR5, TLR4, TLR2	19	66	8534	34.0271	0.0006624	2.7612e-05	1.3039e-

Category	Term	Count	%	PValue	Genes	List Total	Pop Hits	Pop Total	Fold Enrichment	Bonferroni	Benjamini	FDR
HWAY	bowel disease			06						62		05
KEGG_PATHWAY	hsa05235:PD-L1 expression and PD-1 checkpoint pathway in cancer	5	25	3.1615e-05	TRAF6, TLR4, TIRAP, MYD88, TLR2	19	90	8534	24.9532	0.00227371	9.1051e-05	4.2996e-05
KEGG_PATHWAY	hsa05169:Epstein-Barr virus infection	6	30	4.9407e-05	IRAK1, IRF3, TRAF6, IRAK4, MYD88, TLR2	19	204	8534	13.2105	0.00355111	0.000136821	6.4610e-05
KEGG_PATHWAY	hsa04010:MAPK signaling pathway	6	30	0.000305023	IL1A, IRAK1, IL1B, TRAF6, IRAK4, MYD88	19	300	8534	8.98316	0.0217255	0.000813394	0.000384103
KEGG_PATHWAY	hsa04657:IL-17 signaling pathway	4	20	0.000966017	CXCL8, IL1B, TRAF6, CCL2	19	95	8534	18.9119	0.0672209	0.00248404	0.00117302
KEGG_PATHWAY	hsa04933:AGE-RAGE signaling pathway in diabetic complications	4	20	0.0011539	IL1A, CXCL8, IL1B, CCL2	19	101	8534	17.7884	0.0797672	0.00286485	0.00135285
KEGG_PATHWAY	hsa05146:Amoebiasis	4	20	0.0012213	CXCL8, IL1B, TLR4, TLR2	19	103	8534	17.443	0.0842277	0.00293112	0.00138414
KEGG_PATHWAY	hsa04722:Neurotrophin signaling pathway	4	20	0.00189639	IRAK1, IRAK2, TRAF6, IRAK4	19	120	8534	14.9719	0.127742	0.00440453	0.00207992
KEGG_PATHWAY	hsa04217:Necroptosis	4	20	0.00421614	IL1A, IL1B, TLR4, TLR3	19	159	8534	11.2996	0.262289	0.00919885	0.0043439
KEGG_PATHWAY	hsa04145:Phagosome	4	20	0.00421614	MSR1, TLR6, TLR4, TLR2	19	159	8534	11.2996	0.262289	0.00919885	0.0043439
KEGG_PATHWAY	hsa04622:RIG-I-like receptor signaling pathway	3	15	0.00984215	CXCL8, IRF3, TRAF6	19	72	8534	18.7149	0.509409	0.0208422	0.00984215
KEGG_PATHWAY	hsa05163:Human cytomegalovirus infection	4	20	0.0111454	CXCL8, IRF3, IL1B, CCL2	19	226	8534	7.9497	0.553795	0.0229277	0.0111454
KEGG_PATHWAY	hsa04060:Cytokine-cytokine receptor interaction	4	20	0.0233115	IL1A, CXCL8, IL1B, CCL2	19	298	8534	6.02897	0.817006	0.046623	0.0233115
KEGG_PATHWAY	hsa05418:Fluid shear stress and atherosclerosis	3	15	0.0348856	IL1A, IL1B, CCL2	19	141	8534	9.55655	0.922433	0.067826	0.0348856
KEGG_PATHWAY	hsa04380:Osteoclast differentiation	3	15	0.0357971	IL1A, IL1B, TRAF6	19	143	8534	9.42289	0.927535	0.067826	0.0357971
KEGG_PATHWAY	hsa04932:Non-alcoholic fatty liver disease	3	15	0.0424352	IL1A, CXCL8, IL1B	19	157	8534	8.58263	0.955933	0.0781546	0.0424352
KEGG_PATHWAY	hsa05160:Hepatitis C	3	15	0.0434192	IRF3, TRAF6, TLR3	19	159	8534	8.47468	0.959078	0.0781546	0.0434192
KEGG_PATHWAY	hsa05167:Kaposi sarcoma-associated herpesvirus infection	3	15	0.0631082	CXCL8, IRF3, TLR3	19	196	8534	6.87487	0.990845	0.110824	0.0631082
KEGG_PATHWAY	hsa05143:African trypanosomiasis	2	10	0.0753025	IL1B, MYD88	19	37	8534	24.2788	0.996436	0.12909	0.0753025
KEGG_PATHWAY	hsa04940:Type I diabetes mellitus	2	10	0.0889326	IL1A, IL1B	19	44	8534	20.4163	0.998776	0.148687	0.0889326
KEGG_PAT	hsa05332:Graft-versus-host	2	10	0.0908642	IL1A, IL1B	19	45	8534	19.9626	0.99895	0.148687	0.090864

Category	Term	Count	%	PValue	Genes	List Total	Pop Hits	Pop Total	Fold Enrichment	Bonferroni	Benjamini	FDR
HWAY	disease											2

Table S5. Cell-type proportions were compared between the flare and remission groups using the Wilcoxon test. Raw P values, adjusted P values, formatted P values, and significance annotations are reported as generated by the original statistical output. ns indicates not significant.

Cell Type	Measure	Group 1	Group 2	P Value	Adjusted P Value	Formatted P Value	Significance	Statistical Method
B_cell	Proportion	Flare	Remission	1	1	1	ns	Wilcoxon
Neutrophils	Proportion	Flare	Remission	1	1	1	ns	Wilcoxon
Monocyte	Proportion	Flare	Remission	1	1	1	ns	Wilcoxon
Hepatocytes	Proportion	Flare	Remission	1	1	1	ns	Wilcoxon
Epithelial_cells	Proportion	Flare	Remission	1	1	1	ns	Wilcoxon
DC	Proportion	Flare	Remission	1	1	1	ns	Wilcoxon
BM	Proportion	Flare	Remission	1	1	1	ns	Wilcoxon
NK_cell	Proportion	Flare	Remission	1	1	1	ns	Wilcoxon
Myelocyte	Proportion	Flare	Remission	1	1	1	ns	Wilcoxon
HSC_-G-CSF	Proportion	Flare	Remission	1	1	1	ns	Wilcoxon
GMP	Proportion	Flare	Remission	1	1	1	ns	Wilcoxon
Embryonic_stem_cells	Proportion	Flare	Remission	1	1	1	ns	Wilcoxon
CMP	Proportion	Flare	Remission	1	1	1	ns	Wilcoxon
Platelets	Proportion	Flare	Remission	1	1	1	ns	Wilcoxon
Pro-Myelocyte	Proportion	Flare	Remission	1	1	1	ns	Wilcoxon
Pre-B_cell_CD34-	Proportion	Flare	Remission	1	1	1	ns	Wilcoxon
T_cells	Proportion	Flare	Remission	1	1	1	ns	Wilcoxon
Pro-B_cell_CD34+	Proportion	Flare	Remission	1	1	1	ns	Wilcoxon

Table S6A. Heterogeneity statistics for four MR-prioritized genes.

Exposure ID	Outcome ID	Outcome Trait	Exposure Trait	MR Method	Cochran's Q	Q d.f.	Q-test P Value	Gene Symbol	Ensembl Gene ID	Exposure ID Used in Analysis
eqtl-a-ENSG00000038945	ebi-a-GCST90018977	Serum uric acid levels || id:ebi-a-GCST90018977	ENSG00000038945 || id:eqtl-a-ENSG00000038945	MR Egger	1034.24	1217	0.999951	MSR1	ENSG00000038945	eqtl-a-ENSG00000038945
eqtl-a-ENSG00000038945	ebi-a-GCST90018977	Serum uric acid levels || id:ebi-a-GCST90018977	ENSG00000038945 || id:eqtl-a-ENSG00000038945	Inverse variance weighted	1067.18	1218	0.999259	MSR1	ENSG00000038945	eqtl-a-ENSG00000038945

Exposure ID	Outcome ID	Outcome Trait	Exposure Trait	MR Method	Cochran's Q	Q d.f.	Q-test P Value	Gene Symbol	Ensembl Gene ID	Exposure ID Used in Analysis
eqtl-a-ENSG00000169429	ebi-a-GCST90018977	Serum uric acid levels || id:ebi-a-GCST90018977	ENSG00000169429 || id:eqtl-a-ENSG00000169429	MR Egger	182.801	173	0.29014	CXCL8	ENSG00000169429	eqtl-a-ENSG00000169429
eqtl-a-ENSG00000169429	ebi-a-GCST90018977	Serum uric acid levels || id:ebi-a-GCST90018977	ENSG00000169429 || id:eqtl-a-ENSG00000169429	Inverse variance weighted	183.031	174	0.304478	CXCL8	ENSG00000169429	eqtl-a-ENSG00000169429
eqtl-a-ENSG00000136869	ebi-a-GCST90018977	Serum uric acid levels || id:ebi-a-GCST90018977	ENSG00000136869 || id:eqtl-a-ENSG00000136869	MR Egger	2685.28	1631	9.6286e-55	TLR4	ENSG00000136869	eqtl-a-ENSG00000136869
eqtl-a-ENSG00000136869	ebi-a-GCST90018977	Serum uric acid levels || id:ebi-a-GCST90018977	ENSG00000136869 || id:eqtl-a-ENSG00000136869	Inverse variance weighted	2685.96	1632	1.0819e-54	TLR4	ENSG00000136869	eqtl-a-ENSG00000136869
eqtl-a-ENSG00000150455	ebi-a-GCST90018977	Serum uric acid levels || id:ebi-a-GCST90018977	ENSG00000150455 || id:eqtl-a-ENSG00000150455	MR Egger	690.125	482	1.3645e-09	TIRAP	ENSG00000150455	eqtl-a-ENSG00000150455
eqtl-a-ENSG00000150455	ebi-a-GCST90018977	Serum uric acid levels || id:ebi-a-GCST90018977	ENSG00000150455 || id:eqtl-a-ENSG00000150455	Inverse variance weighted	754.875	483	2.7994e-14	TIRAP	ENSG00000150455	eqtl-a-ENSG00000150455

Table S6B. MR-PRESSO sensitivity results for four MR-prioritized genes.

Gene Symbol	Ensembl Gene ID	Exposure ID Used in Analysis	MR-PRESSO Global Test P Value	MR-PRESSO Distortion Test P Value	MR-PRESSO Outlier Count	MR-PRESSO Error Message
MSR1	ENSG00000038945	eqtl-a-ENSG00000038945	0.9995			
CXCL8	ENSG00000169429	eqtl-a-ENSG00000169429	0.3365			
TLR4	ENSG00000136869	eqtl-a-ENSG00000136869		0.974	1633	
TIRAP	ENSG00000150455	eqtl-a-ENSG00000150455		0.926	484	

Table S6C. MR-Egger intercept tests for horizontal pleiotropy.

Exposure ID	Outcome ID	Outcome Trait	Exposure Trait	Egger Intercept	SE	P Value	Gene Symbol	Ensembl Gene ID	Exposure ID Used in Analysis
eqtl-a-ENSG00000038945	ebi-a-GCST90018977	Serum uric acid levels || id:ebi-a-GCST90018977	ENSG00000038945 || id:eqtl-a-ENSG00000038945	-0.00105738	0.00018423	1.1982e-08	MSR1	ENSG00000038945	eqtl-a-ENSG00000038945

Exposure ID	Outcome ID	Outcome Trait	Exposure Trait	Egger Intercept	SE	P Value	Gene Symbol	Ensembl Gene ID	Exposure ID Used in Analysis
eqtl-a-ENSG00000169429	ebi-a-GCST90018977	Serum uric acid levels || id:ebi-a-GCST90018977	ENSG00000169429 || id:eqtl-a-ENSG00000169429	0.000302147	0.000648569	0.641896	CXCL8	ENSG00000169429	eqtl-a-ENSG00000169429
eqtl-a-ENSG00000136869	ebi-a-GCST90018977	Serum uric acid levels || id:ebi-a-GCST90018977	ENSG00000136869 || id:eqtl-a-ENSG00000136869	9.0307e-05	0.000140613	0.520809	TLR4	ENSG00000136869	eqtl-a-ENSG00000136869
eqtl-a-ENSG00000150455	ebi-a-GCST90018977	Serum uric acid levels || id:ebi-a-GCST90018977	ENSG00000150455 || id:eqtl-a-ENSG00000150455	0.00273209	0.000406272	4.9906e-11	TIRAP	ENSG00000150455	eqtl-a-ENSG00000150455

Table S6D. Integrated sensitivity summary for four MR-prioritized genes.

Gene Symbol	Ensembl Gene ID	Reported Exposure ID	Exposure ID Used in Analysis	Number of SNPs	IVW Beta	IVW SE	IVW P Value	Cochran's Q	Q d.f.	Q-test P Value	Egger Intercept	Egger Intercept SE	Egger Intercept P Value	Steiger P Value	Correct Causal Direction	MR-PRESSO Global Test P Value	MR-PRESSO Distortion Test P Value	MR-PRESSO Outlier Count
MSR1	ENSG00000038945	eqtl-a-ENSG00000038945	eqtl-a-ENSG00000038945	1219	0.00401724	0.000264246	3.3950e-52	1067.18	1218	0.999259	-0.00105738	0.00018423	1.1982e-08		TRUE	0.9995		
CXCL8	ENSG00000169429	eqtl-a-ENSG00000169429	eqtl-a-ENSG00000169429	175	0.0169595	0.0016242	1.5989e-25	183.031	174	0.304478	0.000302147	0.000648569	0.641896	0	TRUE	0.3365		
TLR4	ENSG00000136869	eqtl-a-ENSG00000136869	eqtl-a-ENSG00000136869	1633	0.00314592	0.000310395	3.8547e-24	2685.96	1632	1.0819e-54	9.0307e-05	0.000140613	0.520809		TRUE		0.974	1633
TIRAP	ENSG00000150455	eqtl-a-ENSG00000150455	eqtl-a-ENSG00000150455	484	0.00927395	0.000874962	3.0037e-26	754.875	483	2.7994e-14	0.00273209	0.000406272	4.9906e-11		TRUE		0.926	484

Table S6E. Steiger directionality tests for four MR-prioritized genes.

Exposure ID	Outcome ID	Exposure Trait	Outcome Trait	SNP R^2 for Exposure	SNP R^2 for Outcome	Correct Causal Direction	Steiger P Value	Gene Symbol	Ensembl Gene ID	Exposure ID Used in Analysis
eqtl-a-ENSG00000038945	ebi-a-GCST90018977	ENSG00000038945 || id:eqtl-a-ENSG00000038945	Serum uric acid levels || id:ebi-a-GCST90018977	9.53731	0.00369545	TRUE		MSR1	ENSG00000038945	eqtl-a-ENSG00000038945
eqtl-a-ENSG00000169429	ebi-a-GCST90018977	ENSG00000169429 || id:eqtl-a-ENSG00000169429	Serum uric acid levels || id:ebi-a-GCST90018977	0.34158	0.000867463	TRUE	0	CXCL8	ENSG00000169429	eqtl-a-ENSG00000169429
eqtl-a-	ebi-a-	ENSG00000136869	Serum uric acid	11.5035	0.0083107	TRUE		TLR4	ENSG000001	eqtl-a-

<table>
<tr><th>Exposure ID</th><th>Outcome ID</th><th>Exposure Trait</th><th>Outcome Trait</th><th>SNP R² for Exposure</th><th>SNP R² for Outcome</th><th>Correct Causal Direction</th><th>Steiger P Value</th><th>Gene Symbol</th><th>Ensembl Gene ID</th><th>Exposure ID Used in Analysis</th></tr>
<tr><td>ENSG00000136869</td><td>GCST90018977</td><td>|| id:eqtl-a- ENSG00000136869</td><td>levels || id:ebi-a- GCST90018977</td><td></td><td></td><td></td><td></td><td></td><td>36869</td><td>ENSG00000136869</td></tr>
<tr><td>eqtl-a- ENSG00000150455</td><td>ebi-a- GCST90018977</td><td>ENSG00000150455 || id:eqtl-a- ENSG00000150455</td><td>Serum uric acid levels || id:ebi-a- GCST90018977</td><td>1.61422</td><td>0.00271549</td><td>TRUE</td><td></td><td>TIRAP</td><td>ENSG00000150455</td><td>eqtl-a- ENSG00000150455</td></tr>
</table>

Table S7. Summary table of main noncovalent interactions of fucoidan-TLR4 (PLIP analysis, included interaction type, residues, key parameters and notes).

Ligand_ID	Interaction_Type	Residues	Key_Parameters	Notes
HET:E:1	Hydrogen bond	C:122 LYS	H-bond: 2.90 Å 128.1°	protein donor
HET:E:3	Hydrogen bond	A:319 THR; A:362 LYS	H-bond: 3.12-3.55 Å 113.5-126.5°	sidechain; protein donor
HET:E:6	Hydrogen bond	A:294 ASP	H-bond: 3.27 Å 142.8°	ligand donor
HET:E:7	Hydrogen bond; Salt bridge	C:120 SER; C:122 LYS	H-bond: 3.59 Å 104.1°; Salt bridge: 5.14 Å	sidechain; protein donor
HET:F:1	Hydrophobic; Hydrogen bond	A:526 ASN; A:504 SER; A:505 GLN; A:550 ASP	H-bond: 1.61-3.15 Å 131.0-159.7°; Hydrophobic: 3.80 Å	sidechain; ligand donor; protein donor
HET:G:1	Hydrophobic; Hydrogen bond	A:575 ASN; A:550 ASP; A:552 SER	H-bond: 1.43-3.19 Å 148.3-162.8°; Hydrophobic: 2.87 Å	sidechain; ligand donor; protein donor
HET:H:1	Hydrogen bond	D:122 LYS	H-bond: 2.92 Å 126.8°	protein donor
HET:H:3	Hydrogen bond; Salt bridge	B:296 TYR; B:362 LYS; B:341 LYS	H-bond: 2.42-3.11 Å 124.5-153.9°; Salt bridge: 4.11-5.02 Å	sidechain; protein donor; ligand donor
HET:H:4	Hydrogen bond	B:341 LYS	H-bond: 3.43 Å 101.1°	sidechain; protein donor
HET:H:5	Hydrogen bond	B:296 TYR	H-bond: 2.92 Å 132.9°	sidechain; ligand donor
HET:H:7	Hydrogen bond	D:120 SER	H-bond: 3.59 Å 104.4°	sidechain; protein donor
HET:I:1	Hydrophobic; Hydrogen bond	B:526 ASN; B:504 SER; B:505 GLN; B:528 SER	H-bond: 1.66-3.60 Å 104.7-146.0°; Hydrophobic: 3.80 Å	sidechain; protein donor
HET:J:1	Hydrophobic; Hydrogen bond; Salt bridge	B:575 ASN; B:552 SER; B:577 THR; B:606 ARG; B:550 ASP	H-bond: 1.42-3.20 Å 125.0-160.4°; Salt bridge: 3.33 Å; Hydrophobic: 2.97 Å	sidechain; protein donor

Table S8. Summary table of main noncovalent interactions with alginate-TLR4 ((PLIP analysis, included interaction type, residues, key parameters and notes).

Ligand_ID	Interaction_Type	Residues	Key_Parameters	Notes
HET:E:1	Hydrogen bond	C:122 LYS	H-bond: 2.90-3.03 Å 124.9-128.1°	protein donor; ligand donor
HET:E:3 + HET:E:7	Hydrogen bond	C:120 SER; A:296 TYR;	H-bond: 2.59-3.59 Å 104.1-	sidechain; protein donor;

Ligand_ID	Interaction_Type	Residues	Key_Parameters	Notes
		A:319 THR; A:362 LYS	136.2°	ligand donor
HET:E:5 + HET:E:6	Hydrogen bond	A:294 ASP	H-bond: 3.27 Å 142.8°	ligand donor
HET:F:1 + HET:F:2	Hydrogen bond	A:504 SER; A:505 GLN; A:526 ASN; A:550 ASP	H-bond: 1.87-2.74 Å 123.1-147.4°	sidechain; protein donor; ligand donor
HET:G:1 + HET:G:2	Hydrogen bond	A:550 ASP	H-bond: 2.21 Å 149.9°	sidechain; ligand donor
HET:H:1	Hydrogen bond	D:122 LYS	H-bond: 2.92-3.22 Å 123.8-141.7°	protein donor; ligand donor
HET:H:3 + HET:H:4 + HET:H:5 + HET:H:6 + HET:H:7	Hydrogen bond; Salt bridge	D:120 SER; B:294 ASP; B:296 TYR; B:341 LYS; B:362 LYS	H-bond: 2.15-3.59 Å 104.4-153.9°; Salt bridge: 4.16 Å	sidechain; protein donor; ligand donor
HET:I:1 + HET:I:2	Hydrogen bond	B:504 SER; B:505 GLN; B:526 ASN; B:528 SER; B:550 ASP	H-bond: 2.42-3.60 Å 104.7-153.0°	sidechain; protein donor; ligand donor
HET:J:1 + HET:J:2	Hydrogen bond	B:550 ASP; B:575 ASN; B:577 THR; B:606 ARG	H-bond: 1.99-3.20 Å 107.1-155.7°	sidechain; ligand donor; protein donor

Table S9. ADMET prediction results for Fucoidan (pkCSM model). This table shows the main pharmacokinetic (ADMET) prediction parameters of the candidate non-starch polysaccharides (Fucoidan). The prediction is completed using the pkCSM online tool, and the input is the canonical molecular structure generated from the corresponding chemical descriptors.

Property	Model Name	Predicted Value	Unit
Absorption	Water solubility	-2.892	Numeric (log mol/L)
Absorption	Caco2 permeability	-1.933	Numeric (log Papp in 10-6 cm/s)
Absorption	Intestinal absorption (human)	0	Numeric (% Absorbed)
Absorption	Skin Permeability	-2.735	Numeric (log Kp)
Absorption	P-glycoprotein substrate	Yes	Categorical (Yes/No)
Absorption	P-glycoprotein I inhibitor	No	Categorical (Yes/No)
Absorption	P-glycoprotein II inhibitor	No	Categorical (Yes/No)
Distribution	VDss (human)	-1.117	Numeric (log L/kg)
Distribution	Fraction unbound (human)	0.429	Numeric (Fu)
Distribution	BBB permeability	-5.234	Numeric (log BB)
Distribution	CNS permeability	-7.624	Numeric (log PS)
Metabolism	CYP2D6 substrate	No	Categorical (Yes/No)
Metabolism	CYP3A4 substrate	Yes	Categorical (Yes/No)
Metabolism	CYP1A2 inhibitior	No	Categorical (Yes/No)
Metabolism	CYP2C19 inhibitior	No	Categorical (Yes/No)

Property	Model Name	Predicted Value	Unit
Metabolism	CYP2C9 inhibitior	No	Categorical (Yes/No)
Metabolism	CYP2D6 inhibitior	No	Categorical (Yes/No)
Metabolism	CYP3A4 inhibitior	No	Categorical (Yes/No)
Excretion	Total Clearance	0.727	Numeric (log ml/min/kg)
Excretion	Renal OCT2 substrate	No	Categorical (Yes/No)
Toxicity	AMES toxicity	No	Categorical (Yes/No)
Toxicity	Max. tolerated dose (human)	0.517	Numeric (log mg/kg/day)
Toxicity	hERG I inhibitor	No	Categorical (Yes/No)
Toxicity	hERG II inhibitor	No	Categorical (Yes/No)
Toxicity	Oral Rat Acute Toxicity (LD50)	2.051	Numeric (mol/kg)
Toxicity	Oral Rat Chronic Toxicity (LOAEL)	0.931	Numeric (log mg/kg_bw/day)
Toxicity	Hepatotoxicity	No	Categorical (Yes/No)
Toxicity	Skin Sensitisation	No	Categorical (Yes/No)
Toxicity	T.Pyriformis toxicity	0.285	Numeric (log ug/L)
Toxicity	Minnow toxicity	6.187	Numeric (log mM)

Table S10. ADMET prediction results for Alginate (pkCSM model). This table shows the main pharmacokinetic (ADMET) prediction parameters of the candidate non-starch polysaccharides (Alginate). The prediction is completed using the pkCSM online tool, and the input is the canonical molecular structure generated from the corresponding chemical descriptors.

Property	Model Name	Predicted Value	Unit
Absorption	Water solubility	-2.891	Numeric (log mol/L)
Absorption	Caco2 permeability	-1.58	Numeric (log Papp in 10-6 cm/s)
Absorption	Intestinal absorption (human)	0	Numeric (% Absorbed)
Absorption	Skin Permeability	-2.735	Numeric (log Kp)
Absorption	P-glycoprotein substrate	Yes	Categorical (Yes/No)
Absorption	P-glycoprotein I inhibitor	No	Categorical (Yes/No)
Absorption	P-glycoprotein II inhibitor	No	Categorical (Yes/No)
Distribution	VDss (human)	-1.189	Numeric (log L/kg)
Distribution	Fraction unbound (human)	0.535	Numeric (Fu)
Distribution	BBB permeability	-3.632	Numeric (log BB)
Distribution	CNS permeability	-7.53	Numeric (log PS)
Metabolism	CYP2D6 substrate	No	Categorical (Yes/No)
Metabolism	CYP3A4 substrate	Yes	Categorical (Yes/No)
Metabolism	CYP1A2 inhibitior	No	Categorical (Yes/No)
Metabolism	CYP2C19 inhibitior	No	Categorical (Yes/No)
Metabolism	CYP2C9 inhibitior	No	Categorical (Yes/No)

Property	Model Name	Predicted Value	Unit
Metabolism	CYP2D6 inhibitior	No	Categorical (Yes/No)
Metabolism	CYP3A4 inhibitior	No	Categorical (Yes/No)
Excretion	Total Clearance	1.187	Numeric (log ml/min/kg)
Excretion	Renal OCT2 substrate	No	Categorical (Yes/No)
Toxicity	AMES toxicity	No	Categorical (Yes/No)
Toxicity	Max. tolerated dose (human)	0.566	Numeric (log mg/kg/day)
Toxicity	hERG I inhibitor	No	Categorical (Yes/No)
Toxicity	hERG II inhibitor	No	Categorical (Yes/No)
Toxicity	Oral Rat Acute Toxicity (LD50)	1.659	Numeric (mol/kg)
Toxicity	Oral Rat Chronic Toxicity (LOAEL)	1.457	Numeric (log mg/kg_bw/day)
Toxicity	Hepatotoxicity	No	Categorical (Yes/No)
Toxicity	Skin Sensitisation	No	Categorical (Yes/No)
Toxicity	T.Pyriformis toxicity	0.285	Numeric (log ug/L)
Toxicity	Minnow toxicity	8.312	Numeric (log mM)
